# Effects of metronidazole on the fecal microbiome and metabolome in healthy dogs

**DOI:** 10.1111/jvim.15871

**Published:** 2020-08-28

**Authors:** Rachel Pilla, Frederic P. Gaschen, James W. Barr, Erin Olson, Julia Honneffer, Blake C. Guard, Amanda B. Blake, Dean Villanueva, Mohammad R. Khattab, Mustafa K. AlShawaqfeh, Jonathan A. Lidbury, Jörg M. Steiner, Jan S. Suchodolski

**Affiliations:** ^1^ Gastrointestinal Laboratory, Department of Small Animal Clinical Sciences Texas A&M University College Station Texas USA; ^2^ Department of Veterinary Clinical Sciences School of Veterinary Medicine, Louisiana State University Baton Rouge Louisiana USA; ^3^ School of Electrical Engineering and Information Technology German‐Jordanian University Amman Jordan

**Keywords:** antibiotic, bile acid metabolism, dysbiosis, fecal metabolome, microbiota, serum metabolome

## Abstract

**Background:**

Metronidazole has a substantial impact on the gut microbiome. However, the recovery of the microbiome after discontinuation of administration, and the metabolic consequences of such alterations have not been investigated to date.

**Objectives:**

To describe the impact of 14‐day metronidazole administration, alone or in combination with a hydrolyzed protein diet, on fecal microbiome, metabolome, bile acids (BAs), and lactate production, and on serum metabolome in healthy dogs.

**Animals:**

Twenty‐four healthy pet dogs.

**Methods:**

Prospective, nonrandomized controlled study. Dogs fed various commercial diets were divided in 3 groups: control group (no intervention, G1); group receiving hydrolyzed protein diet, followed by metronidazole administration (G2); and group receiving metronidazole only (G3). Microbiome composition was evaluated with sequencing of 16S rRNA genes and quantitative polymerase chain reaction (qPCR)‐based dysbiosis index. Untargeted metabolomics analysis of fecal and serum samples was performed, followed by targeted assays for fecal BAs and lactate.

**Results:**

No changes were observed in G1, or G2 during diet change. Metronidazole significantly changed microbiome composition in G2 and G3, including decreases in richness (*P* < .001) and in key bacteria such as Fusobacteria (*q* < 0.001) that did not fully resolve 4 weeks after metronidazole discontinuation. Fecal dysbiosis index was significantly increased (*P* < .001). Those changes were accompanied by increased fecal total lactate (*P* < .001), and decreased secondary BAs deoxycholic acid and lithocholic acid (*P* < .001).

**Conclusion and Clinical Importance:**

Our results indicate a minimum 4‐week effect of metronidazole on fecal microbiome and metabolome, supporting a cautious approach to prescription of metronidazole in dogs.

**Abbreviations**
ANOSIM: analysis of similarity
BA: bile acid
CA: cholic acid
CDCA: chenodeoxycholic acid
DCA: deoxycholic acid
DI: fecal dysbiosis index
LCA: lithocholic acid
PCoA: principal coordinate analysis
qPCR: quantitative polymerase chain reaction
SCFA: short‐chain fatty acids

## 1 INTRODUCTION

Intestinal microbiota and their metabolites are important in health. The microbiota primes the immune system and protects from enteropathogens.^1^ Bacteria‐derived metabolites, such as short‐chain fatty acids [SCFAs] are energy sources, regulate intestinal motility, and are anti‐inflammatory.^2^ Other metabolites include indole,^3^ a by‐product of tryptophan degradation, and secondary bile acids (BAs).^4^, ^5^ These metabolites are also immunomodulatory, thereby strengthening the intestinal barrier. Some important taxa (eg, *Ruminococcus*, *Faecalibacterium*) are depleted in dogs with chronic inflammatory enteropathies and acute colitis,^6^, ^7^, ^8^, ^9^, ^10^, ^11^ suggesting that these groups could be important in maintaining intestinal homeostasis. Therapeutic modulation of the microbiota is therefore a desirable approach in animals with gastrointestinal disease.^12^, ^13^

Antimicrobials are used empirically for treatment of both acute and chronic gastrointestinal disease. Antimicrobials, however, can disrupt the intestinal microbiome for a prolonged period of time. In humans, 30% of bacterial taxa were affected up to 6 months after antimicrobial administration.^14^ In healthy dogs, tylosin administration altered the jejunal microbiome, with some bacterial groups being decreased for more than 14 days.^15^ Tylosin increased fecal dysbiosis index (DI) and decreased the abundance of several key bacteria, including *Clostridium hiranonis*,^16^ a bacterial species responsible for BA conversion. Eight weeks after discontinuation of administration of tylosin several species were still decreased, and BA dysmetabolism was observed in some patients.^16^ Similarly, administration of metronidazole to healthy dogs led to major but reversible alterations in the intestinal microbiome.^17^

Metronidazole is the most prescribed antimicrobial for treatment of acute diarrhea in dogs, mostly due to suspicion of *Giardia* or *Clostridium perfringens* infection.^18^ Indeed, *C. perfringens* is suggested as the causal agent of acute hemorrhagic diarrhea syndrome in dogs, because of the strong association with the *netF* toxin gene.^19^ However, in a clinical trial there was no benefit of antimicrobial treatment.^20^

Metronidazole is commonly administered after dogs with chronic diarrhea fail 1 or more dietary trials,^21^, ^22^ including hydrolyzed protein diets. However, concerns over the use of antimicrobials, which generate dysbiosis, in dogs with an already dysbiotic microbiome have been raised, and alternative approaches with probiotics and synbiotics have been proposed.^22^, ^23^, ^24^, ^25^ In addition, it is unknown if the administration of metronidazole to dogs receiving a hydrolyzed protein diet affects the microbiome composition or its function differently than dogs receiving other commercial diets.

Limited information is available on how antimicrobial‐induced dysbiosis affects the serum and fecal metabolome, especially in dogs. Better understanding of changes in the microbiome and functional bacterial‐derived metabolites due to antimicrobials is needed. Therefore, the aim of this study was to evaluate the impact of metronidazole administration, alone or in combination with a hydrolyzed protein diet, on the fecal microbiome and metabolome, BA metabolism, fecal lactate production, and on the serum metabolome of healthy dogs.

## 2 MATERIALS AND METHODS

### 2.1 Study population

The study protocol was reviewed and approved (14‐027) by the Institutional Animal Care and Use Committee (IACUC) at Louisiana State University (LSU).

**FIGURE 1:**
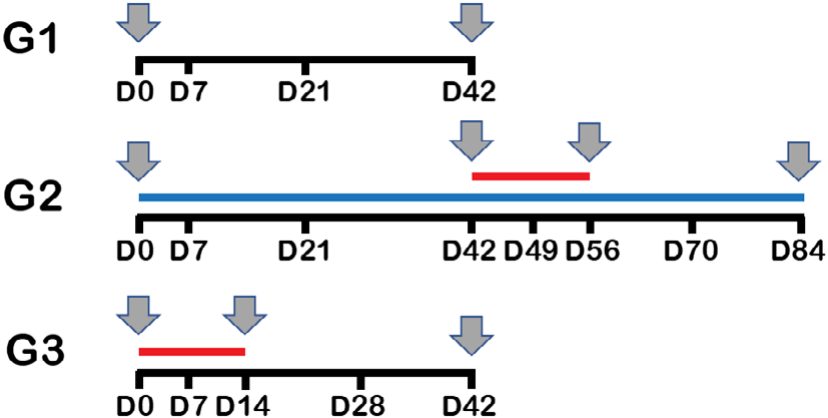
Schematic timeline. Dogs were randomly assigned into 3 groups (n = 8 each). Group 1 (controls) was maintained on their usual diet for the entire study period and did not receive any intervention. Group 2 (diet change/metronidazole) was switched to a soy‐based hydrolyzed protein diet (blue line) for a total of 6 weeks, after which they received metronidazole PO for 2 weeks (weeks 7 and 8, red line). Group 3 (metronidazole) was maintained on their usual diet for the entire study period, and received metronidazole at the same dose as dogs in group 2 for 2 weeks (weeks 1 and 2, red line). Fecal samples were collected at all time points; serum samples were obtained at the time points indicated with gray arrows

Twenty‐four clinically healthy staff owned dogs between the ages of 1 and 10 years old were enrolled. All dogs were deemed clinically healthy based on history (no signs of gastrointestinal disease and no antimicrobial treatment in the last 12 months) and abnormalities were not detected on physical examination, and CBC and serum chemistry panel. All dogs were owned by students or staff at the LSU School of Veterinary Medicine After receiving broad‐spectrum anthelminthic treatment (fenbendazole 50 mg/kg PO q24h for 3 days) they assigned to 1 of 3 groups (8 dogs each, Figure 1). Group 1 (controls) consisted of 8 dogs that were fed various diets, but that did not receive any intervention. Group 2 (diet change/metronidazole) consisted of 8 dogs that at enrollment were fed various diets, but were switched to a soy‐based hydrolyzed protein diet (HA Hydrolyzed, Canine Formula, Purina ProPlan Veterinary Diets) for a total of 6 weeks, after which they received metronidazole at 15 mg/kg PO q12h for 2 weeks (weeks 7 and 8). Group 3 (metronidazole) consisted of 8 dogs fed various diets, maintained on their usual diet for the entire study period, that received metronidazole at the same dose as dogs in group 2 for 2 weeks (weeks 1 and 2).

### 2.2 Collection of fecal and serum samples

Fecal samples were collected at various time points during the study (Figure 1), aliquoted in 1 g samples and frozen within 4 hours of collection and kept at −80°C until analysis. In group 1 (control), samples were collected at baseline (day 0), and on days 7, 21, and 42 to evaluate for any variation of the microbiome over 6 weeks without intervention.

In group 2 (diet change/metronidazole), fecal samples were collected to evaluate changes in microbiome and metabolome after dietary change for 6 weeks and during and after metronidazole administration. These samples were collected at day 0 (before diet switch), days 21 and 42 (3 and 6 weeks after diet switch and before metronidazole), days 49 and 56 (7 and 14 days of metronidazole administration), and days 70 and 84 (2 and 4 weeks after the cessation of metronidazole administration).

In group 3 (metronidazole), fecal samples were collected at baseline (day 0), days 7 and 14 (after 1 and 2 weeks on metronidazole) and days 28 and 42 (2 and 4 weeks after the cessation of metronidazole administration) to evaluate antimicrobial effects in a group of dogs on various diets.

Serum samples were collected (Figure 1) in group 1 (control dogs) on days 0, 21, and 42, in group 2 (diet change/metronidazole) on days 0, 21 and 42 (3 and 6 weeks after diet switch and before metronidazole), on day 56 (after 2 weeks of metronidazole administration), and on day 84 (4 weeks after the cessation of metronidazole administration), and in group 3 (metronidazole only) at baseline (day 0), on day 14 (2 weeks of metronidazole), and on day 42 (4 weeks after the cessation of metronidazole administration).

Blood samples were allowed to clot and centrifugated. The serum samples were then immediately frozen at ‐80°C until further analysis.

### 2.3 Serum markers

Serum concentrations of cobalamin and folate were measured using an automated chemiluminescence assay (Immulite2000, Siemens Healthcare Diagnostics).^26^

### 2.4 DNA extraction and sequencing of 16S rRNA genes

DNA was extracted from fecal samples using a MoBio Power soil DNA isolation kit (MoBio Laboratories) following the manufacturer's instructions. Illumina sequencing of the V4 region of the bacterial 16S rRNA genes was performed using primers 515F (5′‐GTGCCAGCMGCCGCGGTAA‐3′) to 806R (5′‐ GGACTACVSGGGTATCTAAT‐3″) at the MR DNA laboratory (www.mrdnalab.com, Shallowater, Texas) as previously described.^27^, ^28^, ^29^, ^30^ Briefly, the PCR reaction was performed in a single‐step 30 cycle PCR using the HotStarTaq Plus Master Mix Kit (Qiagen) under the following conditions: 94°C for 3 minutes, followed by 28 cycles (5 cycles used on PCR products) of 94°C for 30 seconds, 53°C for 40 seconds and 72°C for 1 minute, after which a final elongation step at 72°C for 5 minutes was performed. Using Illumina TruSeq DNA's protocol, a DNA library was set up and Illumina MiSeq was used for sequencing according the manufacturer's guidelines. Sequences were analyzed using a QIIME 2^31^ 2018.8 pipeline as described elsewhere.^32^, ^33^ The amplicon sequence variant (ASV) table was created using DADA2,^34^ and rarefied to 19 200 sequences per sample based on the lowest read depth in all samples for even depth of analysis. The raw sequences were uploaded to NCBI Sequence Read Archive under accession number SRP 066795.

Alpha diversity metrics were assessed by Chao1 (richness), observed ASVs (species richness), and Shannon diversity (evenness). Beta diversity was evaluated with the phylogeny based weighted UniFrac distance metric and plots were visualized using Principal Coordinate Analysis (PCoA).^35^ Analysis of similarity (ANOSIM) test within PRIMER 6 software package (PRIMER‐E Ltd., Luton, UK) was used to analyze significant differences in microbial communities between time points.

### 2.5 Quantitative PCR analysis and calculation of DI

To calculate a quantitative PCR (qPCR)‐based DI, qPCR assays were performed for total bacteria, *Faecalibacterium*, *Turicibacter*, *Escherichia coli*, *Streptococcus*, *Blautia*, *Fusobacterium*, and *C. hiranonis* as previously described.^36^, ^37^ Also, a probe‐based PCR assay was performed for *C. perfringens* as previously described.^38^

To correlate the results from the DI with the results from sequencing of 16S rRNA genes, a weighted UniFrac distance matrix was created with samples from all 3 groups. DI values were classified as normal (DI < 0), equivocal (0 < DI < 2), or high (DI > 2), and ANOSIM was calculated with the PRIMER 6 software package (PRIMER‐E Ltd.). In addition, the number of Observed ASVs and DI results from all 3 groups were used to calculate a Pearson correlation using GraphPad Prism 8.2.1 for Windows (GraphPad Software, San Diego, California).

### 2.6 Untargeted serum and fecal metabolomics

The serum and fecal metabolome were assessed using an untargeted approach at the West Coast Metabolomics Center (University of California, Davis, California) via gas chromatography time‐of‐flight mass spectrometry as previously described for canine serum and fecal samples.^29^, ^30^ Peak height data were obtained and uploaded to MetaboAnalyst 4.0 (Xia Lab, McGill University, Canada). Before statistical analysis the data were log transformed and Pareto scaled. Multivariate analysis (principal components analysis), and univariate analysis (1‐way analysis Of variance (ANOVA)) was then performed.

### 2.7 Fecal BA and lactate concentrations

Lyophilized fecal samples were used to measure the concentrations of unconjugated fecal primary BA (cholic acid [CA] and chenodeoxycholic acid [CDCA]) and secondary BA (lithocholic acid [LCA] and deoxycholic acid [DCA]) using a gas chromatography with mass spectrometry protocol previously described.^39^, ^40^, ^41^ Fecal concentrations of BA were expressed as μg/mg of lyophilized feces, as well as percentage of total BA.

Fecal concentrations of d‐, l‐, and total lactate were measured using a modified and adapted enzymatic assay (D‐/L‐Lactate Enzymatic Kit, R‐Biopharm) as described for canine fecal samples.^42^

### 2.8 Statistical analysis

All data sets were tested for normality using the Shapiro‐Wilk test (JMP Pro 11, SAS software). Friedman tests were performed for data over time and adjusted for multiple comparison using Benjamini and Hochberg's False Discovery Rate^43^ at each taxonomic level, alpha diversity parameters, qPCR data, dysbiosis index, BA measurements, and lactate concentrations, using GraphPad Prism version 8.2.1 for Windows (GraphPad Software). A *P* or *q* value <.05 was considered statistically significant. Post hoc Dunn's multiple comparison test was used to determine the bacterial taxa that were different between the time‐points.

Two‐way ANOVA was used to compare alpha diversity parameters between groups 2 and 3 during the metronidazole trial using GraphPad Prism 8.2.1 for Windows (GraphPad Software).

To establish the correlation between *C. hiranonis* and concentrations of secondary BAs, Pearson correlation was calculated using GraphPad Prism 8.2.1 for Windows (GraphPad Software).

## 3 RESULTS

### 3.1 Study population

The signalment of dogs is summarized in Table S1.

Of the 16 dogs enrolled in groups 2 and 3, owners of 9 (56%; 95% confidence interval = 29.9%‐80.2%) dogs reported the development of diarrhea during administration of metronidazole. Because this was an unexpected adverse event, fecal scores were not collected. However, diarrhea was described as yellow and varied from soft to watery, was likely of small bowel origin, started 2 to 3 days after the initiation of metronidazole and resolved within 2 to 3 days of the discontinuation of administration of metronidazole.

### 3.2 Serum markers

There were no significant changes in serum folate or cobalamin concentrations in any of the groups over time (*P* = .39 and *P* = .15, respectively).

### 3.3 Analysis of 16S rRNA genes

#### 3.3.1 Diversity within samples

**FIGURE 2:**
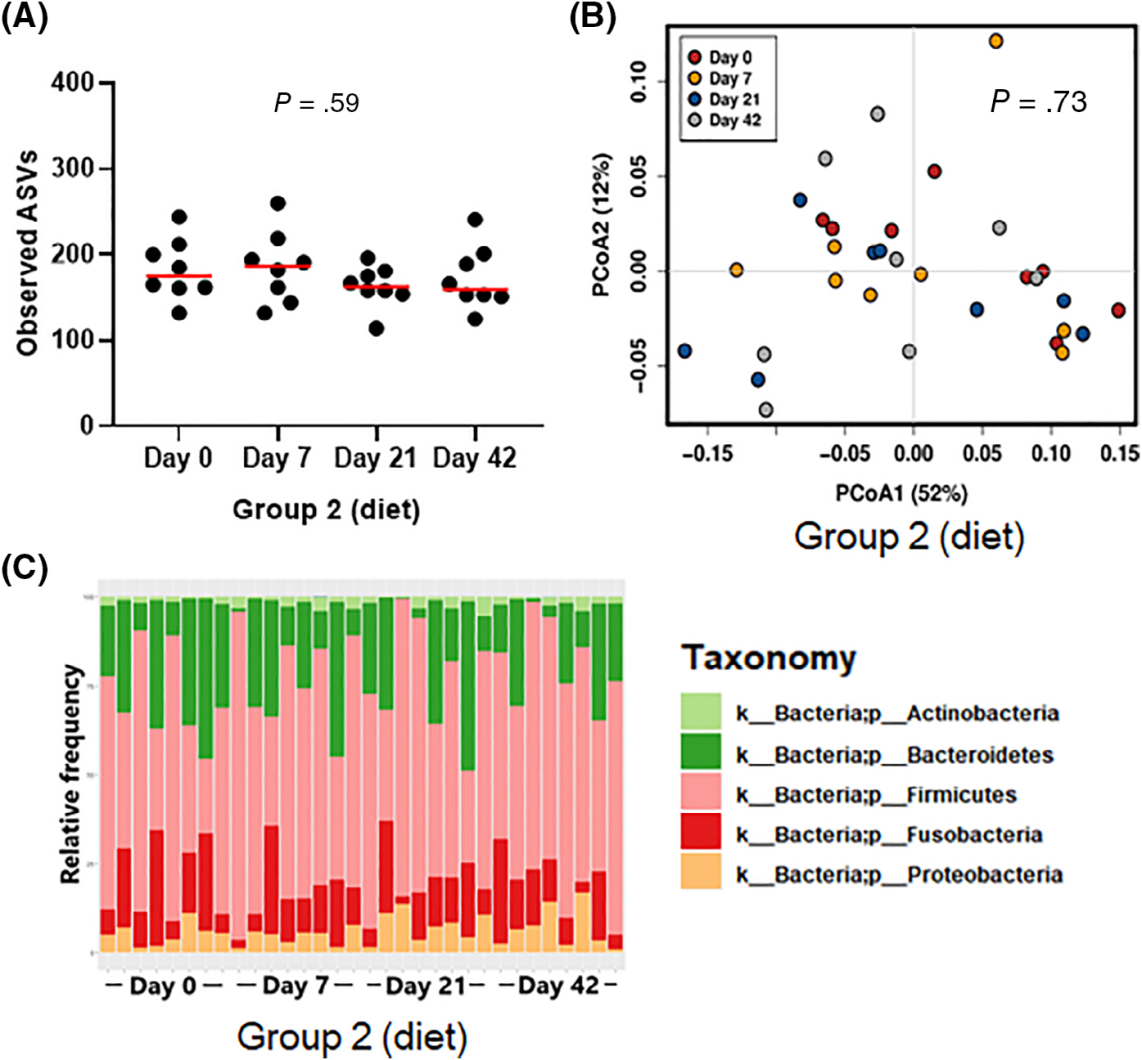
Species richness (A), PCoA of weighted UniFrac distances of taxa (B), and phylum bar graph for group 2 (fed hydrolyzed protein diet for 6 weeks before metronidazole trial, n = 8) during dietary trial. No significant difference was observed in (A) species richness (observed ASVs), (B) beta‐diversity, or (C) overall phylum abundances after diet change. ASVs, amplicon sequence variants; PCoA, principal coordinate analysis

Alpha‐diversity, or diversity within samples, did not change significantly over time in the control group (Chao1 *P* = .58, Observed ASVs *P* = .42, Shannon index *P* = .08; Figure S1A) or in group 2 during the dietary switch period (Chao1 *P* = .58, Observed ASVs *P* = .59, Shannon index *P* = .79; Figures 2A and S1B).

However, during metronidazole administration both in group 2 during the antimicrobial period, and in group 3, there was a significant decrease in all alpha‐diversity variables. Group 2 (Figure S1C) had significantly reduced richness after 14 days of metronidazole (Chao1 and Observed ASVs, *P* = .04 for both), and reduced evenness both at 7 and 14 days (Shannon index, *P* = .04 for both). In group 3 (Figure S1D), both richness and evenness were significantly decreased at day 7 (Chao1 *P* = .01, Observed ASVs *P* = .01, Shannon index *P* = .002) and at day 14 (Chao1 *P* = .03, Observed ASVs *P* = .03, Shannon index *P* = .001). Species richness increased after the antimicrobial was withdrawn in both group 2 and group 3, and was no longer significantly different from baseline after 2 and 4 weeks from the end of metronidazole administration.

**FIGURE 3:**
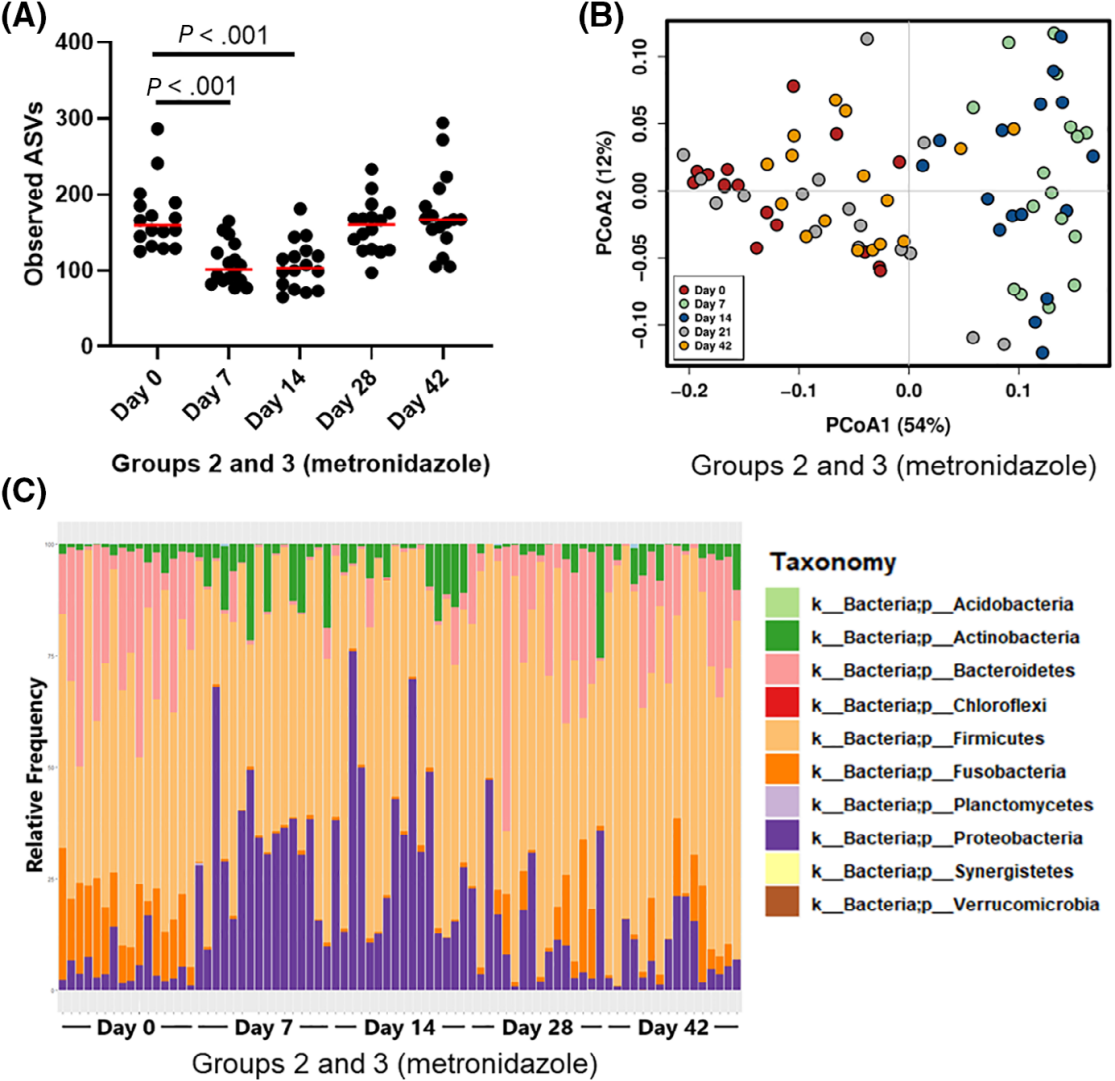
Species richness (A), PCoA of weighted UniFrac distances of taxa (B), and phylum bar graph for groups 2 (fed hydrolyzed protein diet for 6 weeks before metronidazole trial, n = 8) and 3 (maintained in various commercial diets, n = 8) during metronidazole trial. (A) Species richness (observed ASVs) was significantly decreased by metronidazole administration (days 7 and 14) but recovered after the discontinuation of metronidazole administration. (B) Beta‐diversity: red dots represent baseline samples. After 7 (green) and 14 (blue) days of metronidazole administration, microbial communities were significantly shifted (ANOSIM, *P* = .001 for both). Two (gray) and 4 (yellow) weeks after metronidazole was discontinued, samples clustered again with baseline samples; however, microbial communities remained significantly different from baseline (*P* = .02 and *P* = .01, respectively). (C) Phyla abundances are visibly altered during metronidazole administration (days 7 and 14), but return to baseline abundances 2 and 4 weeks after the end of metronidazole administration (days 28 and 42). ANOSIM, analysis of similarity; ASVs, amplicon sequence variants; PCoA, principal coordinate analysis

No difference was found in alpha‐diversity parameters in the response to metronidazole between group 2 (hydrolyzed protein diet) and group 3 (various commercial diets; Chao1 *P* = .86, Observed ASVs *P* = .86, Shannon index *P* = .26, Figure S1E), and therefore the results from both groups were combined in Figures 3A and S1F.

#### 3.3.2 Diversity between samples

Beta‐diversity, or diversity between samples, was evaluated through weighted UniFrac distance measures, and PCoA plots indicated no significant clustering of microbiome over time in group 1 (ANOSIM, *R* = −.03, *P* = .73; Figure S2A) and in group 2 before and after the dietary switch (ANOSIM, *R* = −.069, *P* = .95; Figures 2B and S2B).

However, significant changes were observed during the administration of metronidazole within group 2 (ANOSIM, *R* = .435, *P* = .001; Figure S2B) and within group 3 (ANOSIM, *R* = .49, *P* < .001; Figure S2C). Because both groups showed the same response to metronidazole up to day 21 (Figure S2D), the results from group 2 (hydrolyzed protein diet) and group 3 (various commercial diets) are shown combined in Figure 3B. Samples collected after 7 days of metronidazole were significantly different from baseline (ANOSIM, *R* = .944, *P* = .001), as were the samples after 14 days of metronidazole (ANOSIM, *R* = .872, *P* = .001). Samples collected 2 and 4 weeks after discontinuation of metronidazole administration clustered visually with the baseline samples; however, they remained statistically different from them (ANOSIM, day 28: *R* = .124, *P* = .02, day 42: *R* = .163, *P* = .01).

#### 3.3.3 Univariate statistics

Because no difference in diversity within and between samples was identified after administration of metronidazole in group 2 and group 3, the data for these groups were combined for univariate analysis of individual bacterial taxa. Figure S3 illustrates side‐by‐side the changes in group 1 (Figure S3A, control), group 2 (Figure S3B, diet change), and the groups that received metronidazole (Figure S3C, groups 2 and 3) at the phylum level.

No significant variance was observed in group 1 (Figure S3A, control). Median abundances and statistics for all taxonomic levels for group 1 are available as Supporting Information Data S1 (microbiome G1). Similarly, group 2 (Figures 2C and S3B, diet change) showed no significant variance during the dietary trial. Median abundances and statistics for all taxonomic levels for group 2 are available as Supporting Information Data S2 (microbiome G2 diet).

Metronidazole administration, instead, had a significant impact on the gut microbiome. As can be seen in Figure 3C and Supporting Information Data S3 (microbiome G2 and G3 metronidazole), Bacteroidetes and Fusobacteria abundance was significantly decreased from a median of 24.3 to 0.7% (*q* < 0.001) and 14.5 to 0.6% (*q* < 0.001), respectively, after 7 days of administration. Simultaneously, an increase in the abundance of Proteobacteria and Actinobacteria is seen, from 3.5% to 32.3% (*q* < 0.001) and 1.6% to 5.1% (*q* = 0.006), respectively, after 7 days. Two weeks after the end of administration (day 28), however, the abundances of all four phyla started to return to baseline levels, and by day 42 all but Fusobacteria (day 0 median: 14.49%, day 42 median: 1.8%, *q* = 0.025) were no longer significantly different from the abundances found before metronidazole administration.

While the abundance of phylum Firmicutes remained unchanged, its composition changed significantly (Supporting Information Data S3, microbiome G2 and G3 metronidazole). After 7 days of metronidazole administration, order Clostridiales was significantly reduced (from 47.5% to 8.9%, *q* < 0.001), and order Lactobacillales was significantly increased (from 1.3% to 42.5%, *q* < 0.001). Both orders returned to baseline abundances after 2 weeks from the end of administration (*q* > 0.999 for both).

### 3.4 qPCR and dysbiosis index

In line with the 16S rRNA sequencing data, only minor differences over time were observed in group 1 (Figure S4A). Abundance of *Blautia* and *C. hiranonis* was significantly increased (*P* = .008 and *P* = .02, respectively) at the end of the control period. The DI was not affected by those oscillations and remained unchanged.

During the diet change, group 2 also presented with minor differences that did not impact the DI values (Figure S4B). *Streptococcus* was found to significantly increase on day 42 (*P* = .001), and *E. coli* oscillated during the same period (overall *P* = .05).

**FIGURE 4:**
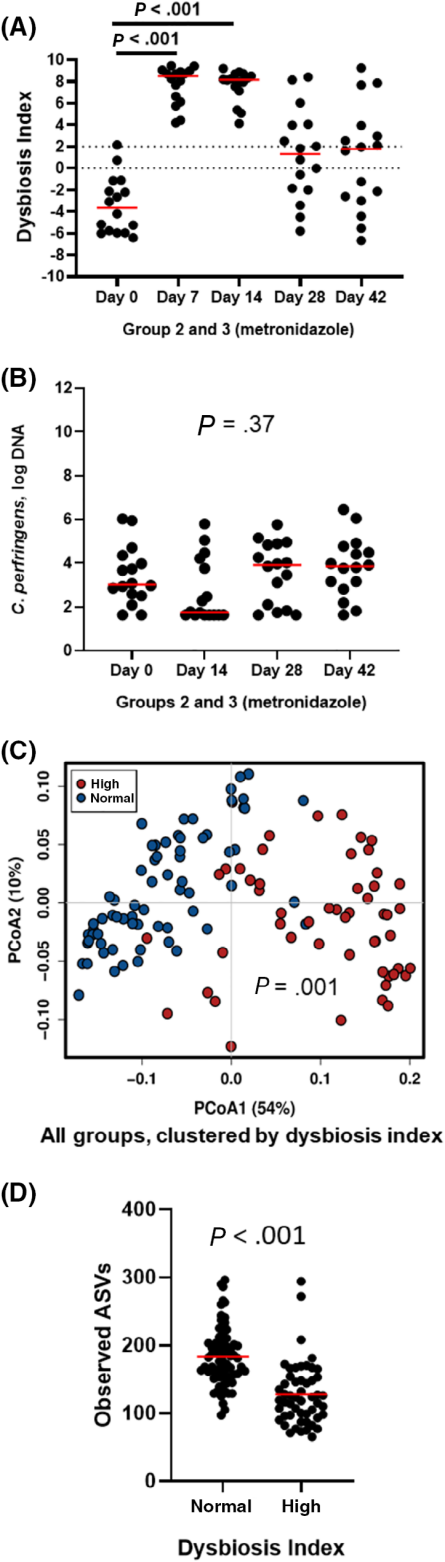
A, qPCR‐based dysbiosis index and, B, *Clostridium perfringens* abundance from groups 2 (fed hydrolyzed protein diet for 6 weeks before metronidazole trial, n = 8) and 3 (maintained in various commercial diets, n = 8) during metronidazole administration. C, PCoA of weighted UniFrac distances of taxa and, D, observed ASVs for all groups, clustered by dysbiosis index, are also shown. Dysbiosis index (A) is significantly increased (*P* < .001 for both) after 7 and 14 days of metronidazole administration, but no longer significantly different from baseline on days 28 and 42 (*P* = .74 and *P* > .99). Dotted lines indicate the reference interval: values below 0 indicate normobiosis, values between 0 and 2 are considered equivocal, and values above 2 indicate dysbiosis. Abundance of *Clostridium perfringens* (B) showed a trend towards reduction after 14 days of metronidazole (*P* = .37), but recovered after the end of administration. When clustered by DI (DI < 0 was considered normal, DI > 0 was considered high), samples with high DI (red) clustered separately from those with normal DI (blue) on a PCoA (weighted UniFrac, C), and showed lower richness (observed ASVs, D), showing that the qPCR‐based DI correlates well with sequencing results. ASVs, amplicon sequence variants; DI, dysbiosis index; PCoA, principal coordinate analysis; qPCR, quantitative polymerase chain reaction

Metronidazole administration in groups 2 and 3 increased the DI significantly (days 7 and 14, *P* < .001, Figure 4A). All but 1 bacterial taxa (*Blautia*) quantified by qPCR were significantly affected (Figure S4C), with decreased abundances of *Faecalibacterium*, *Turicibacter*, *Fusobacterium*, and *C. hiranonis*, and increased abundance of *Streptococcus* and *E. coli*. After 2 weeks from the end of metronidazole administration, the DI was already no longer significantly altered compared to baseline (day 28: *P* = .74, day 42: *P* > .99).

*Clostridium perfringens* was also quantified on samples from the metronidazole trial by qPCR. The abundance of *C. perfringens* was not significantly altered by administration of metronidazole (*P* = .37, Figure 4B).

When the correlation between the DI and the results of 16S rRNA gene sequencing was evaluated, beta‐diversity results from samples with normal or high DI were visually separately clustered (Figure 4C), and were significantly different (ANOSIM, *R* = .621, *P* = .001). When species richness (Observed ASVs) was considered, samples with normal DI values had significantly higher species richness (*P* < .001, Figure 4D), and DI values negatively correlated with species richness (*r* = −.572, *P* < .001).

### 3.5 Analysis of the fecal metabolome

A total of 215 named metabolites were detected in fecal samples. In group 1 and the dietary change period of group 2, no metabolites were significantly altered after adjustment for multiple comparisons. The complete list of metabolites, with mean values and statistics, are included as Supporting Information Data S4 (fecal metabolomics G1) and Supporting Information Data S5 (fecal metabolomics G2 dietary change period).

**FIGURE 5:**
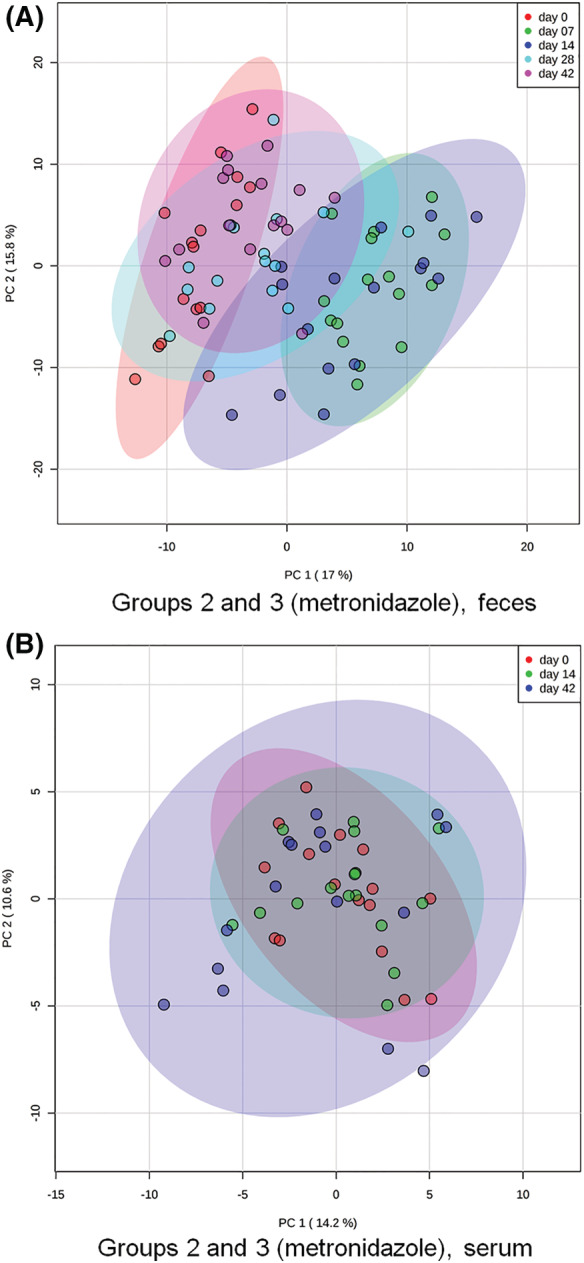
Principal coordinate analysis of, A, fecal and, B, serum metabolites for groups 2 (fed hydrolyzed protein diet for 6 weeks before metronidazole trial, n = 8) and 3 (maintained in various commercial diets, n = 8) during metronidazole trial. In figure (A), red dots represent baseline fecal samples. After 7 (green) and 14 (blue) days of metronidazole administration, the overall fecal metabolome composition was significantly altered. After 2 weeks (cyan blue, day 28) and 4 weeks (pink, day 42) from the end of administration, most fecal samples clustered again with baseline samples. In figure (B), red dots represent baseline serum samples. Overall, serum metabolome composition was not significantly affected by metronidazole administration (day 14, green), although a few outliers could be seen after 4 weeks from the end of administration (day 42, blue)

Metronidazole administration led to alteration in 87 measured fecal metabolites; 65 of those were still significantly altered after adjustment for multiple comparisons. Figure 5A shows on PCoA how samples from days 7 and 14 separate from baseline, but samples from days 28 and 42 once again cluster with samples from baseline (the figure includes data from dogs in groups 2 and 3 during and after metronidazole administration). While most of the changes were reversed 14 days after the end of antimicrobial administration, some of the evaluated metabolites remained significantly altered up to 4 weeks after the end of administration (end of study). The following metabolites decreased significantly: secondary BAs (LCA, *q* = 0.005; DCA, *q* = 0.002), vitamins (pantothenic acid, *q* = 0.044), nucleobases (uracyl, *q* < 0.001; thymidine, *q* = 0.003), and antioxidants (3,4‐dyhydroxyhydrocinnamic acid, *q* = 0.003). The complete list of metabolites, with mean values and statistics, is included as Supporting Information Data S6 (fecal metabolomics G2 and G3 metronidazole).

### 3.6 Analysis of the serum metabolome

A total of 146 named metabolites were identified in serum samples. In group 1 (control dogs), the serum metabolome was not changed over time. Also in group 2 after the dietary change, no metabolites were significantly altered after adjustment for multiple comparisons. The complete lists of metabolites, with mean values and statistics, are included as Supporting Information Data S7 (serum metabolomics G1) and Supporting Information Data S8 (serum metabolomics G2 diet).

During antimicrobial administration, only cholesterol (*q* = 0.042), isothreonic acid (*q* < 0.001), ribonic acid (*q* < 0.001), and ethanolamine (*q* = 0.001) were significantly altered after adjustment for multiple comparisons. Isothreonic acid, ribonic acid, and ethanolamine were increased after 2 weeks of metronidazole administration (day 14), but returned to baseline values after 4 weeks from the last antimicrobial dose. In contrast, cholesterol was not significantly altered during antimicrobial administration. However, after 4 weeks without metronidazole, it was significantly decreased. Figure 5B shows on PCoA how samples from days 14 and 42 did not separate from baseline values, reinforcing that changes observed were small. The complete list of metabolites, with mean values and statistics, is included as Supporting Information Data S9 (serum metabolomics G2 and G3 metronidazole).

### 3.7 Targeted assay for fecal BAs and fecal lactate

No significant changes in fecal BAs or fecal lactate concentrations were observed in group 1 (data not shown). During the dietary change trial of group 2, no differences were observed in fecal lactate (data not shown). However, a significant decrease in CA (*P* = .02) was observed on 7 day, likely driven by the normalization of an outlier (data not shown). No other difference was observed for the duration of the dietary trial.

**FIGURE 6:**
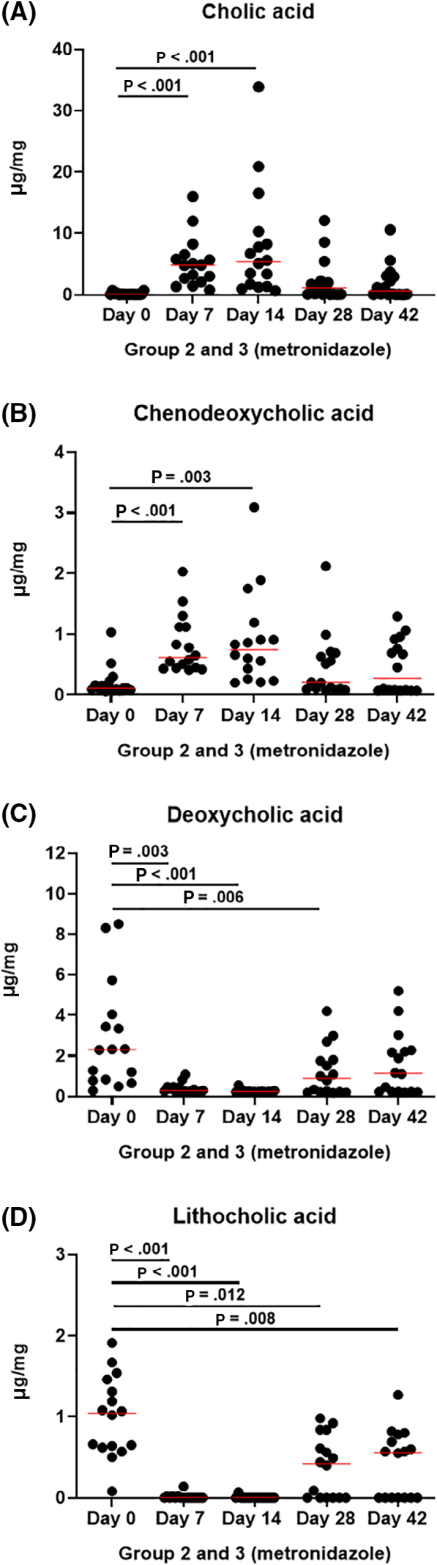
Bile acid quantification in fecal samples from groups 2 (fed hydrolyzed protein diet for 6 weeks before metronidazole trial, n = 8) and 3 (maintained in various commercial diets, n = 8) during metronidazole administration. Primary bile acids, (A) cholic acid, and (B) chenodeoxycholic acid, were increased during metronidazole administration (days 7 and 14), but returned to baseline values after the end of administration (days 28 and 42). In contrast, secondary bile acids (C) deoxycholic acid, and (D) lithocholic acid were decreased during metronidazole administration. After the end of administration secondary bile acids remaining decreased in some dogs

Administration of metronidazole significantly altered fecal BA composition, but not the fecal concentration of total BAs. The primary BAs cholic acid (Figure 6A) and CDCA (Figure 6B) were significantly increased on day 7 (*P* < .001 for both) and day 14 (*P* < .001 and *P* = .003, respectively). Both returned to baseline values after the end of administration. The secondary BAs deoxycholic acid (Figure 6C) and LCA (Figure 6D) were significantly decreased by metronidazole on day 7 (*P* = .003 and *P* < .001, respectively) and day 14 (*P* < .001 for both). However, recovery of secondary BAs was slower than that of primary BAs, with DCA remaining significantly decreased on day 28 (*P* = .006), and LCA still being significantly decreased on day 42 (*P* = .008).

**FIGURE 7:**
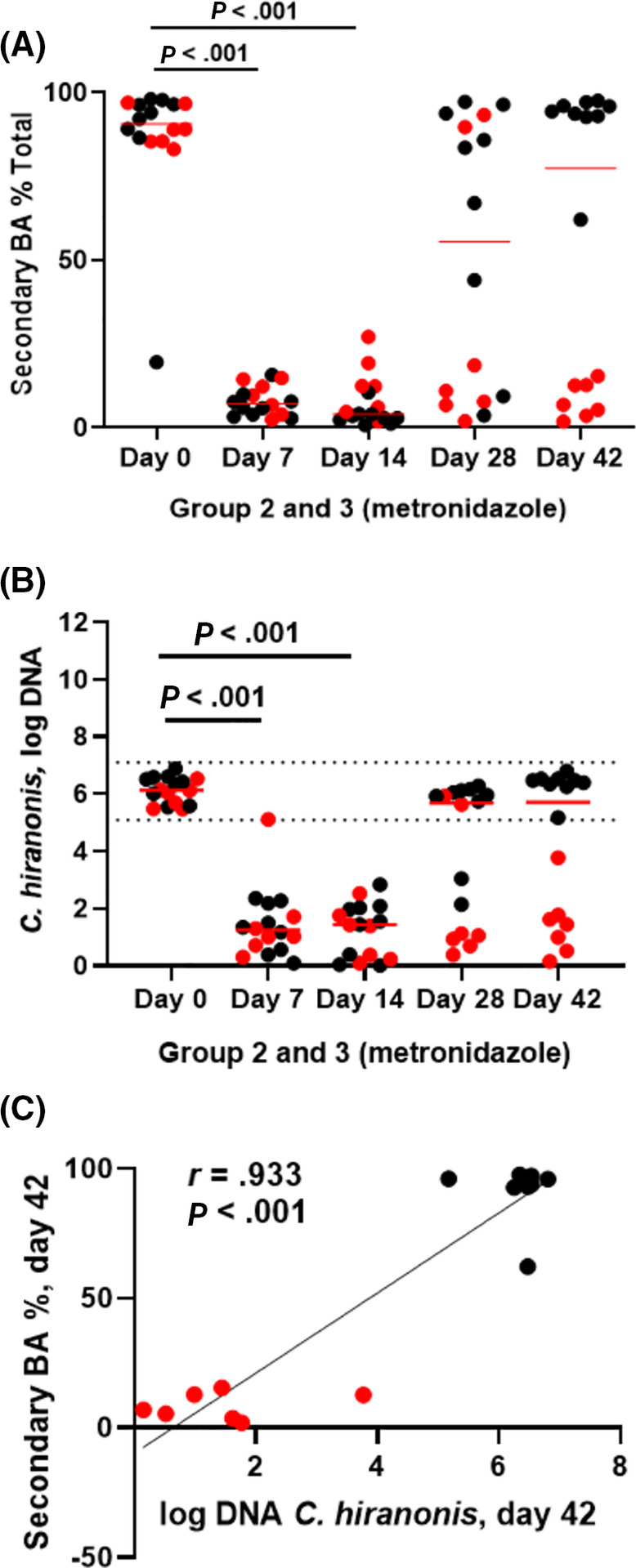
A, Secondary bile acid percentages, B, *Clostridium hiranonis* abundance, and, C, correlation between them on day 42 in fecal samples from groups 2 (fed hydrolyzed protein diet for 6 weeks before metronidazole trial, n = 8) and 3 (maintained in various commercial diets, n = 8) during metronidazole administration. In figure (A), secondary bile acid production was decreased during metronidazole administration, and did not recover after 4 weeks from the end of administration (day 42) in 7 dogs (highlighted in red). The same 7 dogs had a low abundance of *C. hiranonis* (B), which correlated with the percentage of secondary bile acids (C). Only 3/7 of these dogs developed diarrhea during metronidazole administration. Dotted lines (B) indicate the reference interval

The decrease of total secondary BAs can be seen in Figure 7A. Fecal secondary BAs normalized after the end of metronidazole administration 9 dogs (56%; Figure 7A, black dots), while it did not in 7 dogs (44%; Figure 7A, red dots). Because this pattern mirrored the abundance of *C. hiranonis* (Figure 7B), a Pearson correlation was calculated between total secondary BAs and *C. hiranonis* on day 42. Results indicated a strong correlation (*r* = .933, *P* < .001; Figure 7C).

**FIGURE 8:**
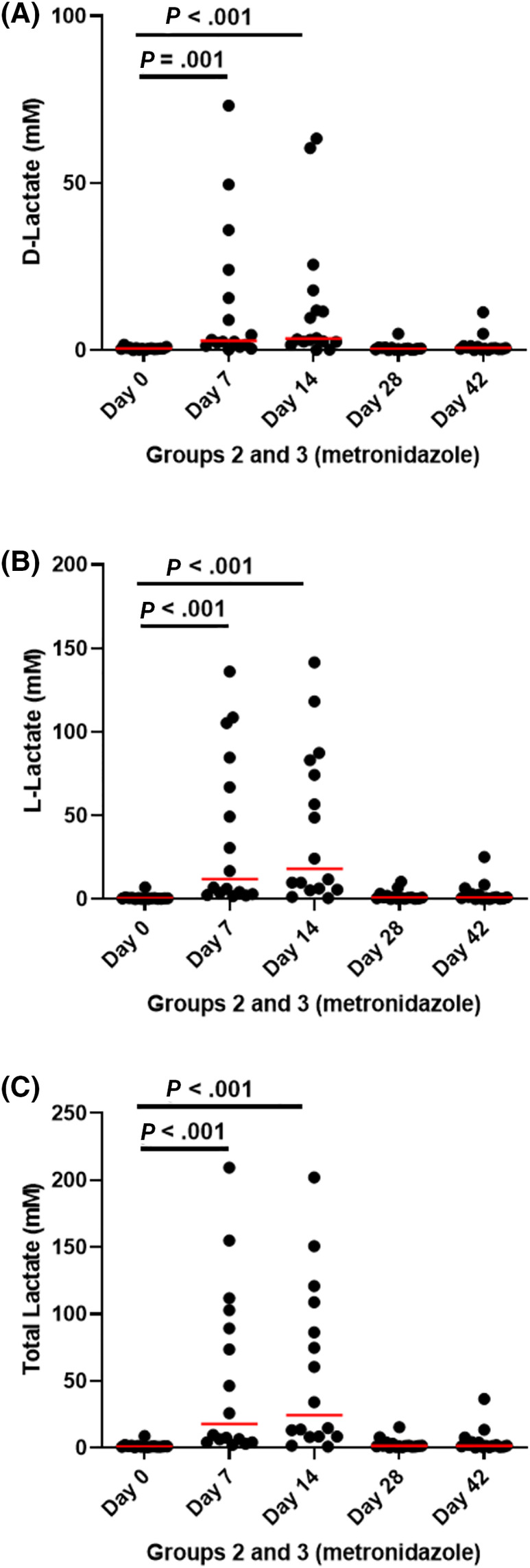
A, d‐lactate, B, l‐lactate, and, C, total lactate in fecal samples from groups 2 (fed hydrolyzed protein diet for 6 weeks before metronidazole trial, n = 8) and 3 (maintained in various commercial diets, n = 8) during metronidazole administration. Lactate was significantly increased during metronidazole administration, but returned to baseline levels after 2 weeks from the end of administration

In addition, metronidazole administration led to significant increase, compared to baseline, in d‐lactate (*P* = .001 and *P* < .001, respectively; Figure 8A), l‐lactate (*P* < .001 for both; Figure 8B), and total lactate (*P* < .001 for both; Figure 8C) on days 7 and 14. However, at the end of metronidazole administration, all 3 returned to baseline values and were no longer significantly different after 2 weeks (l‐lactate: *P* = .3, d‐lactate, and total lactate: *P* > .99).

## 4 DISCUSSION

The objective of this study was to describe the impact of metronidazole administration, alone or in combination with a hydrolyzed protein diet, on the fecal microbiome and metabolome, BA metabolism, fecal lactate production, and in the serum metabolome of a population of healthy dogs. Metronidazole had a significant impact, both alone and in combination with a hydrolyzed protein diet, in the fecal microbiome and metabolome. Microbiome composition was significantly altered, with decreased richness, and decreased abundance of Fusobacteria that did not fully recover after 4 weeks. The DI was significantly increased. The microbiome changes were accompanied by increased fecal lactate, increased markers of oxidative stress in feces and serum, and impaired BA conversion.

In the control group, we observed no significant differences in overall microbiome composition, richness, or evenness over time, nor changes in serum or fecal metabolites. When fed a hydrolyzed protein in the first half of the trial, dogs in group 2 also showed no significant differences in overall microbiome composition, richness, or evenness, nor in serum or fecal metabolites during the dietary trial. Hydrolyzed protein diets are considered 1 of the dietary choices for dogs with chronic enteropathies, and their advantages include reduced immunogenicity and increased digestibility.^44^ The lack of impact of this diet in the microbiome and metabolome of healthy dogs highlights its importance as a non microbiome‐damaging intervention for patients with chronic enteropathies, who already have dysbiosis.

After metronidazole administration, microbial communities were significantly changed. Antimicrobial administration caused a significant drop in richness (Chao1 and Observed ASVs) and evenness (Shannon index) on days 7 and 14 (Figures 3A and S1), which is consistent with previous findings with both metronidazole^17^ and tylosin^15^, ^16^ administration. In the PCoA plot (Figure 3B), overall microbial community composition was significantly shifted on days 7 and 14.

However, 2 weeks after the end of metronidazole administration (day 28), both evenness and richness had returned to baseline values. In addition, in the PCoA plot (Figure 3B), samples postadministration (days 28 and 42) clustered with those from preadministration, indicating that microbial communities did recover to a similar composition to baseline samples, as reported elsewhere.^17^

Metronidazole is an antimicrobial that selectively targets anaerobic bacteria. Indeed, metronidazole administration significantly decreased the abundance of mainly anaerobic Bacteroidetes and Fusobacteria phyla. In addition, and in agreement with the literature, abundances of Actinobacteria and Proteobacteria were significantly increased for the duration of administration.^17^ Even though the absolute abundance of the phylum Firmicutes was not altered, its composition changed significantly, with a decrease in anaerobic bacteria from order Clostridiales, and an increase of the order Lactobacillales, which includes aerobic and facultative anaerobic bacteria.^45^ Lactobacillales include known lactic acid‐producing genera, and their increase coincided with the increase in fecal lactate levels, a finding previously associated with chronic enteropathies and exocrine pancreatic insufficiency in dogs.^42^

Overall, the abundance of key SCFA‐producing bacteria was decreased during metronidazole administration. SCFAs are produced by bacteria mostly from dietary fiber, and are essential for the maintenance of intestinal health. As mentioned before, both SCFA concentrations, and abundances of SCFA‐producing bacteria are decreased in dogs with chronic enteropathies,^7^, ^46^ and are potential targets for treatment. Butyrate, in particular, is a source of energy for epithelial cells, and is responsible for intestinal homeostasis and regulation of gut permeability.

Butyrate producers are mostly anaerobes from the phylum Firmicutes,^47^, ^48^, ^49^ which were significantly decreased by metronidazole, including *Faecalibacterium prausnitzii*. Decreases in abundance of *F. prausnitzii* are associated with gastrointestinal disease in many species,^4^, ^50^ including dogs,^6^, ^10^, ^36^ which raises concerns over the long‐term impact of antimicrobial usage on gastrointestinal health. Phylum Bacteroidetes also contains genera known to produce SCFA, mainly acetate and propionate, and genus *Bacteroides* was significantly reduced by metronidazole (day 0:20.62% vs day 7: 0.6%). While in this study we did not measure SCFA concentrations, we can hypothesize that metronidazole administration decreases SCFA. Future studies that include this measurement are warranted.

A significant finding was the decrease of Fusobacteria after metronidazole administration (day 0:14.5% vs day 7: 0.6%). While Fusobacteria is associated with colon cancer in humans,^51^ in dogs, Fusobacteria seem to have an important role in the maintenance of health, and have been reported to be decreased in dogs with gastrointestinal disease.^36^, ^38^ Four weeks after the cessation of metronidazole (day 42), Fusobacteria abundance, comprising almost exclusively genus *Fusobacterium*, remained significantly reduced (day 0:14.49% vs day 42:1.8%, *P* = .03). While the role of *Fusobacterium* in the gastrointestinal tract in dogs is not fully understood, some Fusobacteria species are known to produce SCFA from protein sources, and might also have a role in BA conversion.

Bile acid metabolism is an essential function for intestinal health, and BA dysfunction has been associated with antimicrobial administration^16^ and chronic enteropathies in dogs.^41^, ^52^ Secondary BA production is a key function known to be impaired after antimicrobial administration in humans, and it is believed that lower secondary BA concentrations are a predisposing factor to antimicrobial‐induced *Clostridioides difficile* infections.^53^, ^54^ Fecal transplantation, 1 of the treatment options for recurring *C. difficile* infections is believed to work in part by restoring physiologic BA composition,^55^, ^56^ which is mainly attributed to *Clostridium scindens*.^57^ In dogs, colonization by *C. difficile* does not always correlate with clinical signs, and *C. difficile*‐induced diarrhea might be secondary to other underlying diseases.^58^, ^59^, ^60^, ^61^ However, the correlation between BA dysmetabolism and *C. difficile* colonization holds true, and protection from *C. difficile* seems to correlate with colonization by *C. hiranonis*,^62^ another bacterium with bile acid 7‐dehydroxylation ability.^63^ *Clostridium hiranonis* is part of the DI for dogs,^36^ and has been quantified in our study by qPCR. We observed that metronidazole administration significantly decreased *C. hiranonis* abundance, which did not recover in 7/16 dogs after 4 weeks from the end of administration of metronidazole.

Indeed, we observed both in untargeted fecal metabolomics and with a targeted quantitative assay that DCA and LCA, both secondary BAs produced by bacteria, were significantly decreased by metronidazole administration. A distinct separation of responses to the antimicrobial is visible when secondary BAs are considered together, as a percentage of total BAs, with 7/16 dogs showing persistently decreased secondary BAs 4 weeks after the end of metronidazole administration (Figure 7A, highlighted in red), mirroring the pattern seen in *C. hiranonis* abundance (Figure 7B), where the same 7 dogs were not recolonized with sufficient numbers of this bacterium 4 weeks after discontinuation of metronidazole. The correlation between the decreased secondary BAs and low *C. hiranonis* abundance 4 weeks after cessation of the antimicrobial administration was strong (Figure 7C), and suggests that metronidazole administration can have a long‐term impact on BA metabolism.

Untargeted metabolomics of fecal samples revealed that 65 metabolites were significantly impacted by metronidazole administration. In addition to secondary BAs, vitamins, nucleobases, and antioxidants were also significantly decreased after 4 weeks, indicating some long‐lasting gastrointestinal metabolic changes can be induced by antimicrobial administration.

Serum samples showed that the systemic impact of metronidazole administration was smaller than its gastrointestinal impact, as only 4 metabolites were affected. Cholesterol, isothreonic acid, ribonic acid, and ethanolamine were all significantly increased after 14 days of metronidazole administration, and returned to baseline values 4 weeks after the end of antimicrobial administration. While the increase was transient, the increase of isothreonic acid, a degradation product of ascorbic acid, and ribonic acid, a product of the oxidation of ribose, indicate that metronidazole administration increased systemic oxidative stress. Both compounds increase after traumatic brain injuries, another situation in which oxidative stress is increased.^64^, ^65^

There are limitations to our study that need to be considered. One of them is that our results were obtained in a cohort of healthy dogs, thus findings might not translate directly to dogs with gastrointestinal diseases. However, our results indicate that, despite its debatable benefit in improving clinical signs in dogs with diarrhea,^66^, ^67^ the effect of metronidazole is unlikely to be attributable to a normalization of the dysbiotic microbiome. Indeed, a clinical trial in dogs with acute diarrhea receiving metronidazole treatment for 7 days demonstrated that their microbiome was still dysbiotic after 28 days.^13^ Similar results have been reported in healthy dogs receiving tylosin for 7 days.^16^

During the 2 weeks of metronidazole administration, 9 of the 16 dogs developed diarrhea, as reported by their owners. Healthy dogs receiving tylosin in a previous study^16^ did not develop diarrhea during administration, despite showing a similar impact on *C. hiranonis* abundance and BA metabolism. No difference was observed in d‐lactate and l‐lactate at the end of administration between dogs that had diarrhea and those that did not (data not shown). Three out of 9 dogs that developed diarrhea were among the 7 dogs that had persistent dysbiosis and BA dysmetabolism. As this was an unanticipated occurrence, no fecal scores were collected; however, we suggest that further studies with antimicrobial administration should always include the evaluation of fecal scores, even in healthy dogs.

In addition, the small sample size could also be a limitation. No differences in the microbiome and metabolome of dogs from group 2 and group 3 were observed during and after metronidazole administration in microbiome diversity within or between samples. Therefore, to increase statistical power, we combined samples from groups 2 and 3 during and after the metronidazole administration. However, it is possible that small differences in individual bacterial taxa between those 2 groups might have been missed, and the impact of diets other than hydrolyzed protein diet remains unknown. Additionally, a cross‐over study design would have been ideal; however, due to the prolonged effects of antimicrobial administration, which are well documented here and in the literature,^16^ such a design would have been difficult to implement.

Overall, our results indicate that, while a dietary change to a hydrolyzed protein diet did not significantly impact the fecal microbiome of healthy dogs, metronidazole administration significantly changed the microbiome richness and composition, including a decrease in key bacteria, such as Fusobacteria and *C. hiranonis* that did not fully resolve 4 weeks after discontinuation of the antimicrobial administration. Those changes were reflected in a higher DI, increased fecal lactate, increased oxidative stress markers in feces and serum, and impaired BA conversion, which persisted for at least 4 weeks after the end of administration in almost half (7/16) of the dogs included in the trial. Our results point toward a long‐lasting effect of metronidazole administration, and should be considered as further evidence to support a more cautious approach to prescribing this antimicrobial to dogs.

## CONFLICT OF INTEREST DECLARATION

Rachel Pilla, Amanda B. Blake, Mohammad R. Khattab, Jonathan A. Lidbury, Jörg M. Steiner, and Jan S. Suchodolski are employed by the Gastrointestinal Laboratory at Texas A&M University, which provides assay for intestinal function and microbiota analysis on a fee‐for‐service basis. Frederic P. Gaschen, James W. Barr, Erin Olson, Julia Honneffer, Blake C. Guard, Dean Villanueva, and Mustafa K. AlShawaqfeh have no conflicts to declare.

## OFF‐LABEL ANTIMICROBIAL DECLARATION

Authors declare no off‐label use of antimicrobials.

## INSTITUTIONAL ANIMAL CARE AND USE COMMITTEE (IACUC) OR OTHER APPROVAL DECLARATION

Louisiana State University IACUC approval (14‐027).

## HUMAN ETHICS APPROVAL DECLARATION

Authors declare human ethics approval was not needed for this study.

## Supporting information


**Supplementary Data S1.** **List of relevant bacterial taxa detected in fecal samples from group 1 (control), separated by taxonomic level, with median and range for each time point.** Time points were compared with Friedman test, and adjusted for multiple comparison using Benjamini and Hochberg's False Discovery Rate, and p‐ and q‐values are presented. Post hoc Dunn's multiple comparison test was used to determine the bacterial taxa that were different between the time‐points, and significant differences are indicated by different superscript letters.

**Supplementary Data S2.** **List of relevant bacterial taxa detected in fecal samples from group 2 during the hydrolyzed protein diet trial, separated by taxonomic level, with median and range for each time point.** Time points were compared with Friedman test, and adjusted for multiple comparison using Benjamini and Hochberg's False Discovery Rate, and p‐ and q‐values are presented. Post hoc Dunn's multiple comparison test was used to determine the bacterial taxa that were different between the time‐points, and significant differences are indicated by different superscript letters.

**Supplementary Data S3.** **List of relevant bacterial taxa detected in fecal samples from groups 2 and 3 during the metronidazole trial, separated by taxonomic level, with median and range for each time point.** Time points were compared with Friedman test, and adjusted for multiple comparison using Benjamini and Hochberg's False Discovery Rate, and p‐ and q‐values are presented. Post hoc Dunn's multiple comparison test was used to determine the bacterial taxa that were different between the time‐points, and significant differences are indicated by different superscript letters.

**Supplementary Data S4.** **List of relevant metabolites detected in fecal samples from group 1 (control), with mean and SD for each time point.** Time points were compared with 1‐way ANOVA and adjusted for multiple comparison using Benjamini and Hochberg's False Discovery Rate, and p‐ and q‐values are presented.

**Supplementary Data S5.** **List of relevant metabolites detected in fecal samples from group 2 during the hydrolyzed protein diet trial, with mean and SD for each time point.** Time points were compared with 1‐way ANOVA and adjusted for multiple comparison using Benjamini and Hochberg's False Discovery Rate, and p‐ and q‐values are presented.

**Supplementary Data S6.** **List of relevant metabolites detected in fecal samples from groups 2 and 3 during the metronidazole trial, with mean and SD for each time point.** Time points were compared with 1‐way ANOVA and adjusted for multiple comparison using Benjamini and Hochberg's False Discovery Rate, and p‐ and q‐values are presented.

**Supplementary Data S7.** **List of relevant metabolites detected in serum samples from group 1 (control), with mean and SD for each time point.** Time points were compared with 1‐way ANOVA and adjusted for multiple comparison using Benjamini and Hochberg's False Discovery Rate, and p‐ and q‐values are presented.

**Supplementary Data S8.** **List of relevant metabolites detected in serum samples from group 2 during the hydrolyzed protein diet trial, with mean and SD for each time point.** Time points were compared with 1‐way ANOVA and adjusted for multiple comparison using Benjamini and Hochberg's False Discovery Rate, and p‐ and q‐values are presented.

**Supplementary Data S9.** List of relevant metabolites detected in serum samples from groups 2 and 3 during the metronidazole trial, with mean and SD for each time point. Time points were compared with 1‐way ANOVA and adjusted for multiple comparison using Benjamini and Hochberg's False Discovery Rate, and p‐ and q‐values are presented

**Supplementary Figure S1.** **Alpha diversity for (A) group 1 (control), (B) groups 2 (hydrolyzed protein diet trial), (C) group 2 (metronidazole trial), (D) group 3 (metronidazole trial), and (E) a comparison between groups 2 and 3 during the metronidazole trial.** Species richness (Chao 1 and Observed ASVs) and evenness (Shannon index) were not significantly affected by time (A) or diet change (B). Metronidazole administration significantly decreased all alpha diversity parameters in both group 2 (C) and 3 (D, days 7 and 14), but all parameters recovered after discontinuation of the antibiotic. No difference in the response to metronidazole was observed between group 2, which was concomitantly in a hydrolyzed diet, and group 3, which remained on their original diets (E).

**Supplementary Figure S2.** **Principal Coordinate Analysis (PCoA) of weighted UniFrac distances of 16S rRNA genes for (A) group 1 (control), (B) group 2 (metronidazole trial), (C) group 3 (metronidazole trial), and (D) ANOSIM comparison between groups 2 and 3 during metronidazole trial.** Microbial communities were not significantly affected by time (A, *P* = .73). Metronidazole administration, as shown on day 7 (green) and day 14 (blue), significantly shifted microbial communities in both group 2 (B, *P* = .001) and 3 (C, *P* < .001). After discontinuation of treatment, microbial communities shifted back towards baseline samples, indicating a recovery of the microbiome. (D) No difference in the response to metronidazole was observed between group 2 (B), which was concomitantly in a hydrolyzed diet, and group 3 (C), which remained on their original diets, before (day 0), during (days 7 and 14), or after (day 21) metronidazole administration. A significant difference was seen at day 42 (D).

**Supplementary Figure S3.** **Phylum bar graphs from (A) group 1, (B) group 2 during the hydrolyzed protein diet trial, An (C) groups 2 and 3 during the administration of metronidazole.** No significant variation is observed in 3A and 3B over time. In 3C, instead, metronidazole administration caused a significant change with decreased Bacteroidetes and Fusobacteria, and increased Proteobacteria and Actinobacteria on days 7 and 14. After the discontinuation of metronidazole, Bacteroidetes, Proteobacteria and Actinobacteria returned to abundances similar to baseline, but Fusobacteria remained significantly decreased (*P* = .025).

**Supplementary Figure S4.** **qPCR for *Faecalibacterium*, *Turicibacter*, *Streptococcus*, *E. coli*, *Blautia*, *Fusobacterium*, *Clostridium hiranonis*, and calculated fecal dysbiosis index for (A) group 1 (control), (B) group 2 during the hydrolyzed protein diet trial, and (C) groups 2 and 3 during the metronidazole trial.** Results are expressed in logDNA, and dotted lines indicate the reference intervals. Fecal dysbiosis index values below zero indicate normobiosis, values between 0 and 2 are considered equivocal, and values above 2 indicate dysbiosis.

**Supplementary Table S1.** Signalment of dogs enrolled
