# Supplementary material for: Effects of metronidazole on the fecal microbiome and metabolome in healthy dogs
Source: J Vet Intern Med. 2020 Aug 28;34(5):1853–66. doi: 10.1111/jvim.15871 (PMC7517498; doi:10.1111/jvim.15871)
Supplement: Supplementary file 1 — Supplementary Data S1. List of relevant bacterial taxa detected in fecal samples from group 1 (control), separated by taxonomic level, with median and range for each time point. Time points were compared with Friedman test, and adjusted for multiple comparison using Benjamini and Hochberg's False Discovery Rate, and p‐ and q‐values are presented. Post hoc Dunn's multiple comparison test was used to determine the bacterial taxa that were different between the time‐points, and significant differences are indicated by different superscript letters. [file JVIM-34-1853-s001.pdf]

PHYLUM

Bacterial group	Day 0		Day 7		Day 21		Day 42		Day 0 vs Day 7 vs Day 21 vs Day 42	
	Median	Range	Median	Range	Median	Range	Median	Range	P value	Q value
Actinobacteria	1.06	0.19-2.89	1.18	0.8-1.62	1.33	0.1-3.49	1.34	0-8.84	0.5828	0.7285
Bacteroidetes	23.14	13.71-38.3	23.73	14.24-39.9	24.62	15.33-41.43	13	0.84-32.46	0.3691	0.692167
Firmicutes	48.1	17.83-67.01	56.1	48.27-61.94	50.66	15.5-60.42	70.44	34.07-95.27	0.4153	0.692167
Fusobacteria	14.98	8.68-36.44	10.95	0.67-22.15	11.47	4.27-38.48	9.6	1.01-14.72	0.0979	0.4895
Proteobacteria	5.45	4.14-21.13	5.17	3.45-13.5	5.74	1.67-17.79	4.58	0.76-24.16	0.9852	0.9852

CLASS

Bacterial group	Day 0		Day 7		Day 21		Day 42		Day 0 vs Day 7 vs Day 21 vs Day 42	
	Median	Range	Median	Range	Median	Range	Median	Range	P value	Q value
Actinobacteria	0	0-0.15	0	0-0.21	0	0-0.4	0.02	0-4.99	0.2998	0.428286
Coriobacteriia	1	0.19-2.75	1.18	0.8-1.58	1.27	0.1-3.09	1.28	0-4.11	0.5828	0.647556
Bacteroidia	23.14	13.71-38.3	23.73	14.24-39.9	24.62	15.33-41.43	13	0.84-32.46	0.3691	0.461375
Bacilli	0.98	0.51-8.08	1.72	0.25-9.08	1.78	0.67-14.2	4.1	1.12-31.68	0.1191	0.397
Clostridia	45.16	16.93-59.11	50.55	44.28-57.19	43.9	14.79-55.89	55.58	29.54-85.45	0.7892	0.7892
Erysipelotrichi	0.9	0-3.05	1.66	0.45-2.98	1.31	0.03-4.11	1.75	0.88-4.02	0.2261	0.428286
Fusobacteriia	14.98	8.68-36.44	10.95	0.67-22.15	11.47	4.27-38.48	9.6	1.01-14.72	0.0979	0.397
Betaproteobacteria	3.29	0.99-5.62	1.92	0.76-3.63	1.59	0.72-4.27	0.58	0.13-3.36	0.0576	0.397
Epsilonproteobacteria	1.95	0.1-5	1.09	0.1-7.12	0.62	0-7.7	0.09	0-3.52	0.2174	0.428286
Gammaproteobacteria	0.64	0.08-14.55	1.49	0.49-7.26	1.92	0.62-14.19	1.62	0.48-18.99	0.2898	0.428286

ORDER

Bacterial group	Day 0		Day 7		Day 21		Day 42		Day 0 vs Day 7 vs Day 21 vs Day 42	
	Median	Range	Median	Range	Median	Range	Median	Range	P value	Q value

Bifidobacteriales	0	0-0.15	0	0-0.21	0	0-0.4	0.01	0-4.96	0.4883	0.7425
Coriobacteriales	1	0.19-2.75	1.18	0.8-1.58	1.27	0.1-3.09	1.28	0-4.11	0.5828	0.777067
Bacteroidales	23.14	13.71-38.3	23.73	14.24-39.9	24.62	15.33-41.43	13	0.84-32.46	0.3691	0.7382
Lactobacillales	0.77	0.33-3.98	0.9	0.21-8.51	0.77	0.39-14.16	1	0.6-30.71	0.9852	0.9852
Turicibacterales	0.38	0.03-4.1	0.62	0.03-1.71	0.45	0-2.28	1.82	0.07-5.1	0.2561	0.61464
Clostridiales	45.16	16.93-59.11	50.55	44.28-57.19	43.9	14.79-55.89	55.58	29.54-85.45	0.7892	0.914618
Erysipelotrichales	0.9	0-3.05	1.66	0.45-2.98	1.31	0.03-4.11	1.75	0.88-4.02	0.2261	0.61464
Fusobacteriales	14.98	8.68-36.44	10.95	0.67-22.15	11.47	4.27-38.48	9.6	1.01-14.72	0.0979	0.5874
Burkholderiales	3.29	0.99-5.62	1.92	0.76-3.63	1.59	0.72-4.27	0.58	0.13-3.36	0.0576	0.5874
Campylobacterales	1.95	0.1-5	1.09	0.1-7.12	0.62	0-7.7	0.09	0-3.52	0.2174	0.61464
Aeromonadales	0.05	0-14.46	0.13	0-6.76	0.05	0-13.28	0.07	0.02-18.58	0.8384	0.914618
Enterobacteriales	0.51	0.08-0.79	0.59	0.4-6.86	1.26	0.38-4.49	1.09	0.38-10.56	0.495	0.7425

FAMILY

Bacterial group	Day 0		Day 7		Day 21		Day 42		Day 0 vs Day 7 vs Day 21 vs Day 42	
	Median	Range	Median	Range	Median	Range	Median	Range	P value	Q value
Bifidobacteriaceae	0	0-0.15	0	0-0.21	0	0-0.4	0.01	0-4.96	0.4883	0.784929
Coriobacteriaceae	1	0.19-2.75	1.18	0.8-1.58	1.27	0.1-3.09	1.28	0-4.11	0.5828	0.854773
Bacteroidaceae	18.9	10.78-38.23	20.67	10.39-39.73	19.24	12.33-41.23	12.58	0.63-25.21	0.1191	0.52404
Porphyromonadaceae	0.02	0-2.62	0	0-2.39	0.01	0-1.34	0	0-1.35	0.2375	0.626022
Prevotellaceae	1.03	0-11.54	2.58	0-10.91	2.49	0-9.85	0.15	0-8.03	0.9637	0.9852
[Paraprevotellaceae]	0.23	0-3.63	0.5	0-4	0.96	0-3.7	0.07	0-4.36	0.761	0.984824
Enterococcaceae	0.21	0-0.69	0.24	0.21-8.14	0.23	0.16-0.51	0.27	0.11-14.27	0.9189	0.9852
Streptococcaceae	0.48	0.29-3.06	0.49	0-6.51	0.44	0.23-12.68	0.59	0.29-21.7	0.7222	0.984824
Turicibacteraceae	0.38	0.03-4.1	0.62	0.03-1.71	0.45	0-2.28	1.82	0.07-5.1	0.2561	0.626022
o__Clostridiales;f__	1.09[a]	0.1-2.94	1.77[a,b]	0.21-3.41	0.5[a,b]	0-3.03	0.41[b]	0-1.09	0.0059	0.1298
Clostridiaceae	7.36	3.95-19.29	9.59	3.65-14.8	8.78	3.75-16.53	8.55	4.12-43.89	0.8614	0.9852
Lachnospiraceae	19.39	10.13-32.62	25.34	8.74-33.42	19.97	7.14-24.64	19.71	13.05-47.17	0.2898	0.63756
Peptostreptococcaceae	0.11	0-0.68	0.11	0-0.58	0.63	0.12-3.24	0.53	0-8.27	0.2123	0.626022
Ruminococcaceae	4.18	0.23-10.02	4.99	1.11-10.2	2.1	0.14-9.02	3.92	0.48-7.1	0.3691	0.7382
Veillonellaceae	4.18	1.19-30.56	8.4	0.23-23.21	5.63	0.91-26.01	7.46	0.58-29.23	0.9852	0.9852
Erysipelotrichaceae	0.9	0-3.05	1.66	0.45-2.98	1.31	0.03-4.11	1.75	0.88-4.02	0.2261	0.626022

Fusobacteriaceae	14.98	8.68-36.44	10.95	0.67-22.15	11.47	4.27-38.48	9.6	1.01-14.72	0.0979	0.52404
Alcaligenaceae	3.29	0.99-5.62	1.92	0.76-3.63	1.59	0.72-4.27	0.58	0.13-3.36	0.0576	0.52404
Campylobacteraceae	0.47	0-2.24	0.05	0-1.56	0	0-0.71	0	0-0.71	0.1001	0.52404
Helicobacteraceae	0.52	0.04-4.87	0.15	0-7.12	0.59	0-7.08	0.09	0-3.52	0.4995	0.784929
Succinivibrionaceae	0.05	0-14.46	0.13	0-6.76	0.05	0-13.28	0.07	0.02-18.58	0.8384	0.9852
Enterobacteriaceae	0.51	0.08-0.79	0.59	0.4-6.86	1.26	0.38-4.49	1.09	0.38-10.56	0.495	0.784929

GENUS

Bacterial group	Day 0		Day 7		Day 21		Day 42		Day 0 vs Day 7 vs Day 21 vs Day 42	
	Median	Range	Median	Range	Median	Range	Median	Range	P value	Q value
Bifidobacterium	0	0-0.15	0	0-0.21	0	0-0.4	0.01	0-4.96	0.4883	0.787673
Collinsella	0.99	0.19-2.22	1.18	0.55-1.41	1.27	0.1-2.49	1.28	0-4.11	0.6823	0.913555
Slackia	0	0-0.53	0	0-0.2	0.01	0-0.6	0	0-0.02	0.416	0.776438
Bacteroides	18.9	10.78-38.23	20.66	10.39-39.73	19.21	12.33-41.23	12.58	0.63-25.21	0.1191	0.50102
Parabacteroides	0.02	0-2.62	0	0-2.39	0.01	0-1.34	0	0-1.35	0.2375	0.608594
Prevotella	1.03	0-11.54	2.58	0-10.91	2.49	0-9.85	0.15	0-8.03	0.9637	0.9852
[Prevotella]	0.23	0-3.62	0.5	0-4	0.96	0-3.7	0.07	0-4.35	0.7353	0.913555
Enterococcus	0.21	0-0.69	0.24	0.21-8.14	0.23	0.16-0.51	0.27	0.11-14.27	0.9189	0.9852
Streptococcus	0.48	0.29-3.06	0.49	0-6.51	0.44	0.23-12.68	0.59	0.29-21.67	0.7222	0.913555
Turicibacter	0.38	0.03-4.1	0.62	0.03-1.71	0.45	0-2.28	1.82	0.07-5.1	0.2561	0.617653
o__Clostridiales;f__;g__	1.09[a]	0.1-2.94	1.77[a,b]	0.21-3.41	0.5[a,b]	0-3.03	0.41[b]	0-1.09	0.0059	0.2419
f__Clostridiaceae;__	6.75	3.67-18.77	7.54	3.65-14.22	7.59	1.94-15.23	7.9	3.71-43.19	0.8614	0.9852
f__Clostridiaceae;g__	0.38	0-0.89	0.42	0-1.89	0.7	0-1.8	0.42	0.19-1.34	0.7006	0.913555
Clostridium	0	0-0.19	0.16	0-1.18	0.19	0-2.03	0	0-4.56	0.5573	0.826004
f__Lachnospiraceae;__	4.81	1.76-10.04	7.01	1.51-20.03	4.29	2.85-8.39	4.68	1.55-31.71	0.8964	0.9852
f__Lachnospiraceae;g__	3.54	1.13-5.36	3.13	0.81-5.05	1.92	0.96-9.46	1.37	0.54-4.99	0.0803	0.50102
Blautia	4.67	2.16-13.73	6.92	4.69-10.77	7.85	0.99-13.41	9.6	3.15-18.4	0.1993	0.586027
Coprococcus	0	0-0.35	0.1	0-0.56	0	0-0.4	0	0-0.17	0.0803	0.50102
Dorea	0.9	0-2.86	1.23	0-2.93	0.28	0-2.37	0.16	0-3.47	0.4281	0.776438
Roseburia	0.05	0-0.5	0.04	0-1.05	0	0-0.72	0	0-1.66	0.9469	0.9852
[Ruminococcus]	2.95	0.34-7.17	2.31	1.15-5.35	2.65	0.22-5.17	2.91	0.33-4.97	0.7892	0.951682
f__Peptostreptococcaceae;g__	0.11	0-0.4	0.05	0-0.58	0.55	0-3.24	0.53	0-5.15	0.2085	0.586027

f__Ruminococcaceae;g__	0.74	0.08-3.52	0.75	0.1-2.38	0.56	0-3.54	0.81	0-4.22	0.204	0.586027
Faecalibacterium	3.18	0.15-5.65	3.28	0.71-9.05	1.66	0.14-6.02	1.51	0.28-5.73	0.4402	0.776438
Oscillospira	0.02	0-0.79	0.04	0-0.25	0	0-0.59	0	0-0.15	0.4318	0.776438
Ruminococcus	0.06	0-0.19	0.06	0-0.39	0.09	0-0.65	0.05	0-0.16	0.1077	0.50102
Megamonas	2.88	0.52-29.31	7.29	0.23-21.41	4.93	0.52-25.91	6.51	0.38-29.17	0.9852	0.9852
Phascolarctobacterium	0.54	0-1.35	1.11	0-2.29	0.46	0-1.41	0.1	0-1.86	0.5641	0.826004
f__Erysipelotrichaceae;g__	0.13	0-1.44	0.36	0-2.02	0.11	0.02-2.3	0.35	0-0.97	0.6182	0.874007
Allobaculum	0.03	0-0.18	0.06	0-0.28	0.05	0-0.25	0.01	0-0.6	0.4394	0.776438
Catenibacterium	0.19	0-2.63	0.06	0-2.26	0.36	0-0.99	0.27	0-2.61	0.8866	0.9852
[Eubacterium]	0.17[a]	0-0.67	0.28[a,b]	0.06-0.96	0.19[a,b]	0-1.7	0.64[b]	0.23-1.51	0.0273	0.50102
Cetobacterium	0.04	0-0.14	0	0-0.17	0	0-0.19	0	0-0.07	0.4367	0.776438
Fusobacterium	14.89	8.68-36.3	10.9	0.67-21.98	11.47	4.27-38.29	9.6	1.01-14.72	0.0979	0.50102
Sutterella	3.29	0.99-5.62	1.92	0.76-3.63	1.59	0.72-4.27	0.58	0.13-3.36	0.0576	0.50102
Campylobacter	0.47	0-2.24	0.05	0-1.56	0	0-0.71	0	0-0.71	0.1001	0.50102
Helicobacter	0.52	0.04-4.87	0.15	0-7.12	0.59	0-7.08	0.09	0-3.52	0.4995	0.787673
f__Succinivibrionaceae;__	0.02	0-0.47	0	0-0.76	0	0-1.55	0	0-3.88	0.4545	0.776438
f__Succinivibrionaceae;g__	0.02	0-13.93	0.07	0-6.7	0.05	0-11.63	0.06	0-14.68	0.2144	0.586027
Succinivibrio	0	0-0.31	0.03	0-0.11	0.01	0-0.16	0	0-0.03	0.1222	0.50102
f__Enterobacteriaceae;g__	0.51	0.08-0.79	0.59	0.4-6.86	1.18	0.38-4.49	1.09	0.38-10.56	0.1852	0.586027

SPECIES

Bacterial group	Day 0		Day 7		Day 21		Day 42		Day 0 vs Day 7 vs Day 21 vs Day 42	
	Median	Range	Median	Range	Median	Range	Median	Range	P value	Q value
g__Bifidobacterium;s__	0	0-0.15	0	0-0.18	0	0-0.4	0.01	0-4.96	0.2156	0.598889
Collinsella stercoris	0.99	0.19-2.09	1.11	0.55-1.41	1.27	0.1-2.26	1.28	0-4.11	0.9297	0.983367
g__Slackia;s__	0	0-0.53	0	0-0.2	0.01	0-0.6	0	0-0.02	0.416	0.783621
g__Bacteroides;__	5.76	2.75-16.62	5.36	3.2-10.49	6.21	3.58-18.91	5.08	0-8.2	0.5828	0.88303
g__Bacteroides;s__	8.55	1.86-18.36	6.9	3.21-20.44	10.34	1.84-28.55	3.24	0.23-12.98	0.6481	0.900139
Bacteroides fragilis	0[a]	0-0.1	0.05[a]	0-0.42	0.13[a]	0-0.29	0[a]	0-0.12	0.0186	0.465
Bacteroides plebeius	3	0.24-10.94	3.8	0.18-10.16	3.45	0.2-6.7	0.75	0.1-7.89	0.1357	0.565417
Bacteroides uniformis	0[a]	0-1.22	0.07[a]	0-15.84	0[a]	0-5.39	0[a]	0-0.46	0.0471	0.5385

g__Parabacteroides;s__	0.01	0-2.62	0	0-2.02	0.01	0-1.34	0	0-1.24	0.5118	0.825484
Prevotella copri	1.03	0-11.54	2.58	0-10.91	2.49	0-9.85	0.15	0-8.03	0.9637	0.983367
g__[Prevotella];s__	0.23	0-3.62	0.5	0-4	0.96	0-3.7	0.07	0-4.35	0.7353	0.942692
g__Enterococcus;s__	0.21	0-0.69	0.24	0.21-8.14	0.23	0.16-0.51	0.27	0.11-14.27	0.9189	0.983367
g__Streptococcus;s__	0.48	0.29-3.06	0.49	0-6.51	0.44	0.23-12.68	0.59	0.29-21.67	0.7222	0.942692
g__Turicibacter;s__	0.38	0.03-4.1	0.62	0.03-1.71	0.45	0-2.28	1.82	0.07-5.1	0.2561	0.673947
o__Clostridiales;f__;g__;s__	1.09[a]	0.1-2.94	1.77[a,b]	0.21-3.41	0.5[a,b]	0-3.03	0.41[b]	0-1.09	0.0059	0.295
f__Clostridiaceae;__;__	6.75	3.67-18.77	7.54	3.65-14.22	7.59	1.94-15.23	7.9	3.71-43.19	0.8614	0.983367
f__Clostridiaceae;g__;s__	0.38	0-0.89	0.42	0-1.89	0.7	0-1.8	0.42	0.19-1.34	0.7006	0.942692
g__Clostridium;__	0	0-0.16	0	0-0.92	0.08	0-0.24	0	0-4.56	0.7885	0.983367
Clostridium perfringens	0	0-0.19	0	0-0.41	0.02	0-2.03	0	0-0.34	0.302	0.686364
f__Lachnospiraceae;__;__	4.81	1.76-10.04	7.01	1.51-20.03	4.29	2.85-8.39	4.68	1.55-31.71	0.8964	0.983367
f__Lachnospiraceae;g__;s__	3.54	1.13-5.36	3.13	0.81-5.05	1.92	0.96-9.46	1.37	0.54-4.99	0.0803	0.5385
g__Blautia;s__	2.33	0.44-9.14	3.43	2.12-4.75	2.84	0-9.04	3.28	1.33-6.32	0.2898	0.686364
Blautia producta	2.89	1.72-5.86	3.96	2.57-7.14	3.86	0.99-6.82	5.1	1.82-15.56	0.2725	0.68125
g__Coprococcus;s__	0	0-0.35	0.1	0-0.56	0	0-0.4	0	0-0.17	0.0803	0.5385
g__Dorea;s__	0.9	0-2.86	1.23	0-2.93	0.28	0-2.37	0.16	0-3.47	0.4281	0.783621
g__Roseburia;s__	0.05	0-0.5	0.04	0-1.05	0	0-0.72	0	0-1.66	0.9469	0.983367
g__[Ruminococcus];__	0.16	0-3.83	0.08	0-3.44	0.02	0-0.89	0.03	0-1.14	0.8179	0.983367
g__[Ruminococcus];s__	0.53	0-1.42	0.55	0.2-1.77	0.39	0-1.61	0.31	0-2.41	0.635	0.900139
[Ruminococcus] gnavus	1.23	0-5.33	1.48	0.79-3.43	1.37	0-3.71	2.04	0-3.1	0.9297	0.983367
f__Peptostreptococcaceae;g__;s__	0.11	0-0.4	0.05	0-0.58	0.55	0-3.24	0.53	0-5.15	0.2085	0.598889
f__Ruminococcaceae;g__;s__	0.74	0.08-3.52	0.75	0.1-2.38	0.56	0-3.54	0.81	0-4.22	0.204	0.598889
Faecalibacterium prausnitzii	3.18	0.15-5.65	3.28	0.71-9.05	1.66	0.14-6.02	1.51	0.28-5.73	0.4402	0.783621
g__Oscillospira;s__	0.02	0-0.79	0.04	0-0.25	0	0-0.59	0	0-0.15	0.4318	0.783621
g__Ruminococcus;s__	0.06	0-0.19	0.06	0-0.39	0.09	0-0.65	0.05	0-0.16	0.1077	0.5385
g__Megamonas;s__	2.88	0.52-29.31	7.29	0.23-21.41	4.93	0.52-25.91	6.51	0.38-29.17	0.9852	0.9852
g__Phascolarctobacterium;s__	0.54	0-1.35	1.11	0-2.29	0.46	0-1.41	0.1	0-1.86	0.5641	0.881406
f__Erysipelotrichaceae;g__;s__	0.13	0-1.44	0.36	0-2.02	0.11	0.02-2.3	0.35	0-0.97	0.6182	0.900139
g__Allobaculum;s__	0.03	0-0.18	0.06	0-0.28	0.05	0-0.25	0.01	0-0.6	0.4394	0.783621
g__Catenibacterium;s__	0.19	0-2.63	0.06	0-2.26	0.36	0-0.99	0.27	0-2.61	0.8866	0.983367
[Eubacterium] biforme	0.13[a]	0-0.67	0.18[a]	0-0.96	0.16[a]	0-1.67	0.5[a]	0-1.09	0.0469	0.5385
[Eubacterium] dolichum	0.01	0-0.1	0.07	0-0.15	0.04	0-0.06	0.04	0-0.98	0.2014	0.598889
Cetobacterium somerae	0.04	0-0.14	0	0-0.17	0	0-0.19	0	0-0.07	0.4367	0.783621

g__Fusobacterium;s__	14.89	8.68-36.3	10.9	0.67-21.98	11.47	4.27-38.29	9.6	1.01-14.72	0.0979	0.5385
g__Sutterella;s__	3.29	0.99-5.62	1.92	0.76-3.63	1.59	0.72-4.27	0.58	0.13-3.36	0.0576	0.5385
g__Campylobacter;s__	0.47	0-2.24	0.05	0-1.56	0	0-0.71	0	0-0.71	0.1001	0.5385
g__Helicobacter;s__	0.52	0.04-4.24	0.15	0-7.12	0.59	0-6.17	0.09	0-3.52	0.4995	0.825484
f__Succinivibrionaceae;__;__	0.02	0-0.47	0	0-0.76	0	0-1.55	0	0-3.88	0.4545	0.783621
f__Succinivibrionaceae;g__;s__	0.02	0-13.93	0.07	0-6.7	0.05	0-11.63	0.06	0-14.68	0.2144	0.598889
g__Succinivibrio;s__	0	0-0.31	0.03	0-0.11	0.01	0-0.16	0	0-0.03	0.1222	0.555455
f__Enterobacteriaceae;g__;s__	0.51	0.08-0.79	0.59	0.4-6.86	1.18	0.38-4.49	1.09	0.38-10.56	0.1852	0.598889
