# Supplementary material for: Effects of metronidazole on the fecal microbiome and metabolome in healthy dogs
Source: J Vet Intern Med. 2020 Aug 28;34(5):1853–66. doi: 10.1111/jvim.15871 (PMC7517498; doi:10.1111/jvim.15871)
Supplement: Supplementary file 2 — Supplementary Data S2. List of relevant bacterial taxa detected in fecal samples from group 2 during the hydrolyzed protein diet trial, separated by taxonomic level, with median and range for each time point. Time points were compared with Friedman test, and adjusted for multiple comparison using Benjamini and Hochberg's False Discovery Rate, and p‐ and q‐values are presented. Post hoc Dunn's multiple comparison test was used to determine the bacterial taxa that were different between the time‐points, and significant differences are indicated by different superscript letters. [file JVIM-34-1853-s002.pdf]

PHYLUM

Bacterial group	Day 0		Day 7		Day 21		Day 42		Day 0 vs Day 7 vs Day 21 vs Day 42	
	Median	Range	Median	Range	Median	Range	Median	Range	P value	Q value
Actinobacteria	1.21	0.58-2.38	2.08	0.46-3.94	1.43	0.19-5.43	1.87	0.38-4	0.3916	0.4895
Bacteroidetes	30.28	7.69-44.94	17.63	1.01-43.57	20.33	0.4-47.76	17.62	0.89-32.94	0.1116	0.36175
Firmicutes	48.19	20.91-80.31	62.77	30.55-92.35	63.35	25.98-83.58	65.93	42.47-75.26	0.3273	0.4895
Fusobacteria	13.57	5.21-32.62	11.39	2.26-30.43	13.09	2.15-26.04	13.06	3.24-29.49	0.5222	0.5222
Proteobacteria	5.28	1.45-10.99	5.35	1.24-7.76	7.8	1.55-13.68	4.94	0.98-16.75	0.1447	0.36175

CLASS

Bacterial group	Day 0		Day 7		Day 21		Day 42		Day 0 vs Day 7 vs Day 21 vs Day 42	
	Median	Range	Median	Range	Median	Range	Median	Range	P value	Q value
Actinobacteria	0.08	0-0.19	0.14	0-0.23	0.07	0-0.58	0.06	0-0.38	0.7969	0.885444
Coriobacteriia	1.2	0.44-2.38	1.96	0.46-3.8	1.35	0.19-4.85	1.73	0-4	0.5222	0.885444
Bacteroidia	30.28	7.69-44.94	17.63	1.01-43.57	20.33	0.4-47.76	17.62	0.89-32.94	0.1116	0.558
Bacilli	0.96	0.71-22.97	1.08	0.6-4.06	1.55	0.58-13.12	1.86	0.6-24.12	0.7892	0.885444
Clostridia	40.83	20.13-71.64	60.93	27.96-84.7	54.7	24.77-78.37	54.35	40.82-67.27	0.2898	0.885444
Erysipelotrichi	1.03	0-7.92	1.2	0.35-6.88	0.34	0-3.19	0.99	0.03-7.05	0.5828	0.885444
Fusobacteriia	13.57	5.21-32.62	11.39	2.26-30.43	13.09	2.15-26.04	13.06	3.24-29.49	0.5222	0.885444
Betaproteobacteria	1.9	0.18-5.18	0.97	0.18-6.08	0.81	0.28-7.44	0.41	0.28-1.96	0.0841	0.558
Epsilonproteobacteria	0.14	0-3.58	0.15	0-0.86	0.29	0.06-1.36	0.25	0-2.24	0.9203	0.9203
Gammaproteobacteria	1.89	0.61-5.74	1.3	0.77-4.49	3.97	0.66-13.25	2.16	0.45-16.17	0.7892	0.885444

ORDER

Bacterial group	Day 0		Day 7		Day 21		Day 42		Day 0 vs Day 7 vs Day 21 vs Day 42	
	Median	Range	Median	Range	Median	Range	Median	Range	P value	Q value

Bifidobacteriales	0.08	0-0.19	0.07	0-0.21	0	0-0.58	0.06	0-0.38	0.8423	0.9203
Coriobacteriales	1.2	0.44-2.38	1.96	0.46-3.8	1.35	0.19-4.85	1.73	0-4	0.5222	0.9203
Bacteroidales	30.28	7.69-44.94	17.63	1.01-43.57	20.33	0.4-47.76	17.62	0.89-32.94	0.1116	0.6696
Lactobacillales	0.76	0.62-11.17	0.94	0.6-1.65	0.82	0.58-12.93	1.4	0.58-23.89	0.7173	0.9203
Turicibacterales	0.19	0-18.99	0.1	0-2.55	0.18	0-5.44	0.18	0-6.03	0.5566	0.9203
Clostridiales	40.83	20.13-71.64	60.93	27.96-84.7	54.7	24.77-78.37	54.35	40.82-67.27	0.2898	0.9203
Erysipelotrichales	1.03	0-7.92	1.2	0.35-6.88	0.34	0-3.19	0.99	0.03-7.05	0.5828	0.9203
Fusobacteriales	13.57	5.21-32.62	11.39	2.26-30.43	13.09	2.15-26.04	13.06	3.24-29.49	0.5222	0.9203
Burkholderiales	1.9	0.18-5.18	0.97	0.18-6.08	0.81	0.28-7.44	0.41	0.28-1.96	0.0841	0.6696
Campylobacterales	0.14	0-3.58	0.15	0-0.86	0.29	0.06-1.36	0.25	0-2.24	0.9203	0.9203
Aeromonadales	0.09	0-1.77	0.06	0-3.61	0.07	0-6.56	0.07	0-3.13	0.8887	0.9203
Enterobacteriales	0.75	0.51-5.62	0.9	0.39-4.44	1.44	0.49-13.25	0.88	0.12-16	0.7892	0.9203

FAMILY

Bacterial group	Day 0		Day 7		Day 21		Day 42		Day 0 vs Day 7 vs Day 21 vs Day 42	
	Median	Range	Median	Range	Median	Range	Median	Range	P value	Q value
Bifidobacteriaceae	0.08	0-0.19	0.07	0-0.21	0	0-0.58	0.06	0-0.38	0.8423	0.922519
Coriobacteriaceae	1.2	0.44-2.38	1.96	0.46-3.8	1.35	0.19-4.85	1.73	0-4	0.5222	0.788494
Bacteroidaceae	27.74	5.3-31.35	10.33	1.01-26.21	20.05	0.32-42.75	14.66	0.79-28.87	0.2561	0.736288
Prevotellaceae	0.61	0-9.45	0.12	0-17.75	0.11	0.07-0.45	0.12	0-4.01	0.1975	0.648929
[Paraprevotellaceae]	1.86	0-8.66	0.45	0-17.2	0.14	0-5.88	0.2	0-3.82	0.3189	0.7567
Enterococcaceae	0.25	0.2-10.11	0.27	0.22-0.37	0.26	0.2-1.99	0.29	0.18-1.17	0.7793	0.914365
Lactobacillaceae	0.1	0-0.41	0.18	0-0.34	0.07	0-10.16	0.15	0-21.28	0.5566	0.788494
Streptococcaceae	0.5	0.34-0.6	0.47	0.37-1.16	0.47	0.34-0.77	0.58	0.37-1.84	0.5222	0.788494
Turicibacteraceae	0.19	0-18.99	0.1	0-2.55	0.18	0-5.44	0.18	0-6.03	0.5566	0.788494
o__Clostridiales;f__	0.2	0-3.17	0.28	0-1.57	0.2	0-1.76	0.28	0-1.42	0.9651	0.9651
Clostridiaceae	9.11	1.98-24.95	13.93	4.49-36.01	17.93	2.88-62.04	17.91	4.88-41.14	0.1755	0.648929
Lachnospiraceae	14.94[a]	2.5-49.24	32.9[a]	8.8-43.95	20.26[a]	7.24-29.74	23.93[a]	15.64-39.78	0.0411	0.57845
Peptococcaceae	0	0-0	0	0-0.05	0.01	0-0.19	0	0-0.88	0.1299	0.648929
Peptostreptococcaceae	0.05	0-0.36	0	0-1.34	0.07	0-9	0	0-11.15	0.329	0.7567
Ruminococcaceae	1.7	0.18-11.24	2.64	0.59-11.06	2.45	0.12-7.41	1.94	0.27-11.26	0.3916	0.788494
Veillonellaceae	5.53	0.85-15.47	3.5	1.56-23.95	1.54	0.42-15.06	2.97	0.6-18.93	0.0503	0.57845

Erysipelotrichaceae	1.03	0-7.92	1.2	0.35-6.88	0.34	0-3.19	0.99	0.03-7.05	0.5828	0.788494
Fusobacteriaceae	13.57	5.21-32.62	11.39	2.26-30.43	13.09	2.15-26.04	13.06	3.24-29.49	0.5222	0.788494
Alcaligenaceae	1.9	0.18-5.18	0.97	0.18-6.08	0.81	0.28-7.44	0.41	0.28-1.96	0.0841	0.644767
Campylobacteraceae	0.01	0-0.64	0	0-0	0	0-0.11	0	0-1.46	0.1439	0.648929
Helicobacteraceae	0.14	0-3.39	0.15	0-0.86	0.29	0.06-1.36	0.25	0-1.4	0.7951	0.914365
Succinivibrionaceae	0.09	0-1.77	0.06	0-3.61	0.07	0-6.56	0.07	0-3.13	0.8887	0.929095
Enterobacteriaceae	0.75	0.51-5.62	0.9	0.39-4.44	1.44	0.49-13.25	0.88	0.12-16	0.7892	0.914365

GENUS

Bacterial group	Day 0		Day 7		Day 21		Day 42		Day 0 vs Day 7 vs Day 21 vs Day 42	
	Median	Range	Median	Range	Median	Range	Median	Range	P value	Q value
Bifidobacterium	0.08	0-0.19	0.07	0-0.21	0	0-0.58	0.06	0-0.38	0.8423	0.912071
Collinsella	1.11	0.44-1.92	1.89	0.46-3.49	1.2	0.19-4.85	1.7	0-4	0.4153	0.801504
Slackia	0.01	0-0.46	0	0-0.34	0	0-0.31	0.02	0-0.17	0.4531	0.801504
Bacteroides	27.74	5.3-31.35	10.33	0.99-26.21	20.04	0.32-42.75	14.66	0.79-28.87	0.2561	0.801504
Prevotella	0.61	0-9.45	0.12	0-17.75	0.11	0.07-0.45	0.12	0-4.01	0.1975	0.79
[Prevotella]	1.86	0-8.66	0.45	0-17.2	0.14	0-5.88	0.2	0-3.82	0.3189	0.801504
Enterococcus	0.25	0.2-10.11	0.27	0.22-0.37	0.26	0.2-1.99	0.29	0.18-1.17	0.7793	0.912071
Lactobacillus	0.1	0-0.41	0.18	0-0.34	0.07	0-10.16	0.15	0-21.28	0.5566	0.801504
Streptococcus	0.5	0.34-0.55	0.47	0.37-1.16	0.46	0.34-0.77	0.55	0.37-1.84	0.4153	0.801504
Turicibacter	0.19	0-18.99	0.1	0-2.55	0.18	0-5.44	0.18	0-6.03	0.5566	0.801504
o__Clostridiales;f__;g__	0.2	0-3.17	0.28	0-1.57	0.2	0-1.76	0.28	0-1.42	0.9651	0.9651
f__Clostridiaceae;__	7.8	1.98-22.59	13.62	4.34-34.39	16.32	2.71-36.4	15.05	4.65-30.94	0.1544	0.79
f__Clostridiaceae;g__	0.22	0-0.76	0.39	0.06-1.62	0.96	0-24.08	0.35	0-9.7	0.1628	0.79
Clostridium	0	0-1.53	0.14	0-2.5	0.25	0-2.87	0.12	0-5	0.6766	0.877243
f__Lachnospiraceae;__	4.53	0.36-32.43	8.9	1.8-20.18	7.25	0.96-18.15	6.58	3.54-14.32	0.6823	0.877243
f__Lachnospiraceae;g__	1.44	0.42-6.46	2.05	0.51-3.19	1.32	0-2.93	1.51	0.18-2.05	0.4663	0.801504
Blautia	5.92	0.42-11.12	7.14	1.78-14.71	4.8	1.68-11.43	6.03	2.28-9.89	0.3273	0.801504
Dorea	0.12	0-1.33	0	0-0.38	0.06	0-0.75	0	0-1.01	0.9228	0.949166
[Ruminococcus]	3.18	0.88-7.3	8.24	1.77-14.84	3.62	1.96-8.34	8.53	2.81-15.7	0.0703	0.79
Peptococcus	0	0-0	0	0-0.05	0.01	0-0.19	0	0-0.88	0.1299	0.79
f__Peptostreptococcaceae;g__	0.05	0-0.36	0	0-1.34	0.07	0-8.99	0	0-11.15	0.329	0.801504

f__Ruminococcaceae;g__	0.6	0.06-1.55	0.91	0-1.93	0.62	0-1.64	0.44	0-1.28	0.2561	0.801504
Faecalibacterium	1.09	0.12-9.69	1.75	0.33-9.31	1.71	0-5.93	1.05	0.14-9.44	0.8614	0.912071
Ruminococcus	0	0-0.18	0.02	0-0.37	0	0-0.14	0.01	0-0.1	0.4668	0.801504
Megamonas	5.26[a]	0.58-15.36	3.39[a,b]	1.12-20.22	1.22[b]	0.32-13.44	1.68[a,b]	0.54-18.93	0.0239	0.79
Phascolarctobacterium	0.43	0-1.59	0.35	0.05-3.58	0.24	0-1.63	0.18	0-1.23	0.3578	0.801504
f__Erysipelotrichaceae;g__	0.24	0-3.93	0.29	0-0.87	0.05	0-2.23	0.1	0-5.4	0.8145	0.912071
Allobaculum	0.1	0-0.58	0.05	0-4.28	0.05	0-0.3	0.17	0-1.29	0.5363	0.801504
Catenibacterium	0.31	0-1.57	0.22	0-1.19	0.05	0-0.19	0.12	0-1.35	0.5931	0.821215
[Eubacterium]	0.22	0-2.33	0.52	0.1-1.35	0.17	0-0.66	0.29	0.03-1.24	0.1975	0.79
Fusobacterium	13.51	5.21-32.62	11.39	2.26-30.43	13.09	2.15-26.04	13.06	3.24-29.49	0.5222	0.801504
Sutterella	1.9	0.18-5.18	0.97	0.18-6.08	0.81	0.28-7.44	0.41	0.28-1.96	0.0841	0.79
Campylobacter	0.01	0-0.64	0	0-0	0	0-0.11	0	0-1.46	0.1439	0.79
Helicobacter	0.14	0-3.39	0.15	0-0.86	0.29	0.06-1.36	0.25	0-1.4	0.7951	0.912071
f__Succinivibrionaceae;g__	0	0-1.54	0.03	0-3.09	0.04	0-6.22	0.02	0-0.79	0.4914	0.801504
f__Enterobacteriaceae;g__	0.75	0.51-5.62	0.9	0.39-4.44	1.44	0.49-13.25	0.88	0.12-16	0.7892	0.912071

SPECIES

Bacterial group	Day 0		Day 7		Day 21		Day 42		Day 0 vs Day 7 vs Day 21 vs Day 42	
	Median	Range	Median	Range	Median	Range	Median	Range	P value	Q value
g__Bifidobacterium;s__	0.01	0-0.19	0	0-0.21	0	0-0.58	0.06	0-0.38	0.3194	0.732653
Collinsella stercoris	1.11	0.44-1.92	1.89	0.46-3.49	1.2	0.19-4.85	1.7	0-4	0.4153	0.732653
g__Slackia;s__	0.01	0-0.46	0	0-0.34	0	0-0.31	0.02	0-0.17	0.4531	0.732653
g__Bacteroides;__	9.41	2.34-23.44	4.65	0.51-13.72	9.74	0.23-28.67	6.49	0.2-18.78	0.5828	0.732653
g__Bacteroides;s__	7.11	1.75-16.5	3.22	0.27-18.35	4.58	0.08-19.6	3.28	0.4-14.01	0.1447	0.68376
Bacteroides plebeius	2.42	0.16-10.49	1.25	0-10.34	1.98	0-8.93	0.69	0-7.21	0.5856	0.732653
Prevotella copri	0.61	0-9.45	0.12	0-17.75	0.11	0.07-0.45	0.12	0-4.01	0.1975	0.732653
g__[Prevotella];s__	1.86	0-8.66	0.45	0-17.2	0.14	0-5.88	0.2	0-3.82	0.3189	0.732653
g__Enterococcus;s__	0.25	0.2-10.11	0.27	0.22-0.37	0.26	0.2-1.99	0.29	0.18-1.17	0.7793	0.895849
g__Lactobacillus;s__	0.1	0-0.41	0.18	0-0.34	0.07	0-10.16	0.15	0-21.28	0.5566	0.732653
g__Streptococcus;s__	0.5	0.34-0.55	0.47	0.37-1.16	0.46	0.34-0.77	0.55	0.37-1.84	0.4153	0.732653
g__Turicibacter;s__	0.19	0-18.99	0.1	0-2.55	0.18	0-5.44	0.18	0-6.03	0.5566	0.732653
o__Clostridiales;f__;g__;s__	0.2	0-3.17	0.28	0-1.57	0.2	0-1.76	0.28	0-1.42	0.9651	0.9651

f__Clostridiaceae;__;__	7.8	1.98-22.59	13.62	4.34-34.39	16.32	2.71-36.4	15.05	4.65-30.94	0.1544	0.68376
f__Clostridiaceae;g__;s__	0.22	0-0.76	0.39	0.06-1.62	0.96	0-24.08	0.35	0-9.7	0.1628	0.68376
Clostridium perfringens	0	0-0.92	0	0-2.5	0	0-1.29	0.06	0-3.15	0.4402	0.732653
f__Lachnospiraceae;__;__	4.53	0.36-32.43	8.9	1.8-20.18	7.25	0.96-18.15	6.58	3.54-14.32	0.6823	0.81876
f__Lachnospiraceae;g__;s__	1.44	0.42-6.46	2.05	0.51-3.19	1.32	0-2.93	1.51	0.18-2.05	0.4663	0.732653
g__Blautia;s__	2.04[a,b]	0-6.07	2.05[a]	0.4-8.43	1.02[b]	0-3.76	1.08[a,b]	0.29-4.73	0.0239	0.4102
Blautia producta	3.73	0.42-8.17	5.24	1.32-8.07	3.3	1.33-7.66	4.66	1.83-7.63	0.4153	0.732653
g__Dorea;s__	0.12	0-1.33	0	0-0.38	0.06	0-0.75	0	0-1.01	0.9228	0.9651
g__[Ruminococcus];__	0.22	0-2.2	0.03	0-6.97	0.03	0-1.02	0.01	0-9.8	0.3439	0.732653
g__[Ruminococcus];s__	0.24	0-1.68	0.66	0.08-6.2	0.25	0-1.1	0.39	0-1.07	0.2344	0.732653
[Ruminococcus] gnavus	2.42[a]	0.79-5.61	4.48[a]	1.4-13.25	3.23[a]	1.69-8.17	7.39[a]	2.01-9.71	0.0471	0.49455
g__Peptococcus;s__	0	0-0	0	0-0.05	0.01	0-0.19	0	0-0.88	0.1299	0.68376
f__Peptostreptococcaceae;g__;s__	0.05	0-0.36	0	0-1.34	0.07	0-8.99	0	0-11.15	0.329	0.732653
f__Ruminococcaceae;g__;s__	0.6	0.06-1.55	0.91	0-1.93	0.62	0-1.64	0.44	0-1.28	0.2561	0.732653
Faecalibacterium prausnitzii	1.09	0.12-9.69	1.75	0.33-9.31	1.71	0-5.93	1.05	0.14-9.44	0.8614	0.927662
g__Ruminococcus;s__	0	0-0.18	0.02	0-0.37	0	0-0.14	0.01	0-0.1	0.4668	0.732653
g__Megamonas;s__	5.26[a]	0.58-15.36	3.39[a,b]	1.12-20.22	1.22[b]	0.32-13.44	1.68[a,b]	0.54-18.93	0.0239	0.4102
g__Phascolarctobacterium;s__	0.43	0-1.59	0.35	0.05-3.58	0.24	0-1.63	0.18	0-1.23	0.3578	0.732653
f__Erysipelotrichaceae;g__;s__	0.24	0-3.93	0.29	0-0.87	0.05	0-2.23	0.1	0-5.4	0.8145	0.900237
g__Allobaculum;s__	0.1	0-0.58	0.05	0-4.28	0.05	0-0.3	0.17	0-1.29	0.5363	0.732653
g__Catenibacterium;s__	0.31	0-1.57	0.22	0-1.19	0.05	0-0.19	0.12	0-1.35	0.5931	0.732653
[Eubacterium] biforme	0.12	0-2.33	0.07	0-1.07	0.06	0-0.27	0.08	0-1.2	0.4779	0.732653
[Eubacterium] dolichum	0.06[a]	0-0.27	0.28[a]	0-1.02	0.05[a]	0-0.66	0.05[a]	0-0.42	0.0293	0.4102
g__Fusobacterium;s__	13.51	5.21-32.62	11.39	2.26-30.43	13.09	2.15-26.04	13.06	3.24-29.49	0.5222	0.732653
g__Sutterella;s__	1.9	0.18-5.18	0.97	0.18-6.08	0.81	0.28-7.44	0.41	0.28-1.96	0.0841	0.68376
g__Campylobacter;s__	0.01	0-0.64	0	0-0	0	0-0.11	0	0-1.46	0.1439	0.68376
g__Helicobacter;s__	0.11	0-3.39	0.15	0-0.86	0.21	0-1.36	0.25	0-1.4	0.9484	0.9651
f__Succinivibrionaceae;g__;s__	0	0-1.54	0.03	0-3.09	0.04	0-6.22	0.02	0-0.79	0.4914	0.732653
f__Enterobacteriaceae;g__;s__	0.75	0.51-5.62	0.9	0.39-4.44	1.44	0.49-13.25	0.88	0.12-16	0.7892	0.895849
