# Supplementary material for: Effects of metronidazole on the fecal microbiome and metabolome in healthy dogs
Source: J Vet Intern Med. 2020 Aug 28;34(5):1853–66. doi: 10.1111/jvim.15871 (PMC7517498; doi:10.1111/jvim.15871)
Supplement: Supplementary file 3 — Supplementary Data S3. List of relevant bacterial taxa detected in fecal samples from groups 2 and 3 during the metronidazole trial, separated by taxonomic level, with median and range for each time point. Time points were compared with Friedman test, and adjusted for multiple comparison using Benjamini and Hochberg's False Discovery Rate, and p‐ and q‐values are presented. Post hoc Dunn's multiple comparison test was used to determine the bacterial taxa that were different between the time‐points, and significant differences are indicated by different superscript letters. [file JVIM-34-1853-s003.pdf]

PHYLUM

Bacterial group	Day 0		Day 7		Day 14		Day 28		Day 42		Day 0 vs Day 7 vs Day 14 vs Day 28 vs Day 42	
	Median	Range	Median	Range	Median	Range	Median	Range	Median	Range	P value	Q value
Actinobacteria	1.61[a]	0.08-6.47	5.06[b]	0.12-21.57	5.26[a,b]	0.08-17.31	1.32[a]	0-25.45	2.48[a,b]	0-10.39	0.0053	0.006625
Bacteroidetes	24.34[a]	0.89-48.6	0.73[b]	0.24-11.36	0.81[b]	0.29-13.01	15.43[a]	0-63.69	9.02[a]	0.39-31.14	0.0001	0.000167
Firmicutes	55.88	25.94-76.6	59.52	27.34-82.93	60.56	18.43-83.6	56.4	14.18-93.09	67.26	45.41-94.22	0.1117	0.1117
Fusobacteria	14.49[a]	3.24-29.49	0.62[b]	0.17-0.92	0.69[b,c]	0.14-1.41	2.54[a,c]	0.38-29.66	1.8[b,c]	0-21.56	0.0001	0.000167
Proteobacteria	3.46[a]	0.98-16.75	32.33[b]	9.13-68	29.27[b]	10.59-75.99	9.3[a]	0.84-47.21	5.91[a]	0.75-21.13	0.0001	0.000167

CLASS

Bacterial group	Day 0		Day 7		Day 14		Day 28		Day 42		Day 0 vs Day 7 vs Day 14 vs Day 28 vs Day 42	
	Median	Range	Median	Range	Median	Range	Median	Range	Median	Range	P value	Q value
Actinobacteria	0.01[a]	0-1.74	3.15[b]	0-15.95	1.28[b,c]	0-16.97	0.14[a,c]	0-12.12	0.2[a,b,c]	0-6.1	0.0001	0.000167
Coriobacteriia	1.6	0-4.73	2.32	0.12-11.33	1.44	0-7.13	1.18	0-13.32	2.06	0-5.43	0.5792	0.643556
Bacteroidia	24.34[a]	0.89-48.6	0.73[b]	0.24-11.36	0.81[b]	0.29-13.01	15.43[a]	0-63.69	9.02[a]	0.39-31.14	0.0001	0.000167
Bacilli	1.45[a]	0.57-24.12	42.82[b]	12.35-75.92	29.35[b,c]	10.54-73.54	2.05[a]	0.62-30.16	5.56[a,c]	0.66-41.72	0.0001	0.000167
Clostridia	47.47[a]	25.06-67.27	8.89[b]	4.84-48.02	22.91[b]	2.55-39.87	49.3[a]	13.06-89.18	57.55[a]	27.83-84.91	0.0001	0.000167
Erysipelotrichi	0.79[a,b]	0.03-7.05	0.27[a,b]	0-9.34	0.19[a]	0-7.09	0.53[a,b]	0.16-5.99	1.3[b]	0.2-8.3	0.0234	0.033429
Fusobacteriia	14.49[a]	3.24-29.49	0.62[b]	0.17-0.92	0.69[b,c]	0.14-1.41	2.54[a,c]	0.38-29.66	1.8[b,c]	0-21.56	0.0001	0.000167
Betaproteobacteria	0.96	0.12-4.37	0.71	0.21-12.58	0.73	0.24-3.65	0.7	0-9.94	0.64	0.12-5.36	0.7541	0.7541
Epsilonproteobacteria	0.19	0-2.24	0.1	0.02-0.31	0.11	0-0.4	0.16	0-18.55	0.12	0-1.42	0.0704	0.088
Gammaproteobacteria	0.96[a]	0-16.17	27.91[b]	8.86-64.33	26.5[b]	9.46-72.24	4.4[a]	0.6-45.01	3[a]	0.53-19.89	0.0001	0.000167

ORDER

Bacterial group	Day 0		Day 7		Day 14		Day 28		Day 42		Day 0 vs Day 7 vs Day 14 vs Day 28 vs Day 42	
	Median	Range	Median	Range	Median	Range	Median	Range	Median	Range	P value	Q value

Bifidobacteriales	0.01[a]	0-1.74	2.93[b]	0-15.95	1.15[a,b]	0-16.97	0.14[a,b]	0-12.12	0.2[a,b]	0-6.1	0.0002	0.000433
Coriobacteriales	1.6	0-4.73	2.32	0.12-11.33	1.44	0-7.13	1.18	0-13.32	2.06	0-5.43	0.5792	0.684509
Bacteroidales	24.34[a]	0.89-48.6	0.73[b]	0.24-11.36	0.81[b]	0.29-13.01	15.43[a]	0-63.69	9.02[a]	0.39-31.14	0.0001	0.00026
Bacillales	0[a]	0-0.55	0.28[a]	0-7.36	0.04[a]	0-3.93	0[a]	0-0.16	0[a]	0-1.07	0.0124	0.023029
Lactobacillales	1.29[a]	0.57-23.89	42.46[b]	5.11-75.59	28.99[b]	8.91-73.12	0.99[a]	0.62-29.97	4.92[a]	0.51-41.72	0.0001	0.00026
Turicibacterales	0.1	0-6.03	0.18	0-0.45	0.21	0-0.33	0.22	0-1.96	0.16	0-2.54	0.318	0.4134
Clostridiales	47.47[a]	25.06-67.27	8.89[b]	4.84-48.02	22.91[b]	2.55-39.87	49.3[a]	13.06-89.18	57.55[a]	27.83-84.91	0.0001	0.00026
Erysipelotrichales	0.79[a,b]	0.03-7.05	0.27[a,b]	0-9.34	0.19[a]	0-7.09	0.53[a,b]	0.16-5.99	1.3[b]	0.2-8.3	0.0234	0.038025
Fusobacteriales	14.49[a]	3.24-29.49	0.62[b]	0.17-0.92	0.69[b,c]	0.14-1.41	2.54[a,c]	0.38-29.66	1.8[b,c]	0-21.56	0.0001	0.00026
Burkholderiales	0.96	0.12-4.37	0.71	0.21-12.58	0.73	0.24-3.65	0.7	0-9.94	0.64	0.12-5.36	0.7541	0.7541
Campylobacterales	0.19	0-2.24	0.1	0.02-0.31	0.11	0-0.4	0.16	0-18.55	0.12	0-1.42	0.0704	0.101689
Aeromonadales	0.07	0-3.13	0.08	0-2.31	0.06	0-1.67	0.09	0-6.6	0.08	0-2.17	0.7502	0.7541
Enterobacteriales	0.68[a]	0-16	27.81[b]	8.76-64.25	26.44[b]	9.41-72.15	2.12[a]	0.04-44.9	1.97[a]	0.45-19.83	0.0001	0.00026

FAMILY

Bacterial group	Day 0		Day 7		Day 14		Day 28		Day 42		Day 0 vs Day 7 vs Day 14 vs Day 28 vs Day 42	
	Median	Range	Median	Range	Median	Range	Median	Range	Median	Range	P value	Q value
Bifidobacteriaceae	0.01[a]	0-1.74	2.93[b]	0-15.95	1.15[a,b]	0-16.97	0.14[a,b]	0-12.12	0.2[a,b]	0-6.1	0.0002	0.000382
Coriobacteriaceae	1.6	0-4.73	2.32	0.12-11.33	1.44	0-7.13	1.18	0-13.32	2.06	0-5.43	0.5792	0.640168
Bacteroidaceae	20.62[a]	0.79-48.42	0.6[b]	0.22-4.43	0.66[b]	0.29-12.75	15.37[a]	0-63.18	8.36[a]	0.39-31.1	0.0001	0.00021
Prevotellaceae	0.18[a]	0-7.34	0.08[b]	0-10.45	0.06[b]	0-9.76	0.18[a,b]	0-0.24	0.16[a,b]	0-5.85	0.0051	0.008238
[Paraprevotellaceae]	0.2[a]	0-3.82	0.03[b]	0-1.24	0.02[b]	0-0.15	0.07[a,b]	0-2.24	0.15[a,b]	0-1.7	0.0015	0.002625
Enterococcaceae	0.27[a]	0-1.17	10.01[b]	0.98-49.33	6.9[b]	0.95-27.36	0.34[a]	0-3.85	0.31[a]	0-15.09	0.0001	0.00021
Lactobacillaceae	0.14	0-21.28	0.27	0-59.74	0.23	0-17.88	0.12	0-4.23	0.12	0-16.86	0.4982	0.581233
Streptococcaceae	0.51[a]	0.33-19.29	12.29[b]	0.47-58.66	19.97[b,d]	2.01-64.89	0.53[a,c]	0-29.08	0.67[a,c,d]	0.42-41.4	0.0001	0.00021
Turicibacteraceae	0.1	0-6.03	0.18	0-0.45	0.21	0-0.33	0.22	0-1.96	0.16	0-2.54	0.318	0.392824
o__Clostridiales;f__	0.33[a,b]	0-1.71	0[a]	0-0.75	0[a]	0-4.34	0.23[b]	0-2.19	0.31[b]	0-6.48	0.0001	0.00021
Clostridiaceae	10.89	4.47-41.14	6.34	1.19-46.38	8.16	1.35-32.85	13.05	2.19-29.95	9.36	3.67-22.53	0.2532	0.332325
Lachnospiraceae	19.91[a,d]	11.84-39.78	1.28[b]	0.32-10	1.27[a,b]	0-34.3	25.13[a,d]	8.42-85.45	29.85[d]	12.19-63.22	0.0001	0.00021

Peptostreptococcaceae	$0^{a,b,c}$	0-11.15	0^{a}	0-0.1	0^{a}	0-14.85	$0.22^{b,c}$	0-2.22	0.37^{b}	0-4.37	0.0001	0.00021
Ruminococcaceae	2.05^{a}	0.27-11.26	0.12^{b}	0-0.53	0.14^{b}	0-4.48	$0.36^{a,b}$	0.09-6.48	1.2^{a}	0.21-5.75	0.0001	0.00021
Veillonellaceae	7.83^{a}	0.6-20.02	$0.39^{b,c}$	0.22-0.98	$0.41^{b,c}$	0.2-1.53	$0.55^{c,d}$	0.23-26.11	$1.51^{a,d}$	0.24-32.23	0.0001	0.00021
Erysipelotrichaceae	$0.79^{a,b}$	0.03-7.05	$0.27^{a,b}$	0-9.34	0.19^{a}	0-7.09	$0.53^{a,b}$	0.16-5.99	1.3^{b}	0.2-8.3	0.0234	0.0351
Fusobacteriaceae	14.49^{a}	3.24-29.49	0.62^{b}	0.17-0.92	$0.69^{b,c}$	0.14-1.41	$2.54^{a,c}$	0.38-29.66	$1.8^{b,c}$	0-21.56	0.0001	0.00021
Alcaligenaceae	0.96	0.12-4.37	0.71	0.21-12.58	0.73	0.24-3.65	0.7	0-9.94	0.64	0.12-5.36	0.7541	0.7541
Helicobacteraceae	0.14	0-1.49	0.1	0-0.28	0.11	0-0.4	0.12	0-18.55	0.1	0-1.42	0.053	0.0742
Succinivibrionaceae	0.07	0-3.13	0.08	0-2.31	0.06	0-1.67	0.09	0-6.6	0.08	0-2.17	0.7502	0.7541
Enterobacteriaceae	0.68^{a}	0-16	27.81^{b}	8.76-64.25	26.44^{b}	9.41-72.15	2.12^{a}	0.04-44.9	1.97^{a}	0.45-19.83	0.0001	0.00021

GENUS

Bacterial group	Day 0		Day 7		Day 14		Day 28		Day 42		Day 0 vs Day 7 vs Day 14 vs Day 28 vs Day 42	
	Median	Range	Median	Range	Median	Range	Median	Range	Median	Range	P value	Q value
Bifidobacterium	0.01^{a}	0-1.74	2.93^{b}	0-15.95	$1.15^{a,b}$	0-16.97	$0.14^{a,b}$	0-12.12	$0.2^{a,b}$	0-6.1	0.0002	0.000412
Collinsella	1.57	0-4.47	2.2	0.12-11.33	1.44	0-7.13	0.98	0-13.32	2.04	0-4.91	0.5594	0.575853
Bacteroides	20.62^{a}	0.79-48.37	0.6^{b}	0.22-4.43	0.66^{b}	0.29-12.75	15.36^{a}	0-63.18	8.34^{a}	0.39-31.07	0.0001	0.00025
Prevotella	0.18^{a}	0-7.34	0.08^{b}	0-10.45	0.06^{b}	0-9.76	$0.18^{a,b}$	0-0.24	$0.16^{a,b}$	0-5.85	0.0051	0.00714
[Prevotella]	0.2^{a}	0-3.82	0.03^{b}	0-1.24	0.02^{b}	0-0.15	$0.07^{a,b}$	0-2.24	$0.15^{a,b}$	0-1.7	0.0015	0.002625
Enterococcus	0.27^{a}	0-1.17	9.79^{b}	0.98-49.33	6.9^{b}	0.95-27.36	0.34^{a}	0-3.42	0.31^{a}	0-15.09	0.0001	0.00025
Lactobacillus	0.14	0-21.28	0.27	0-59.74	0.23	0-17.88	0.12	0-4.23	0.12	0-16.86	0.4982	0.532
Streptococcus	0.51^{a}	0.33-19.29	12.29^{b}	0.47-58.66	19.97^{b}	2.01-64.89	0.52^{a}	0-29.08	$0.67^{a,b}$	0.42-41.4	0.0001	0.00025
Turicibacter	0.1	0-6.03	0.18	0-0.45	0.21	0-0.33	0.22	0-1.96	0.16	0-2.54	0.318	0.371
o__Clostridiales;f__;g__	$0.33^{a,b}$	0-1.71	0^{a}	0-0.75	0^{a}	0-4.34	0.23^{b}	0-2.19	0.31^{b}	0-6.48	0.0001	0.00025
f__Clostridiaceae;__	10.48^{a}	4.28-30.94	1.09^{b}	0.37-13.05	3.74^{b}	0.24-18.69	$6.19^{a,b}$	1.11-23.66	$6.55^{a,b}$	1.08-21.53	0.0002	0.000412
f__Clostridiaceae;g__	0.26^{a}	0-9.7	3.16^{b}	0.25-33.33	3.55^{b}	0.23-17.02	2.35^{b}	0-11.51	$1.21^{a,b}$	0-5.28	0.002	0.003333
Clostridium	0.12	0-5	0	0-9.77	0	0-6.45	0.5	0-4.27	0.47	0-10.95	0.197	0.24625
f__Lachnospiraceae;__	6^{a}	3.14-14.32	0.43^{b}	0-1.8	0.44^{b}	0-1.92	5.27^{a}	0.49-32.52	8.74^{a}	1.37-17.77	0.0001	0.00025
f__Lachnospiraceae;g__	1.74^{a}	0.18-4.15	0^{b}	0-0.97	0^{b}	0-0.84	1.03^{a}	0.02-5.14	1.35^{a}	0-9.57	0.0001	0.00025
Blautia	$6.64^{a,b}$	2.28-9.89	0.48^{a}	0.21-4.96	0.46^{a}	0-13.55	10.91^{b}	2.42-44.78	14.94^{b}	3.62-28.3	0.0001	0.00025

Dorea	$0^{a,b,c}$	0-1.01	0^{a}	0-0	$0^{a,c}$	0-0.13	$0.77^{b,c}$	0-7.66	0.64^{b}	0-8.35	0.0001	0.00025
[Ruminococcus]	$3.07^{a,c}$	1.72-15.7	0.5^{b}	0-6.87	$0.54^{a,b}$	0-25.2	$4.89^{a,b,c}$	1.44-24.25	4.36^{c}	1.86-38.88	0.0003	0.000553
f__Peptostreptococcaceae;g__	$0^{a,b}$	0-11.15	0^{a}	0-0.1	$0^{a,b}$	0-14.85	$0.19^{a,b}$	0-2.22	0.37^{b}	0-2.2	0.0001	0.00025
f__Ruminococcaceae;g__	0.43^{a}	0-1.28	0^{b}	0-0.23	0^{b}	0-0.3	$0.01^{a,b}$	0-0.49	0.36^{a}	0-4.34	0.0001	0.00025
Faecalibacterium	1.53^{a}	0-9.44	0.11^{b}	0-0.21	$0.13^{a,b}$	0-4.07	$0.16^{a,b}$	0-5.96	$0.2^{a,b}$	0.11-4.91	0.0039	0.005935
Oscillospira	0^{a}	0-0.45	0^{a}	0-0	0^{a}	0-0.38	0^{a}	0-0.63	0.01^{a}	0-1.51	0.0023	0.003659
Ruminococcus	0.04	0-0.1	0	0-0.32	0	0-0.86	0.01	0-0.29	0.06	0-0.2	0.3758	0.42429
Megamonas	5.23^{a}	0.54-19.96	0.35^{b}	0.22-0.68	0.39^{b}	0.2-0.76	$0.51^{b,c}$	0.23-26.09	$0.72^{a,c}$	0.24-31.84	0.0001	0.00025
Phascolarctobacterium	0.04	0-1.23	0	0-0.25	0	0-0.07	0	0-0.61	0	0-3.36	0.0654	0.084778
f__Erysipelotrichaceae;g__	$0.12^{a,b,c}$	0-5.4	0^{a}	0-0.4	$0.01^{a,c}$	0-0.23	$0.16^{b,c}$	0-1.85	0.2^{b}	0-2.07	0.0002	0.000412
Allobaculum	0.09	0-1.29	0	0-3.31	0	0-7.06	0	0-2.21	0	0-2.97	0.2596	0.31331
Catenibacterium	0.16^{a}	0-2.39	$0.07^{a,b}$	0-0.77	0^{b}	0-0.32	0^{b}	0-0.23	$0^{a,b}$	0-1.14	0.0003	0.000553
[Eubacterium]	$0.23^{a,b}$	0-2.95	0.03^{a}	0-8.27	$0.05^{a,b}$	0-5.36	$0.2^{a,b}$	0-4.42	0.66^{b}	0.06-4.5	0.0047	0.006854
Fusobacterium	$14.46^{a,c}$	3.24-29.49	0.6^{b}	0.17-0.92	$0.69^{b,c}$	0.14-1.2	1.02^{c}	0.38-29.66	$1.2^{b,c}$	0-21.56	0.0001	0.00025
Sutterella	0.96	0.12-4.37	0.71	0.21-12.58	0.73	0.24-3.65	0.7	0-9.94	0.64	0.12-5.36	0.7541	0.7541
Helicobacter	0.14	0-1.49	0.1	0-0.28	0.11	0-0.4	0.12	0-18.55	0.09	0-1.42	0.0598	0.0805
f__Succinivibrionaceae;g__	0.02	0-0.79	0.06	0-2.31	0.06	0-1.46	0.07	0-2.19	0.07	0-0.92	0.5016	0.532
f__Enterobacteriaceae;g__	0.68^{a}	0-16	24.89^{b}	8.63-63.53	26.09^{b}	9.41-66.66	2.12^{a}	0-44.9	1.97^{a}	0.45-19.83	0.0001	0.00025
Proteus	0^{a}	0-0.03	0.09^{a}	0-8.04	0^{a}	0-5.84	0^{a}	0-3.31	0^{a}	0-0	0.0001	0.00025

SPECIES

Bacterial group	Day 0		Day 7		Day 14		Day 28		Day 42		Day 0 vs Day 7 vs Day 14 vs Day 28 vs Day 42	
	Median	Range	Median	Range	Median	Range	Median	Range	Median	Range	P value	Q value
g__Bifidobacterium;s__	0.01^{a}	0-1.74	0.15^{a}	0-15.95	0.22^{a}	0-16.97	0^{a}	0-12.12	0.19^{a}	0-6.1	0.015	0.021207
Collinsella stercoris	1.57	0-4	2.2	0.12-11.33	1.44	0-7.13	0.98	0-13.32	1.91	0-4.91	0.4786	0.516384
g__Bacteroides;__	7.14^{a}	0.2-26.84	0.3^{b}	0-0.59	0.3^{b}	0-1.85	0.56^{b}	0-12.71	$0.64^{a,b}$	0-5.69	0.0001	0.000228
g__Bacteroides;s__	5.67^{a}	0.4-27.92	0.2^{b}	0-3.84	0.22^{b}	0.06-11.98	2.74^{a}	0-61.19	4.95^{a}	0.02-26.31	0.0001	0.000228
Bacteroides plebeius	2.61^{a}	0-8.39	0.11^{b}	0-0.19	$0.11^{b,c}$	0-0.34	$0.29^{a,c}$	0-9.84	$0.33^{a,c}$	0-11.6	0.0001	0.000228
Prevotella copri	0.18^{a}	0-7.34	0.08^{b}	0-10.45	0.06^{b}	0-9.76	0.18^{a}	0-0.24	0.16^{a}	0-5.85	0.0051	0.007744

g__[Prevotella];s__	0.2[a]	0-3.82	0.03[b]	0-1.24	0.02[b]	0-0.15	0.07[a,b]	0-2.24	0.15[a,b]	0-1.7	0.0015	0.002795
g__Enterococcus;s__	0.27[a]	0-1.17	9.79[b]	0.98-49.03	6.13[b]	0.95-26.58	0.34[a]	0-3.42	0.31[a]	0-15.09	0.0001	0.000228
g__Lactobacillus;s__	0.14	0-21.28	0.27	0-59.74	0.23	0-17.88	0.12	0-3.39	0.12	0-16.86	0.3983	0.441359
g__Streptococcus;s__	0.51[a]	0.33-19.29	12.29[b]	0.47-58.58	19.97[b]	2.01-64.83	0.52[a]	0-29.08	0.67[a,b]	0.42-41.4	0.0001	0.000228
g__Turicibacter;s__	0.1	0-6.03	0.18	0-0.45	0.21	0-0.33	0.22	0-1.96	0.16	0-2.54	0.318	0.372514
o__Clostridiales;f__;g__;s__	0.33[a,b]	0-1.71	0[a]	0-0.75	0[a]	0-4.34	0.23[b]	0-2.19	0.31[b]	0-6.48	0.0001	0.000228
f__Clostridiaceae;__;__	10.48[a]	4.28-30.94	1.09[b]	0.37-13.05	3.74[b]	0.24-18.69	6.19[a,b]	1.11-23.66	6.55[a,b]	1.08-21.53	0.0002	0.00041
f__Clostridiaceae;g__;s__	0.26[a]	0-9.7	3.16[b]	0.25-33.33	3.55[b]	0.23-17.02	2.35[b]	0-11.51	1.21[a,b]	0-5.28	0.002	0.003565
g__Clostridium;__	0	0-1.85	0	0-9.77	0	0-2.15	0.2	0-1.63	0	0-10.95	0.1581	0.196427
Clostridium perfringens	0.04[a]	0-3.15	0[a]	0-0	0[a]	0-5.96	0[a]	0-4.07	0.1[a]	0-4.02	0.0056	0.0082
f__Lachnospiraceae;__;__	6[a]	3.14-14.32	0.43[b]	0-1.8	0.44[b]	0-1.92	5.27[a]	0.49-32.52	8.74[a]	1.37-17.77	0.0001	0.000228
f__Lachnospiraceae;g__;s__	1.74[a]	0.18-4.15	0[b]	0-0.97	0[b]	0-0.84	1.03[a]	0.02-5.14	1.35[a]	0-9.57	0.0001	0.000228
g__Blautia;s__	1.43[a,c]	0-4.73	0[b]	0-0.99	0[b,c]	0-4.27	1.77[a]	0-16.39	6.06[a]	0-14.48	0.0001	0.000228
Blautia producta	4.45[a,c]	1.83-7.63	0.48[b]	0-3.97	0.43[b,c]	0-13.1	5.79[a,c]	1.75-43.29	7.4[a]	1.67-19.32	0.0001	0.000228
g__Dorea;s__	0[a,b,c]	0-1.01	0[a]	0-0	0[a,c]	0-0.13	0.77[b,c]	0-7.66	0.64[b]	0-8.35	0.0001	0.000228
g__[Ruminococcus];s__	0.33[a]	0-1.07	0[b]	0-0.15	0[b]	0-0.29	0.17[a,b]	0-14.85	0.71[a]	0.1-35.78	0.0001	0.000228
[Ruminococcus] gnavus	2.84[a]	1.32-9.71	0.47[b]	0-6.87	0.46[a,b]	0-25.2	3.83[a,b]	1.25-9.17	2.67[a,b]	1.5-14.79	0.0038	0.00615
f__Peptostreptococcaceae;g__;s__	0[a,b]	0-11.15	0[a]	0-0.1	0[a,b]	0-14.85	0.19[a,b]	0-2.22	0.37[b]	0-2.2	0.0001	0.000228
f__Ruminococcaceae;g__;s__	0.43[a]	0-1.28	0[b]	0-0.23	0[b]	0-0.3	0.01[a,b]	0-0.49	0.36[a]	0-4.34	0.0001	0.000228
Faecalibacterium prausnitzii	1.53[a]	0-9.44	0.11[b]	0-0.21	0.13[a,b]	0-4.07	0.16[a,b]	0-5.96	0.2[a,b]	0.11-4.91	0.0039	0.00615
g__Oscillospira;s__	0[a]	0-0.45	0[a]	0-0	0[a]	0-0.38	0[a]	0-0.63	0.01[a]	0-1.51	0.0023	0.003929
g__Ruminococcus;s__	0.04	0-0.1	0	0-0.32	0	0-0.86	0.01	0-0.29	0.06	0-0.2	0.3758	0.427994
g__Megamonas;s__	5.23[a]	0.54-19.96	0.35[b]	0.22-0.68	0.39[b]	0.2-0.76	0.51[b,c]	0.23-26.09	0.72[a,c]	0.24-31.84	0.0001	0.000228
g__Phascolarctobacterium;s__	0.04	0-1.23	0	0-0.25	0	0-0.07	0	0-0.61	0	0-3.36	0.0654	0.083794
f__Erysipelotrichaceae;g__;s__	0.12[a,b,c]	0-5.4	0[a]	0-0.4	0.01[a,c]	0-0.23	0.16[b,c]	0-1.85	0.2[b]	0-2.07	0.0002	0.00041
g__Allobaculum;s__	0.09	0-1.29	0	0-3.31	0	0-7.06	0	0-2.21	0	0-2.97	0.2596	0.313047
g__Catenibacterium;s__	0.16[a]	0-2.39	0.07[a,b]	0-0.77	0[b]	0-0.32	0[b]	0-0.23	0[a,b]	0-1.14	0.0003	0.000586
[Eubacterium] biforme	0.08	0-2.95	0.03	0-8.04	0.03	0-5.27	0.07	0-4.37	0.15	0-4.15	0.6562	0.672605
[Eubacterium] dolichum	0.05[a]	0-0.42	0[a]	0-0.44	0[a]	0-1.06	0.08[a]	0-2.32	0.08[a]	0-2.59	0.027	0.0369
g__Fusobacterium;s__	14.46[a]	3.24-29.49	0.6[b]	0.17-0.92	0.69[b,c]	0.14-1.2	1.02[a,c]	0.38-29.66	1.2[b,c]	0-21.56	0.0001	0.000228
g__Sutterella;s__	0.96	0.12-4.37	0.71	0.21-12.58	0.73	0.24-3.65	0.7	0-9.94	0.64	0.12-5.36	0.7541	0.7541

g__Helicobacter;s__	0.14	0-1.4	0.1	0-0.28	0.1	0-0.4	0.12	0-18.55	0.09	0-0.95	0.0573	0.075784
f__Succinivibrionaceae;g__;s__	0.02	0-0.79	0.06	0-2.31	0.06	0-1.46	0.07	0-2.19	0.07	0-0.92	0.5016	0.527323
f__Enterobacteriaceae;g__;s__	0.68[a]	0-16	24.89[b]	8.63-63.53	26.09[b]	9.41-66.66	2.12[a]	0-44.9	1.97[a]	0.45-19.83	0.0001	0.000228
g__Proteus;s__	0[a]	0-0.03	0.09[a]	0-8.04	0[a]	0-5.84	0[a]	0-3.31	0[a]	0-0	0.0001	0.000228
