# Supplementary material for: Effects of metronidazole on the fecal microbiome and metabolome in healthy dogs
Source: J Vet Intern Med. 2020 Aug 28;34(5):1853–66. doi: 10.1111/jvim.15871 (PMC7517498; doi:10.1111/jvim.15871)
Supplement: Supplementary file 4 — Supplementary Data S4. List of relevant metabolites detected in fecal samples from group 1 (control), with mean and SD for each time point. Time points were compared with 1‐way ANOVA and adjusted for multiple comparison using Benjamini and Hochberg's False Discovery Rate, and p‐ and q‐values are presented. [file JVIM-34-1853-s004.pdf]

Group 1 Summary Statistics for fecal metabolomics

	day 0		day 7		day 21		day 42			
**Compound name**	**mean**	**standard deviation**	**mean**	**standard deviation**	**mean**	**standard deviation**	**mean**	**standard deviation**	**p-value**	**q-value**
xylulose NIST	10229	8625	12696	16420	8960	6103	7817	5730	0.7482	0.9428
xylose	519348	565805	686314	744163	468380	433454	427240	349741	0.6829	0.9428
xylitol	841	631	1016	729	1141	1535	612	321	0.6740	0.9428
xanthosine	369	110	276	82	340	150	273	124	0.3331	0.9428
xanthine	5237	4569	5983	4423	4233	2455	5703	3264	0.8122	0.9635
vanillic acid	856	929	471	293	931	1164	909	1064	0.6989	0.9428
valine	159705	103419	146034	71601	161353	98885	160134	120477	0.9912	0.9995
urocanic acid	865	197	1156	831	929	503	1104	747	0.7870	0.9614
uridine	1585	831	1501	1625	958	148	701	230	0.2074	0.9428
uric acid	2166	2725	890	503	1631	1049	1509	2466	0.5577	0.9428
urea	2068	764	1689	262	2145	1145	1472	639	0.1821	0.9428
uracil	38645	30040	30424	14932	33940	18392	35762	26901	0.8537	0.9672
UDP-glucuronic acid	1005	573	717	313	759	489	852	551	0.6621	0.9428
tyrosine	126202	58890	165056	93366	144885	65405	140853	69020	0.7645	0.9501
tyramine	50482	45995	93705	68601	33083	30670	125948	91296	0.0331	0.9264
tryptophan	33782	22775	32433	25037	21353	14436	33259	20328	0.5433	0.9428
trehalose	2742	3679	1359	1567	14734	36271	1886	2118	0.4171	0.9428
trans-4-hydroxyproline	9604	5711	10483	7774	8927	6304	12740	7386	0.6117	0.9428
thymine	12237	5002	9754	3396	12507	7519	11199	7395	0.7719	0.9538
thymidine	1765	1900	1336	1115	1108	808	1018	554	0.2032	0.9428
threonine	18736	7075	18993	5147	19801	15280	17094	7593	0.9546	0.9995
threonic acid	308	121	238	53	279	87	295	106	0.3963	0.9428
threitol	1364	1251	1035	814	1322	1078	954	870	0.5133	0.9428
taurine	174133	481842	7220	11390	7648	17133	4268	7135	0.4305	0.9428
tagatose	978	451	653	284	575	243	688	384	0.1332	0.9428
sucrose	165	111	187	154	179	94	213	127	0.8689	0.9672
stearic acid	127158	92223	90116	30147	104815	28562	93973	58136	0.6484	0.9428
spermidine	7218	4252	6155	3673	6635	4245	8453	5435	0.6298	0.9428
sinapinic acid	633	602	1109	1284	860	1108	1004	1177	0.4942	0.9428

shikimic acid	1976	1257	1793	1177	1238	587	1692	1281	0.4499	0.9428
serine	27195	13269	28196	12098	30222	17555	19685	8243	0.4533	0.9428
sebacic acid, di(2-octyl) ester NIST	410	196	418	290	507	294	332	240	0.6331	0.9428
salicylaldehyde	2212	1768	1215	979	2571	2331	2532	2724	0.5029	0.9428
ribose	102968	79391	88530	25749	91562	58778	97056	67867	0.9578	0.9995
ribonic acid	325	111	598	801	353	74	325	135	0.4950	0.9428
ribitol	2913	1470	2470	1599	2067	1357	3333	2717	0.2919	0.9428
raffinose	325	171	281	143	242	56	228	144	0.3793	0.9428
pyruvic acid	2305	852	3583	2877	5116	8604	1809	1033	0.5276	0.9428
putrescine	179900	192610	183889	126252	148638	50232	190884	116914	0.8179	0.9635
pseudo uridine	2103	1434	1993	1016	1655	534	1732	1354	0.8488	0.9672
propane-1,3-diol NIST	763	360	1376	855	1800	1650	3279	4033	0.1670	0.9428
proline	92340	77442	63039	27244	66223	47124	64292	46462	0.6972	0.9428
piperidone	67360	39626	91903	63089	90299	76165	100408	53224	0.6001	0.9428
pipecolinic acid	25179	30712	11240	7972	20419	16844	11790	6634	0.3639	0.9428
pinitol	330	120	312	239	277	76	15538	42757	0.4057	0.9428
pimelic acid	809	1067	388	140	491	317	407	139	0.3643	0.9428
phosphate	1137	762	1317	305	4661	6537	1622	1215	0.1419	0.9428
phenylpyruvate	726	388	621	305	796	540	764	545	0.8772	0.9672
phenylethylamine	3502	3565	5629	5452	4459	5527	6995	10654	0.3367	0.9428
phenylalanine	67595	24686	89396	30649	92735	41312	90111	56993	0.6165	0.9428
phenylacetic acid	26610	34077	15701	18560	20166	20455	5380	4924	0.2433	0.9428
phenol	2946	2459	1910	1093	3335	1788	3363	3371	0.5127	0.9428
pentitol	303	141	248	81	246	34	230	98	0.4810	0.9428
pentadecanoic acid	2891	1252	3036	1311	6235	8281	4229	2498	0.3963	0.9428
parabanic acid NIST	1713	1249	1600	1203	2097	1734	2280	2567	0.8291	0.9635
pantothenic acid	2537	2662	2189	1150	1401	649	1940	1163	0.5675	0.9428
palmitic acid	26918	13354	25094	9889	24877	5491	28577	22660	0.9535	0.9995
oxoproline	32601	23173	32332	21177	31802	15100	32911	14428	0.9995	0.9995
ornithine	21765	16935	26683	13938	21152	16116	29688	28998	0.6170	0.9428
oleic acid	1198	2429	623	1110	735	1436	264	171	0.6441	0.9428
octadecanol	502	212	370	146	386	139	384	230	0.5381	0.9428
O-acetylserine	806	402	829	243	1045	446	969	541	0.5792	0.9428
norvaline	3145	1947	5389	6491	2910	1032	5269	3999	0.4367	0.9428

N-methylalanine	76989	40075	68426	34704	55930	27434	78275	54763	0.7414	0.9428
nicotinic acid	18588	7464	18499	8592	15924	9402	17678	10030	0.8737	0.9672
nicotianamine	341	98	259	45	249	59	355	208	0.1460	0.9428
N-acetylputrescine	2169	2068	3272	1758	2271	1471	1622	483	0.1778	0.9428
N-acetylornithine	2604	2014	3018	2257	2227	1814	2076	2163	0.4252	0.9428
N-acetyl-D-mannosamine	7764	6421	2400	1412	3147	2425	2747	1742	0.0082	0.9264
N-acetyl-D-hexosamine	946	314	759	197	1071	505	868	229	0.2668	0.9428
N-acetyl-D-galactosamine	21475	14963	9644	4116	12798	9240	11027	7365	0.0184	0.9264
myristic acid	7592	4766	9429	6479	9154	6657	8337	4428	0.9128	0.9911
myo-inositol	1259	681	1276	1180	1312	815	1320	900	0.9978	0.9995
montanic acid	1009	484	1029	725	842	283	599	206	0.2329	0.9428
methyltetrahydrophenanthrenone NIST	1761	701	1562	560	1661	728	1650	1010	0.9692	0.9995
methyl O-D-galactopyranoside	1941	863	1374	903	1271	629	1310	1148	0.4903	0.9428
methionine sulfoxide	5166	3316	7046	6416	4874	2710	6279	3764	0.6976	0.9428
methionine	22859	10512	20344	12691	19667	17855	16323	9965	0.8153	0.9635
methanolphosphate	2088	1912	1904	1163	2748	2151	2640	1635	0.7444	0.9428
melezitose	259	79	176	69	257	77	236	142	0.2077	0.9428
maltotriose	405	231	223	58	234	83	242	172	0.0141	0.9264
maltose	11746	17666	5185	7351	13183	14719	5339	8717	0.2223	0.9428
maltitol	407	435	454	302	313	69	355	172	0.6138	0.9428
malonic acid	308	72	230	51	244	121	218	102	0.2938	0.9428
malic acid	2475	2529	791	485	870	593	801	447	0.0413	0.9428
maleimide	2521	1806	2039	906	2288	1780	1840	1172	0.7398	0.9428
maleic acid	381	159	283	54	299	109	305	82	0.2709	0.9428
lyxose	24886	25628	40091	50226	28884	24512	24196	22346	0.6729	0.9428
lyxitol	3491	1982	3242	1187	2961	1592	3727	1965	0.6504	0.9428
lysine	103995	66856	170424	95519	120296	72168	151174	94167	0.2193	0.9428
lithocholic acid	1265	1576	429	498	572	347	311	110	0.0916	0.9428
linoleic acid	499	297	210	135	374	133	309	155	0.0345	0.9264
lignoceric acid	1127	1652	377	117	492	186	421	223	0.2709	0.9428
levoglucosan	1212	1855	966	520	3797	7279	540	526	0.3350	0.9428
leucine	177712	100242	156714	73518	173395	115710	193535	166723	0.9512	0.9995
lactulose	1929	2226	997	1108	6095	14685	4635	6662	0.4860	0.9428
lactose	656	235	595	397	1330	2086	728	558	0.5372	0.9428

lactitol	2105	2100	1854	2080	2356	4311	1528	1719	0.7543	0.9428
kynurenic acid	748	856	576	437	601	330	851	796	0.8095	0.9635
isothreonic acid	817	713	577	464	534	354	476	300	0.3025	0.9428
isomaltose	340	166	308	143	357	93	382	197	0.8247	0.9635
isoleucine	112902	74377	106802	66009	112555	79825	115924	107106	0.9976	0.9995
inosine	1742	1416	1340	1363	1048	863	941	688	0.1227	0.9428
indole-3-lactate	36628	43270	28203	21767	38447	24615	28221	18450	0.6932	0.9428
indole-3-acetate	3177	2357	4827	4767	3556	1635	2727	1491	0.5593	0.9428
hypoxanthine	9338	7976	11213	5060	8270	5442	8708	3909	0.7438	0.9428
hydroxylamine	68439	53236	49739	26496	75414	46870	61993	33508	0.6377	0.9428
hydroxycarbamate NIST	13621	10542	12226	6265	17769	13152	14689	7147	0.6433	0.9428
homoserine	1630	708	1303	692	1376	545	1694	718	0.3097	0.9428
homocystine	520	170	383	95	496	189	405	135	0.0746	0.9428
histidine	8347	5229	14688	7154	9271	5340	12099	7294	0.1155	0.9428
hexuronic acid	9356	20235	1394	820	1521	1031	3052	3844	0.4126	0.9428
hexonic acid	473	279	601	273	632	298	561	324	0.6900	0.9428
hexitol	434	119	461	138	389	63	470	337	0.8631	0.9672
heptadecanoic acid	2317	1871	1413	687	1757	641	1406	525	0.2286	0.9428
guanosine	752	530	532	304	529	358	337	129	0.0168	0.9264
guanine	924	546	620	264	672	323	452	201	0.0269	0.9264
glycyl tyrosine	702	380	717	508	640	339	637	245	0.9122	0.9911
glycyl proline	7418	4598	11296	8275	6552	4576	9411	4876	0.1903	0.9428
glycolic acid	17476	22983	9168	3716	9857	5164	8054	4369	0.3588	0.9428
glycine	42585	20268	34150	15764	37376	25181	38338	18311	0.8876	0.9736
glycerol-alpha-phosphate	773	688	625	563	622	389	553	366	0.8232	0.9635
glycerol-3-galactoside	868	539	488	301	604	364	687	420	0.1943	0.9428
glycerol	35384	14010	34478	14701	33070	23383	28313	12605	0.7373	0.9428
glyceric acid	5198	2218	4427	2197	5414	2442	2558	1397	0.0466	0.9428
glutaric acid	889	749	653	515	660	426	586	220	0.6524	0.9428
glutamine	9339	6942	11527	11686	11122	11512	11292	5212	0.9592	0.9995
glutamic acid	74209	55271	100506	103526	66989	49567	66248	16726	0.6979	0.9428
glucose-1-phosphate	1057	348	1500	745	1095	505	980	406	0.2203	0.9428
glucose	50824	30088	36671	25021	41010	29128	53550	50394	0.6408	0.9428
glucoheptulose	1093	654	607	205	748	680	609	456	0.1479	0.9428

galacturonic acid	4267	4692	1354	1275	918	619	1071	1073	0.0314	0.9264
galactitol	1677	1636	657	259	621	228	673	524	0.0585	0.9428
galactinol	659	761	408	202	288	61	418	352	0.3151	0.9428
fumaric acid	3693	2258	4343	5126	2873	2026	4110	2900	0.7178	0.9428
fucose	61430	18665	39366	16373	42835	25592	57210	67805	0.5626	0.9428
fructose	5327	7956	8277	8122	3282	1815	6742	5918	0.2424	0.9428
ferulic acid	706	403	911	940	672	342	616	379	0.6646	0.9428
ethanolamine	17432	13608	18418	10619	20542	14052	18731	10278	0.9637	0.9995
erythrose	378	166	323	97	349	102	355	142	0.8642	0.9672
erythritol	2540	2685	2040	2646	1598	2185	3783	5016	0.5344	0.9428
epsilon-caprolactam	1497	1066	714	541	1637	1409	1832	1935	0.3046	0.9428
enolpyruvate NIST	377	244	379	132	459	178	452	195	0.7297	0.9428
diglycerol	1828	1064	1826	1591	1355	757	1807	877	0.6744	0.9428
digalacturonic acid	438	156	255	118	288	75	392	454	0.4775	0.9428
deoxycholic acid	164119	329933	33803	44846	34786	35622	22009	37906	0.2910	0.9428
dehydroabietic acid	456	215	352	115	374	98	328	119	0.3570	0.9428
daidzein	440	425	217	79	278	77	274	171	0.2685	0.9428
cytosin	569	275	338	204	491	169	348	167	0.0700	0.9428
cystine	337	65	290	97	279	90	346	196	0.6084	0.9428
cysteine	3258	3672	2731	1582	1833	1347	2162	733	0.4899	0.9428
creatinine	14195	10445	11486	11703	11901	14131	9288	14928	0.7501	0.9428
conduritol-beta-epoxide	421	250	585	1018	263	37	4363	8810	0.2284	0.9428
citrulline	3574	2155	5668	4444	4292	2832	4164	1886	0.6456	0.9428
citramalic acid	855	866	384	130	551	200	514	198	0.2418	0.9428
cholic acid	31931	84557	31219	84268	1891	1259	4083	8728	0.6346	0.9428
cholesterol	1174	798	728	469	738	639	506	190	0.0501	0.9428
chenodeoxycholic acid	730	425	888	718	709	230	623	224	0.6690	0.9428
cerotinic acid	1017	1652	409	177	354	226	297	216	0.3417	0.9428
caprylic acid	968	408	947	561	1069	723	997	1008	0.9830	0.9995
capric acid	650	469	418	128	484	184	507	176	0.3830	0.9428
butyrolactam NIST	4162	4721	3415	802	2936	1967	2944	1885	0.6812	0.9428
biphenyl	2127	2463	1630	823	2483	3066	1286	682	0.6580	0.9428
beta-sitosterol	783	535	537	301	565	201	416	202	0.1805	0.9428
beta-glutamic acid	776	699	693	639	517	405	443	208	0.5731	0.9428

beta-gentiobiose	5991	9903	5155	6236	3166	2426	3854	3282	0.5632	0.9428
beta-alanine	34778	47846	22654	28008	54389	94508	21237	24547	0.5354	0.9428
benzoic acid	8387	2562	7028	1035	8047	2349	6345	2739	0.2883	0.9428
azelaic acid	501	454	488	295	535	410	393	218	0.8394	0.9672
aspartic acid	16537	10373	15744	10269	14692	14145	16673	11407	0.9817	0.9995
asparagine	2368	2318	1827	1424	1916	1462	1572	1186	0.8009	0.9635
arachidic acid	1773	879	1186	375	1407	515	1197	576	0.1503	0.9428
aminomalonate	1318	804	1170	595	1013	716	1865	1246	0.1207	0.9428
alpha-ketoglutarate	476	539	292	146	350	203	223	73	0.3295	0.9428
alpha-aminoadipic acid	1273	888	1246	451	1294	649	1154	531	0.9712	0.9995
allantoic acid	1876	3859	784	621	559	385	1213	1025	0.5414	0.9428
alanine-alanine	8517	7724	9126	9035	4813	6730	5268	3430	0.5028	0.9428
alanine	352929	84265	291792	70039	422427	245166	454471	246170	0.3495	0.9428
adipic acid	1696	2449	827	307	1001	684	778	291	0.4036	0.9428
adenosine	714	410	698	613	647	454	440	243	0.2198	0.9428
adenine	2281	1468	3072	3106	2269	951	2320	1458	0.7247	0.9428
7-methylguanine NIST	812	751	506	222	522	382	392	184	0.2435	0.9428
6-deoxyglucose	48154	50420	23255	27963	19935	18748	39469	30311	0.2008	0.9428
5-aminovaleric acid	769974	505516	1022742	504324	1134638	928791	1081002	580587	0.6725	0.9428
5,6-dihydrouracil	705	443	584	346	490	212	441	144	0.4308	0.9428
4-hydroxyphenylacetic acid	37417	49403	62123	101325	30578	19077	18204	7958	0.5397	0.9428
4-hydroxybutyric acid	866	331	742	512	1024	389	946	528	0.4519	0.9428
4-hydroxybenzoate	9131	9318	8962	7552	9795	5435	8409	5044	0.9721	0.9995
4-aminobutyric acid	1455	1544	6069	13293	1699	1463	1492	826	0.4708	0.9428
3-ureidopropionate	5123	4049	3510	1817	3860	3349	4523	4006	0.7435	0.9428
3-phenyllactic acid	868	710	842	469	1217	1215	1259	1066	0.6946	0.9428
3-hydroxypalmitic acid	1710	1576	1255	1034	1582	1317	1166	916	0.5965	0.9428
3-hydroxybutyric acid	12107	18128	5133	8279	10868	15203	4324	3805	0.3975	0.9428
3-hydroxy-3-methylglutaric acid	263	87	266	86	286	86	278	202	0.9835	0.9995
3-aminoisobutyric acid	6885	4505	7525	6858	8756	6704	5248	2386	0.6475	0.9428
3,6-anhydro-D-galactose	2290	1987	1344	576	1434	491	1289	491	0.2351	0.9428
3,4-dihydroxyphenylacetic acid	942	863	1362	1645	1388	1648	1715	1240	0.7532	0.9428
3,4-dihydroxyhydrocinnamic acid NIST	80875	102477	90719	83496	188385	381565	138412	129853	0.6971	0.9428
3,4-dihydroxycinnamic acid	717	394	543	518	693	443	698	477	0.8629	0.9672

3,4-dihydroxybenzoic acid	3791	4287	3204	2212	2416	2130	3172	1898	0.6567	0.9428
3-(4-hydroxyphenyl)propionic acid	19914	30495	14388	15007	18356	15436	12209	6483	0.6398	0.9428
3-(3-hydroxyphenyl)propionic acid	35629	83410	32192	45963	32212	50420	13999	31821	0.6611	0.9428
2-ketoisocaproic acid	6918	3558	4803	2273	6495	1714	4963	2731	0.2875	0.9428
2-ketobutyric acid	2942	1877	3133	2407	3817	1878	3186	1376	0.8110	0.9635
2-hydroxyhexanoic acid	2696	3471	2461	2518	2174	3399	2818	3190	0.9612	0.9995
2-hydroxyglutaric acid	800	413	585	327	579	209	569	260	0.4764	0.9428
2-hydroxybutanoic acid	6929	6672	10915	7034	14233	22053	15368	20415	0.7472	0.9428
2-deoxytetronic acid	1726	1310	1439	1006	1104	759	2333	3164	0.4854	0.9428
2-deoxyerythritol	1480	1327	1192	1146	1316	1170	1853	1595	0.7789	0.9569
2,4-diaminobutyric acid	4459	3094	9014	5280	7869	6460	8106	5967	0.2863	0.9428
1-monopalmitin	1005	453	599	314	587	456	519	244	0.0821	0.9428
1-kestose	320	232	209	62	302	134	194	135	0.1891	0.9428
1-deoxyerythritol	1916	952	1697	899	2145	1444	2070	1226	0.8725	0.9672
1,5-anhydroglucitol	9715	25818	573	685	730	497	3231	6917	0.5051	0.9428
2,5-dihydroxypyrazine NIST	707	400	895	488	665	202	681	265	0.5950	0.9428

p-values adjusted by Benjamini and Hochberg FDR
