# Supplementary material for: Effects of metronidazole on the fecal microbiome and metabolome in healthy dogs
Source: J Vet Intern Med. 2020 Aug 28;34(5):1853–66. doi: 10.1111/jvim.15871 (PMC7517498; doi:10.1111/jvim.15871)
Supplement: Supplementary file 5 — Supplementary Data S5. List of relevant metabolites detected in fecal samples from group 2 during the hydrolyzed protein diet trial, with mean and SD for each time point. Time points were compared with 1‐way ANOVA and adjusted for multiple comparison using Benjamini and Hochberg's False Discovery Rate, and p‐ and q‐values are presented. [file JVIM-34-1853-s005.pdf]

Group 2 diet Summary Statistics for fecal metabolomics

	day 0		day 7		day 21		day 42			
**Compound name**	**mean**	**standard deviation**	**mean**	**standard deviation**	**mean**	**standard deviation**	**mean**	**standard deviation**	**p-value**	**q-value**
xylulose NIST	10403	10356	6419	7685	3526	3847	5205	6151	0.2574	0.6499
xylose	374810	495547	172332	368086	39536	56293	112648	187640	0.2411	0.6499
xylitol	619	247	329	124	402	265	603	379	0.0603	0.6499
xanthosine	406	152	289	121	287	147	268	156	0.2208	0.6499
xanthine	6625	3442	3936	2809	4110	2429	5188	4537	0.2720	0.6499
vanillic acid	693	535	2467	3664	2093	2824	2024	1596	0.5165	0.7631
valine	209387	93034	303007	150011	308825	97783	265403	154784	0.1813	0.6499
urocanic acid	881	358	680	335	709	232	694	237	0.4834	0.7430
uridine	1813	1696	548	275	630	320	668	339	0.0308	0.6499
uric acid	4992	6776	4022	3381	6843	6040	5580	8022	0.8146	0.9030
urea	1753	1614	1342	518	1749	583	2094	996	0.5526	0.7649
uracil	56172	46032	35585	13195	45647	24114	51502	41406	0.4693	0.7430
UDP-glucuronic acid	1193	942	611	378	556	314	789	509	0.1244	0.6499
tyrosine	106304	72672	220005	148994	317573	156326	168805	103926	0.0123	0.6499
tyramine	157571	189895	146224	117616	131582	107299	79280	66106	0.6658	0.8180
tryptophan	42931	23035	43895	36004	38041	15740	45268	37534	0.9029	0.9157
trehalose	2839	5132	22043	23660	29421	28349	19913	18592	0.0621	0.6499
trans-4-hydroxyproline	7835	2561	3301	2882	4293	2856	6195	7192	0.2366	0.6499
thymine	15546	8009	8963	2639	13247	7676	12959	6345	0.1789	0.6499
thymidine	1857	1836	512	156	864	415	1153	920	0.0739	0.6499
threonine	16571	9617	24937	12159	22600	14792	27858	13404	0.2571	0.6499
threonic acid	235	74	221	111	195	115	223	113	0.8773	0.9075
threitol	974	544	617	713	696	471	1007	529	0.3591	0.7266
taurine	8587	7520	4626	5503	1493	818	2072	3227	0.0188	0.6499
tagatose	685	250	624	564	659	516	1195	1350	0.4872	0.7430
sucrose	162	45	175	165	219	89	254	270	0.7105	0.8393
stearic acid	101865	102706	76086	30554	87191	49262	80563	49793	0.7954	0.8943
spermidine	7587	8511	4638	3048	7830	4707	10405	9661	0.3616	0.7266
sinapinic acid	1036	1121	268	223	307	136	455	396	0.0723	0.6499

shikimic acid	1391	1032	1873	1856	1732	2373	1273	1547	0.8812	0.9075
serine	28232	14501	46474	15441	53846	29332	46571	22289	0.0754	0.6499
sebacic acid, di(2-octyl) ester NIST	247	102	335	442	281	173	650	531	0.1270	0.6499
salicylaldehyde	1787	1398	2479	2088	1525	1101	1675	1690	0.6943	0.8339
ribose	117959	83738	90374	40749	104704	105914	122939	89509	0.5222	0.7631
ribonic acid	369	89	305	277	212	132	403	309	0.2660	0.6499
ribitol	6271	12019	3075	2464	2449	1872	2495	1347	0.4728	0.7430
raffinose	308	362	284	255	1000	1253	671	931	0.3100	0.7016
pyruvic acid	5561	3469	3778	1538	3980	1625	3979	2648	0.4358	0.7349
putrescine	165399	75113	119893	163746	92455	70728	131904	153015	0.7170	0.8424
pseudo uridine	1997	862	1342	1132	1528	1224	2497	1648	0.0155	0.6499
propane-1,3-diol NIST	1854	2657	717	566	514	433	841	712	0.1735	0.6499
proline	94782	79070	110639	43951	144151	63728	99103	58700	0.3534	0.7244
piperidone	50080	42458	50723	29764	59596	62933	46201	48319	0.8515	0.9075
pipecolinic acid	11059	12591	6729	3432	21796	34328	9469	5133	0.3972	0.7349
pinitol	293	138	209	80	986	2154	613	648	0.4248	0.7349
pimelic acid	371	134	236	80	311	126	295	158	0.2593	0.6499
phosphate	3214	5557	1158	730	858	346	1012	618	0.3538	0.7244
phenylpyruvate	899	377	691	520	1000	559	822	843	0.6753	0.8203
phenylethylamine	3528	3133	6044	6108	5661	6088	6158	6748	0.7285	0.8512
phenylalanine	102068	46784	102362	40726	138250	47003	112544	58198	0.1664	0.6499
phenylacetic acid	6518	8346	13361	14850	20834	17899	22143	30356	0.3785	0.7331
phenol	2543	1496	2208	1380	1582	737	2988	1189	0.1228	0.6499
pentitol	256	103	167	80	188	125	250	142	0.2442	0.6499
pentadecanoic acid	4065	3521	4411	1851	7240	5191	3589	2486	0.2065	0.6499
parabanic acid NIST	2723	2336	1313	1227	2089	1062	1218	497	0.0971	0.6499
pantothenic acid	6977	6819	2732	1839	3823	2119	2838	2280	0.0528	0.6499
palmitic acid	22725	17534	20919	9325	24388	17667	18940	10302	0.7986	0.8943
oxoproline	31977	18369	24813	10042	49643	40411	35844	23021	0.1902	0.6499
ornithine	19946	9576	10906	8272	15211	6976	10913	3490	0.1088	0.6499
oleic acid	286	102	1003	2295	1022	1164	379	382	0.5739	0.7760
octadecanol	634	525	744	1268	300	110	345	227	0.5550	0.7649
O-acetylserine	592	272	453	248	373	284	485	258	0.3729	0.7331
norvaline	12837	19485	5260	3082	5240	8080	7154	5281	0.4716	0.7430

N-methylalanine	108484	55603	132787	53396	150486	60793	103259	56972	0.3217	0.7035
nicotinic acid	20922	10789	19128	9466	19315	14223	22694	7524	0.8651	0.9075
nicotianamine	587	840	228	97	305	143	366	474	0.4119	0.7349
N-acetylputrescine	2298	1105	1397	634	3821	3314	1996	1279	0.1456	0.6499
N-acetylornithine	2066	1230	1129	280	2505	2707	1139	680	0.2167	0.6499
N-acetyl-D-mannosamine	9627	16595	6393	7575	4840	4328	5235	4767	0.4010	0.7349
N-acetyl-D-hexosamine	1065	534	717	318	947	757	775	287	0.2976	0.6881
N-acetyl-D-galactosamine	37978	64983	23527	25749	18082	17981	20763	19848	0.3684	0.7331
myristic acid	7333	5795	5458	2761	6316	2870	6912	3726	0.7090	0.8393
myo-inositol	1652	1104	1013	807	1260	1495	1245	1469	0.2577	0.6499
montanic acid	793	298	601	272	674	405	647	193	0.5466	0.7631
methyltetrahydrophenanthrenone NIST	1824	987	1421	893	1554	1323	1602	779	0.8715	0.9075
methyl O-D-galactopyranoside	1884	1525	1158	1648	846	488	1508	1863	0.5031	0.7564
methionine sulfoxide	8826	5803	9251	4565	8596	4496	7669	3509	0.8641	0.9075
methionine	19614	10317	30207	15687	30235	13566	29231	17288	0.2433	0.6499
methanolphosphate	2102	2977	685	466	592	159	975	888	0.2370	0.6499
melezitose	205	128	165	40	206	106	214	116	0.7419	0.8576
maltotriose	221	110	1551	1982	1561	1418	877	997	0.0994	0.6499
maltose	12663	22478	94457	96388	131481	126601	86010	79274	0.0557	0.6499
maltitol	450	392	254	178	407	344	323	143	0.3168	0.7021
malonic acid	280	173	220	88	467	333	296	224	0.1421	0.6499
malic acid	3367	6954	564	407	794	548	5374	12848	0.5264	0.7631
maleimide	1617	739	948	339	1724	652	2046	1979	0.2408	0.6499
maleic acid	466	486	256	97	303	188	377	408	0.5949	0.7924
lyxose	16795	19661	10487	22450	2642	4409	5774	8773	0.3397	0.7160
lyxitol	2592	1060	2013	1905	3535	3818	2664	1374	0.6178	0.8051
lysine	166368	138551	235186	103927	255340	96706	172861	131062	0.2314	0.6499
lithocholic acid	975	935	674	671	2485	2347	546	411	0.0298	0.6499
linoleic acid	309	161	524	828	250	167	350	257	0.6334	0.8058
lignoceric acid	398	241	366	139	424	208	472	283	0.7964	0.8943
levoglucosan	1734	2441	1314	2538	639	704	603	426	0.5338	0.7631
leucine	194075	87967	269522	130109	254899	109712	250321	156227	0.4187	0.7349
lactulose	2411	3724	1425	959	1514	1148	1371	1041	0.6034	0.7948
lactose	1143	2089	968	940	766	511	1175	751	0.8949	0.9119

lactitol	2071	2831	2838	1748	2656	1816	3010	1191	0.8148	0.9030
kynurenic acid	677	805	485	389	752	1012	723	548	0.8429	0.9075
isothreonic acid	609	325	431	301	345	196	464	250	0.2368	0.6499
isomaltose	366	150	454	269	541	237	488	298	0.4379	0.7349
isoleucine	142321	84821	227737	115021	262398	79949	202621	127200	0.0459	0.6499
inosine	1454	1143	668	320	504	452	749	1019	0.1276	0.6499
indole-3-lactate	22460	21302	4915	7695	3786	7379	15006	33488	0.2682	0.6499
indole-3-acetate	2995	2893	2098	2109	2166	2046	2313	1904	0.8294	0.9075
hypoxanthine	9610	8390	3463	2812	2289	1205	6084	8490	0.0807	0.6499
hydroxylamine	65931	45521	64363	29494	52870	40429	48792	25848	0.7796	0.8916
hydroxycarbamate NIST	16999	12823	14503	7118	11218	7883	9495	5485	0.4363	0.7349
homoserine	1631	368	1222	968	939	539	1505	720	0.1258	0.6499
homocystine	646	540	354	146	427	259	451	322	0.2612	0.6499
histidine	13141	11480	17009	7808	19917	7721	11927	10055	0.2153	0.6499
hexuronic acid	2111	1941	1554	1375	1083	718	1286	1130	0.2332	0.6499
hexonic acid	721	638	1041	627	1292	1023	766	587	0.3967	0.7349
hexitol	541	281	417	169	355	192	418	286	0.0713	0.6499
heptadecanoic acid	1811	1651	1125	567	2056	1800	1464	980	0.4493	0.7373
guanosine	1001	766	734	557	467	228	838	1297	0.6063	0.7948
guanine	856	627	641	403	617	216	571	564	0.4205	0.7349
glycyl tyrosine	742	530	412	123	518	198	370	85	0.0988	0.6499
glycyl proline	7731	5947	4467	1271	5814	2742	8085	9908	0.4823	0.7430
glycolic acid	8519	3276	9681	8801	5232	3609	12117	7018	0.2177	0.6499
glycine	37789	19542	41805	14268	65623	27454	57933	50096	0.2720	0.6499
glycerol-alpha-phosphate	519	282	236	157	260	151	315	171	0.0280	0.6499
glycerol-3-galactoside	850	764	4864	8539	1883	1043	3104	3735	0.4135	0.7349
glycerol	32536	19884	65181	106956	32888	22232	38835	27747	0.6423	0.8064
glyceric acid	6705	4037	4410	3216	5622	5034	6157	7463	0.7594	0.8731
glutaric acid	685	426	312	168	444	176	816	652	0.1020	0.6499
glutamine	10452	8382	15458	8825	20845	14731	15116	9733	0.1261	0.6499
glutamic acid	79874	46221	91697	50417	106638	31534	92495	38198	0.5690	0.7743
glucose-1-phosphate	1454	812	919	777	640	434	685	248	0.0089	0.6499
glucose	57362	70250	190345	106282	138103	26150	171428	113653	0.0338	0.6499
glucoheptulose	1082	1433	350	192	293	180	609	548	0.1044	0.6499

galacturonic acid	2211	3576	553	683	308	157	561	735	0.1608	0.6499
galactitol	1702	2630	647	262	728	400	688	485	0.3754	0.7331
galactinol	411	201	241	117	320	181	435	487	0.2901	0.6779
fumaric acid	3446	3393	2623	1615	2884	1550	7696	10606	0.2114	0.6499
fucose	149618	286349	67856	55932	51737	43921	76084	73946	0.4012	0.7349
fructose	4191	2959	6695	6346	4848	2815	6213	4210	0.6451	0.8064
ferulic acid	750	719	537	711	311	261	554	521	0.3441	0.7182
ethanolamine	37798	45490	18075	11231	29374	37560	23313	19510	0.3059	0.6997
erythrose	335	139	301	177	268	132	365	190	0.6706	0.8192
erythritol	2612	4242	2372	2606	1575	2347	4069	5456	0.5355	0.7631
epsilon-caprolactam	1849	2045	933	568	604	289	949	1042	0.1911	0.6499
enolpyruvate NIST	437	210	342	221	358	128	343	182	0.7401	0.8576
diglycerol	2214	1615	558	482	905	928	1099	738	0.0228	0.6499
digalacturonic acid	586	893	203	90	358	400	291	96	0.4856	0.7430
deoxycholic acid	89498	118569	61274	39907	130605	133915	39469	32724	0.2196	0.6499
dehydroabietic acid	403	252	347	116	405	111	402	176	0.8911	0.9119
daidzein	415	549	547	270	1034	861	688	504	0.2086	0.6499
cytosin	552	290	282	94	372	198	682	876	0.4015	0.7349
cystine	330	136	304	215	380	252	331	206	0.8728	0.9075
cysteine	2123	1929	1327	839	1266	808	1758	1043	0.5371	0.7631
creatinine	8306	9168	7391	8916	9834	8212	13492	22622	0.6328	0.8058
conduritol-beta-epoxide	282	111	354	483	654	780	452	450	0.5430	0.7631
citrulline	3062	1302	4743	2430	6239	3664	6729	2159	0.0302	0.6499
citramalic acid	1053	1158	535	171	725	812	849	562	0.5413	0.7631
cholic acid	5077	8723	548	299	1690	2668	1132	1114	0.2047	0.6499
cholesterol	634	365	604	481	1051	966	1190	1819	0.6329	0.8058
chenodeoxycholic acid	971	887	495	278	487	187	519	207	0.1978	0.6499
cerotinic acid	430	137	287	184	369	284	253	116	0.2247	0.6499
caprylic acid	1124	841	1153	556	1094	604	1021	550	0.9834	0.9840
capric acid	569	402	586	341	485	148	541	144	0.8822	0.9075
butyrolactam NIST	2091	644	1673	762	1730	973	1890	1194	0.8271	0.9075
biphenyl	1071	491	547	187	1614	1988	810	559	0.2801	0.6617
beta-sitosterol	621	333	660	285	727	374	927	909	0.6585	0.8180
beta-glutamic acid	479	363	477	492	1502	1371	1708	2988	0.2661	0.6499

beta-gentiobiose	5912	5509	3741	2446	5164	3627	3622	2284	0.4227	0.7349
beta-alanine	7746	12373	35143	41727	21556	18795	36300	54933	0.3239	0.7035
benzoic acid	7853	4660	7676	4385	7164	3169	8755	3620	0.8760	0.9075
azelaic acid	431	345	317	115	418	186	379	147	0.6279	0.8058
aspartic acid	24695	31531	23278	12337	32582	14818	34698	23546	0.5393	0.7631
asparagine	1218	683	1312	1247	1399	835	1368	805	0.9840	0.9840
arachidic acid	1276	627	923	257	1023	651	985	519	0.4745	0.7430
aminomalonate	1576	1534	1515	798	1781	835	1415	875	0.8627	0.9075
alpha-ketoglutarate	680	1138	323	92	892	954	381	301	0.4444	0.7349
alpha-aminoadipic acid	1350	1038	434	365	2474	3919	596	315	0.2281	0.6499
allantoic acid	1204	2315	2271	3923	2272	1481	2658	3239	0.5586	0.7649
alanine-alanine	9797	8322	3530	3670	5146	3777	4697	4166	0.0446	0.6499
alanine	449669	155430	410922	173253	485246	167465	485254	193739	0.7898	0.8943
adipic acid	618	261	416	257	437	244	514	322	0.4998	0.7564
adenosine	997	862	575	663	668	839	587	578	0.5934	0.7924
adenine	1684	1884	806	701	930	793	1720	2158	0.4715	0.7430
7-methylguanine NIST	816	622	395	249	823	505	525	290	0.1089	0.6499
6-deoxyglucose	25883	30319	42481	34294	30986	40649	50020	42090	0.5369	0.7631
5-aminovaleric acid	830256	550182	702091	449862	434318	244932	875883	738300	0.1704	0.6499
5,6-dihydrouracil	532	336	234	98	353	192	420	351	0.1493	0.6499
4-hydroxyphenylacetic acid	14312	9568	6006	4500	10753	7237	12470	19582	0.4801	0.7430
4-hydroxybutyric acid	1457	1200	773	549	637	486	887	688	0.1543	0.6499
4-hydroxybenzoate	6067	5820	2513	2679	1510	972	3613	4684	0.1755	0.6499
4-aminobutyric acid	1342	673	583	393	523	428	883	1174	0.1348	0.6499
3-ureidopropionate	5382	4621	2892	1791	6311	5810	5226	4310	0.2597	0.6499
3-phenyllactic acid	1683	1225	1137	1460	1446	1680	1535	2137	0.8689	0.9075
3-hydroxypalmitic acid	863	579	488	195	1282	1545	978	1456	0.5971	0.7924
3-hydroxybutyric acid	5367	2697	4374	2892	3251	2271	6453	4685	0.1176	0.6499
3-hydroxy-3-methylglutaric acid	261	104	156	59	224	90	193	99	0.0869	0.6499
3-aminoisobutyric acid	3434	2595	5851	4179	4770	3428	5193	4166	0.4287	0.7349
3,6-anhydro-D-galactose	1969	1841	884	604	1011	462	1519	915	0.2200	0.6499
3,4-dihydroxyphenylacetic acid	820	1034	275	238	266	134	419	397	0.2285	0.6499
3,4-dihydroxyhydrocinnamic acid NIST	55460	47631	36823	56220	19222	17436	38117	88143	0.6794	0.8206
3,4-dihydroxycinnamic acid	846	486	306	165	393	244	391	201	0.0069	0.6499

3,4-dihydroxybenzoic acid	3191	2169	2075	2845	1262	1447	1864	3110	0.3344	0.7160
3-(4-hydroxyphenyl)propionic acid	14273	11592	13865	8007	12403	12696	13712	12283	0.9646	0.9736
3-(3-hydroxyphenyl)propionic acid	6531	15257	6521	10852	3948	6364	5701	13299	0.7098	0.8393
2-ketoisocaproic acid	7252	4054	9485	3801	13166	5757	6951	4325	0.0480	0.6499
2-ketobutyric acid	2707	1169	3017	2225	4657	2014	3077	1333	0.1879	0.6499
2-hydroxyhexanoic acid	7065	11702	1342	864	1886	1520	2282	2039	0.2368	0.6499
2-hydroxyglutaric acid	800	359	426	147	688	280	1214	1705	0.3133	0.7016
2-hydroxybutanoic acid	31575	40026	8018	7703	10096	8614	5556	3147	0.0719	0.6499
2-deoxytetronic acid	1855	1437	1242	1200	693	549	2236	2838	0.3376	0.7160
2-deoxyerythritol	1339	1204	2759	2863	2803	4080	4176	7157	0.6658	0.8180
2,4-diaminobutyric acid	4915	3257	4183	1852	4662	1937	4333	2498	0.8544	0.9075
1-monopalmitin	998	830	752	645	632	220	808	344	0.6431	0.8064
1-kestose	330	358	384	275	909	1189	525	707	0.4377	0.7349
1-deoxyerythritol	1543	918	5301	8326	2778	3271	2340	1362	0.4430	0.7349
1,5-anhydroglucitol	939	646	522	549	358	241	967	1396	0.4071	0.7349
2,5-dihydroxypyrazine NIST	687	248	351	195	446	213	657	543	0.1442	0.6499

p-values adjusted by Benjamini and Hochberg FDR
