# Supplementary material for: Effects of metronidazole on the fecal microbiome and metabolome in healthy dogs
Source: J Vet Intern Med. 2020 Aug 28;34(5):1853–66. doi: 10.1111/jvim.15871 (PMC7517498; doi:10.1111/jvim.15871)
Supplement: Supplementary file 6 — Supplementary Data S6. List of relevant metabolites detected in fecal samples from groups 2 and 3 during the metronidazole trial, with mean and SD for each time point. Time points were compared with 1‐way ANOVA and adjusted for multiple comparison using Benjamini and Hochberg's False Discovery Rate, and p‐ and q‐values are presented. [file JVIM-34-1853-s006.pdf]

Group 2 metronidazole and Group 3 Combined Summary Statistics for Fecal Metabolomics

	day 0		day 7		day 14		day 28		day 42			
Compound name	mean	standard deviation	mean	standard deviation	mean	standard deviation	mean	standard deviation	mean	standard deviation	p-value	q-value
xylulose NIST	8541.6875^{a}	8317	1550.1875^{b}	2581	4735.6875^{ab}	8224	4656.5625^{ab}	5830	6538.4375^{ab}	6969	0.011	0.038
xylose	229577	310773	35818	75325	103670	250489	178136	275134	226939	360140	0.113	0.206
xylitol	766	590	672	399	706	595	1355	1911	784	639	0.217	0.331
xanthosine	344	196	197	88	281	175	274	142	247	88	0.025	0.073
xanthine	6347.5^{a}	4648	1797.375^{b}	1237	2014.875^{b}	1372	4406.25^{ab}	3391	3260.375^{b}	2246	0.000	0.001
vanillic acid	1714.0625^{a}	1652	1236.6875^{ab}	747	994.25^{ab}	418	654.8125^{b}	653	792.75^{b}	653	0.003	0.016
valine	203530	135145	239069	106644	194444	116280	203803	119282	256245	141390	0.484	0.587
urocanic acid	896	489	692	566	631	474	798	330	648	231	0.424	0.533
uridine	913	401	824	472	1228	909	1213	1083	666	343	0.066	0.145
uric acid	3812	5923	854	893	782	452	1420	1198	2945	7362	0.082	0.164
urea	3159	1997	16136	31846	34048	62790	2645	1952	2574	998	0.019	0.059
uracil	57787.875^{a}	41414	7832^{c}	4369	7433.0625^{c}	4030	21215.9375^{bc}	17651	31674.0625^{b}	23373	0.000	0.000
UDP-glucuronic acid	1031.5^{b}	611	3827.0625^{a}	2634	4590.3125^{a}	5077	864.6875^{b}	584	720.5^{b}	464	0.000	0.000
tyrosine	160967.875^{a}	85245	51785.25^{c}	66754	66345.8125^{bc}	50595	160326.375^{a}	95390	138835.0625^{ab}	83402	0.000	0.000
tyramine	62290.1875^{c}	54175	318359.0625^{a}	175503	197054.1875^{ab}	160240	122713^{bc}	130050	150332.9375^{bc}	172582	0.000	0.000
tryptophan	65880	53622	37921	20658	38382	16010	45871	37736	35470	20779	0.071	0.151
trehalose	10731	15863	2868	5939	2414	3018	6209	8585	5331	5799	0.066	0.145
trans-4-hydroxyproline	11066.6875^{b}	11069	15549.8125^{b}	9819	36370.75^{a}	37558	8789.375^{b}	5576	12934.8125^{b}	8497	0.000	0.002
thymine	16149.5^{a}	9655	3448.125^{c}	1698	3806.375^{bc}	1865	7246.4375^{bc}	4497	9123.625^{b}	6095	0.000	0.000
thymidine	1425^{a}	788	823.1875^{b}	337	1599.1875^{a}	889	1106.75^{ab}	707	769.4375^{b}	361	0.001	0.003
threonine	25098	11885	25801	16070	29829	14183	33005	18858	32961	16972	0.424	0.533
threonic acid	373	317	262	141	368	319	312	188	266	134	0.458	0.566
threitol	1539	1377	1233	560	1082	580	1310	867	760	666	0.063	0.142
taurine	3453	3127	3919	4706	8385	11955	7258	6240	4249	4752	0.174	0.285
tagatose	1236.0625^{b}	1021	2765.375^{a}	2141	2367.125^{ab}	1662	1970.875^{ab}	2086	1615.875^{ab}	1743	0.013	0.044
sucrose	283	236	198	100	338	336	286	315	396	522	0.580	0.652
stearic acid	113620	71337	64136	34115	94917	49252	98556	60255	73016	27374	0.027	0.076

spermidine	8153.5625^{a}	7206	1682.75^{c}	1032	2182.625^{bc}	1772	3848.8125^{bc}	2260	5251.875^{ab}	4080	0.000	0.000
sinapinic acid	465	320	605	375	551	335	446	256	495	405	0.531	0.627
shikimic acid	1487	1278	1537	2232	1382	982	1959	1876	1310	1164	0.775	0.801
serine	39237	20263	25343	15302	29512	13706	43055	31858	41197	23425	0.080	0.164
sebacic acid, di(2-octyl) ester NIST	534	415	232	109	467	366	367	260	420	300	0.054	0.128
salicylaldehyde	2303.8125^{a}	2516	692.1875^{b}	518	848.75^{b}	564	736.5^{b}	501	1221.75^{ab}	831	0.004	0.017
ribose	100404.4375^{a}	72795	20023^{c}	16342	19109.75^{c}	10496	52107.625^{bc}	37137	64904.125^{ab}	62519	0.000	0.000
ribonic acid	714	852	689	303	1009	551	735	848	435	346	0.155	0.260
ribitol	2287	1203	1565	955	2245	1966	2127	2450	3104	4745	0.583	0.652
raffinose	496	666	353	299	441	312	461	635	312	234	0.725	0.777
pyruvic acid	6480.625^{b}	7110	31822.125^{a}	34001	17059^{ab}	13474	3831.875^{b}	1935	4123.125^{b}	3035	0.000	0.000
putrescine	191032.25^{bc}	250791	477699.4375^{a}	391211	392923.8125^{ab}	346803	163580.875^{c}	352535	266151.0625^{abc}	395031	0.000	0.002
pseudo uridine	2730.9375^{a}	2466	973.0625^{b}	1095	1089.25^{b}	1068	1642.5625^{ab}	938	1466.3125^{ab}	1336	0.009	0.034
propane-1,3-diol NIST	1058.75^{b}	1431	2721.4375^{ab}	2550	4202.4375^{a}	4979	700.1875^{b}	639	706.125^{b}	812	0.001	0.003
proline	79526	49313	90002	96782	81795	84745	83533	62247	97278	75309	0.962	0.962
piperidone	35832	41165	13421	18265	18192	21969	19088	19580	32413	45254	0.102	0.189
pipecolinic acid	11634	8290	7531	13711	4970	3784	8189	6027	16367	18183	0.028	0.076
pinitol	483	479	109117	190602	107138	268696	91263	243341	41422	144983	0.231	0.347
pimelic acid	414	229	294	124	405	277	411	150	371	153	0.212	0.326
phosphate	2534	4174	4195	5051	3528	4499	1207	1303	1109	773	0.059	0.136
phenylpyruvate	1190.8125^{b}	1374	4293.5^{a}	2876	4420.9375^{a}	4261	1000.5625^{b}	705	1205.25^{b}	1274	0.000	0.000
phenylethylamine	4022	5153	7558	9006	4986	10922	6769	8300	5037	6002	0.726	0.777
phenylalanine	95895.125^{ab}	50717	74482.5625^{ab}	29530	61979.0625^{b}	27744	94790^{ab}	50999	109223.5625^{a}	47472	0.009	0.034
phenylacetic acid	15852	22487	7757	10428	9838	9148	13172	16119	12633	11670	0.514	0.611
phenol	3904.4375^{b}	4133	6400.4375^{ab}	5202	9685.5625^{a}	9943	2118.8125^{b}	1181	2244.0625^{b}	1180	0.000	0.002
pentitol	389	269	297	229	565	743	307	159	273	176	0.088	0.171
pentadecanoic acid	4062	3072	3934	3830	3664	5667	4395	4591	2963	2600	0.843	0.859
parabanic acid NIST	2753	2760	1503	953	2019	2105	1465	1487	1340	797	0.151	0.258
pantothenic acid	3565.1875^{a}	2655	1993.125^{ab}	2246	1670.25^{b}	1370	1638.8125^{b}	1477	1445.5625^{b}	1595	0.013	0.044
palmitic acid	25811	15088	18102	12217	26800	21615	23204	17352	19140	7411	0.330	0.441
oxoproline	40619.4375^{c}	18830	372966.875^{ab}	348944	405774.4375^{a}	288786	139633.75^{c}	169893	166199.8125^{bc}	268255	0.000	0.000
ornithine	25482.125^{ab}	24898	47722.9375^{a}	42097	39768.75^{ab}	22250	21138.1875^{b}	17298	18473.5625^{b}	18035	0.004	0.018

oleic acid	392	295	277	435	253	181	611	906	219	132	0.098	0.183
octadecanol	376	222	332	164	973	2304	444	231	378	171	0.408	0.519
O-acetylserine	546	280	395	170	446	220	899	1710	386	315	0.345	0.458
norvaline	6164	4434	3572	7090	4088	6699	4191	4897	6552	7905	0.537	0.631
N-methylalanine	79846.625[a]	48651	35873.5[b]	25254	36079.3125[b]	30426	63103.4375[ab]	53877	63926.3125[ab]	48511	0.002	0.010
nicotinic acid	24878.5[a]	10806	8141.6875[c]	4695	9344.5625[c]	4550	17289.5[b]	6088	18709.6875[ab]	8619	0.000	0.000
nicotianamine	560	514	1094	1487	1083	1955	649	824	372	284	0.253	0.365
N-acetylputrescine	2518	1183	6420	6646	5079	4962	2979	4841	4169	7336	0.084	0.165
N-acetylornithine	1978	1242	5124	15717	1267	621	2258	2726	1416	1332	0.555	0.641
N-acetyl-D-mannosamine	4094	3833	5130	4794	8237	14287	4654	4929	2686	3103	0.296	0.410
N-acetyl-D-hexosamine	997	450	548	236	933	663	944	463	884	381	0.027	0.076
N-acetyl-D-galactosamine	15803	15522	6369	4780	7187	6807	14872	23858	10728	11834	0.051	0.124
myristic acid	10277	6513	7765	6735	11951	9490	11289	10989	7549	4857	0.183	0.293
myo-inositol	1340	1061	12131	19568	29307	60248	5809	7466	5749	12415	0.052	0.124
montanic acid	824	368	576	376	861	662	767	348	642	349	0.131	0.230
methyltetrahydrophenanthrenone NIST	2128	1086	1721	1159	1897	1320	1834	1090	1424	689	0.359	0.471
methyl O-D-galactopyranoside	1399.4375[b]	1342	2824.25[ab]	2086	3924.9375[a]	3238	1697.1875[b]	1484	1457.9375[b]	1521	0.000	0.003
methionine sulfoxide	10237	6967	6213	3033	7099	8836	10527	6417	11554	6893	0.093	0.178
methionine	23819	16481	19115	11685	18987	8921	30179	18460	28281	16804	0.076	0.159
methanolphosphate	1256	1357	1258	1204	1123	1166	734	556	709	567	0.302	0.412
melezitose	269[b]	149	277.3125[ab]	244	602.75[a]	698	244.6875[b]	152	206.3125[b]	86	0.010	0.035
maltotriose	599	753	191	103	267	196	277	148	346	343	0.029	0.079
maltose	45905	68234	11174	15937	7806	13292	20093	34788	20374	25700	0.026	0.075
maltitol	343	133	466	493	339	308	419	229	395	681	0.871	0.884
malonic acid	348	200	281	150	345	228	370	468	265	156	0.725	0.777
malic acid	3821	9048	2100	2418	2376	4215	1195	806	1194	729	0.480	0.587
maleimide	2732	2085	2971	2816	4483	7363	2196	1893	2165	1064	0.373	0.480
maleic acid	370	316	255	137	333	235	301	178	288	179	0.596	0.661
lyxose	10082.8125[a]	11590	1725.0625[a]	2271	1300.5625[a]	804	9655.1875[a]	16293	11134.5625[a]	15227	0.013	0.044
lyxitol	3913	3711	2501	2601	2704	2478	3927	3091	2885	2982	0.359	0.471
lysine	156312.5[a]	103522	117590.3125[ab]	128641	65074.3125[b]	64207	179787.1875[a]	103088	127550.5[ab]	84978	0.008	0.031
lithocholic acid	577.375[a]	345	186.625[b]	91	272.3125[b]	194	410.8125[ab]	423	306.875[b]	228	0.001	0.005
linoleic acid	394.5625[a]	241	210.0625[b]	74	279.3125[ab]	155	282.75[ab]	180	253.875[ab]	93	0.012	0.041

lignoceric acid	493	263	474	673	394	226	607	567	385	255	0.624	0.688
levoglucosan	644	363	1479	2910	2212	4697	650	717	463	215	0.152	0.258
leucine	199269	132624	215966	87092	182929	89218	203699	109859	243428	119277	0.507	0.606
lactulose	2188	2264	1976	1813	2266	3083	1713	1250	1126	948	0.403	0.516
lactose	1072	672	619	678	592	411	846	724	559	431	0.084	0.165
lactitol	2682	1473	834	1262	893	1294	1786	2079	2461	3287	0.016	0.052
kynurenic acid	693	449	5761	8997	6466	9775	4616	14397	448	248	0.083	0.165
isothreonic acid	905	703	769	270	928	389	631	375	610	495	0.118	0.213
isomaltose	542	350	631	475	1076	2025	682	295	400	314	0.364	0.474
isoleucine	153834	113646	158040	63566	147920	84438	158726	104372	186479	101165	0.757	0.794
inosine	1347.875[a]	1043	315.5[b]	189	397.4375[b]	282	601.5625[b]	279	415.3125[b]	357	0.000	0.000
indole-3-lactate	20375	28358	15189	8287	15064	13098	12035	13566	8062	12044	0.184	0.293
indole-3-acetate	3039	2696	5996	9280	6764	10565	4335	5180	5095	12929	0.735	0.780
hypoxanthine	9537.5625[a]	8712	3564.125[b]	2560	3940.125[b]	3491	7728.625[ab]	6237	5494.8125[ab]	4796	0.006	0.025
hydroxylamine	86825	74800	60521	37071	56747	52453	58549	50197	47521	34623	0.262	0.373
hydroxycarbamate NIST	20164	20205	12909	9180	13146	13402	11757	10291	10771	8253	0.243	0.353
homoserine	1771.75[a]	804	545.875[c]	324	572.4375[c]	285	892.8125[bc]	560	1112.125[b]	757	0.000	0.000
homocystine	669	545	650	357	780	419	679	248	490	244	0.235	0.350
histidine	11623.9375[b]	7551	22240.4375[a]	16402	14351.6875[ab]	13100	14154.75[ab]	7562	9578.75[b]	7017	0.005	0.022
hexuronic acid	1112.875[b]	881	3121[a]	2319	3452.75[a]	3685	1057.1875[b]	821	800.9375[b]	591	0.000	0.000
hexonic acid	729	438	677	578	422	242	665	600	497	287	0.261	0.373
hexitol	441	240	676	432	861	666	708	486	466	294	0.032	0.085
heptadecanoic acid	1887	1501	1285	1032	1816	1507	1457	843	1094	437	0.188	0.298
guanosine	726.3125[a]	934	203.9375[b]	91	267.875[b]	138	415.5[ab]	324	256.1875[b]	154	0.012	0.043
guanine	837	686	520	262	760	906	927	1430	544	505	0.573	0.650
glycyl tyrosine	647	409	597	544	592	408	952	1006	513	143	0.236	0.350
glycyl proline	10591.8125[ab]	8808	16924.875[ab]	14709	18324.8125[a]	16334	7456.5625[b]	5784	9986.375[ab]	9264	0.009	0.034
glycolic acid	13211.8125[a]	7248	4875.875[c]	3605	4152[c]	2789	9739.75[ab]	5893	8228.8125[bc]	3157	0.000	0.000
glycine	52497.375[b]	35985	128452[ab]	106348	185798.5[a]	134158	56720.6875[b]	40001	51449.25[b]	56667	0.000	0.000
glycerol-alpha-phosphate	790	1005	695	519	905	838	621	358	376	260	0.161	0.266
glycerol-3-galactoside	1968	2831	1052	781	1185	858	1579	1160	972	1036	0.323	0.436
glycerol	39382.1875[a]	23643	19306.25[b]	18711	24400.0625[ab]	17442	26629.5625[ab]	13086	33488[ab]	15363	0.008	0.031
glyceric acid	7435	6260	3556	1831	3600	1899	7175	6845	5479	3330	0.021	0.064

glutaric acid	1306	1842	262	120	637	825	609	415	760	489	0.051	0.124
glutamine	13648	8550	10833	9447	11201	11336	17943	11630	17774	12616	0.180	0.292
glutamic acid	85786.5625[b]	33617	407191.625[a]	303623	382784.25[a]	255292	165708.6875[b]	137244	153938.5[b]	161775	0.000	0.000
glucose-1-phosphate	1011.875[bc]	482	1676.6875[ab]	945	1729.8125[a]	965	1219[abc]	915	767.5625[c]	436	0.001	0.005
glucose	107749.6875[a]	102546	22288.4375[b]	20613	29537.6875[b]	35727	69522.125[ab]	87771	63142.625[ab]	67847	0.000	0.003
glucoheptulose	840	844	739	431	903	695	540	238	426	224	0.035	0.092
galacturonic acid	1070	1492	654	1122	443	437	1143	2180	381	424	0.300	0.412
galactitol	889	706	742	670	557	495	707	531	838	1324	0.767	0.801
galactinol	651	638	3195	6653	1550	2931	652	374	1621	4978	0.292	0.407
fumaric acid	6162	7669	3758	2165	3405	2602	4084	4060	4648	7929	0.567	0.648
fucose	59954	55053	35563	23698	44814	36627	47728	55994	43636	49757	0.455	0.566
fructose	5269	3169	3752	5614	10015	22833	5300	3891	5725	5209	0.585	0.652
ferulic acid	688	574	956	896	807	592	455	331	541	355	0.067	0.145
ethanolamine	16009	15550	22000	23319	17283	15301	13134	11608	10638	10788	0.098	0.183
erythrose	425	210	408	262	377	190	309	194	298	167	0.241	0.353
erythritol	11052	18657	7248	7376	8577	10342	8923	13765	4273	11519	0.229	0.347
epsilon-caprolactam	1506	1259	1256	1236	1267	866	1799	2306	847	405	0.329	0.441
enolpyruvate NIST	456	319	490	248	607	432	466	390	335	144	0.196	0.305
diglycerol	2315	2369	1908	1271	2478	1816	1898	1311	1947	1767	0.804	0.823
digalacturonic acid	361	181	235	140	346	184	308	215	383	419	0.453	0.566
deoxycholic acid	35764.375[a]	37358	4284.4375[b]	4229	4627.8125[b]	8551	18725.625[ab]	23399	14134.4375[b]	17878	0.000	0.002
dehydroabietic acid	585	312	351	178	495	260	444	239	376	144	0.024	0.072
daidzein	639	619	493	516	592	408	410	292	493	472	0.542	0.631
cytosin	593	636	516	498	439	405	417	199	388	161	0.574	0.650
cystine	386	231	1500	2971	684	830	377	160	271	112	0.070	0.150
cysteine	2269.875[b]	1856	5841.25[a]	3594	5697.625[a]	3292	3490.875[ab]	2341	2710[b]	1831	0.000	0.001
creatinine	14197	17685	6794	5753	12472	11519	14924	17546	8578	8328	0.303	0.412
conduritol-beta-epoxide	682	1115	24047	38509	17519	51978	12533	36451	7311	23122	0.190	0.298
citrulline	4898	2597	10247	21960	11486	33807	5996	3529	5657	2929	0.710	0.771
citramalic acid	903.5625[a]	605	327.5625[c]	155	429.1875[bc]	237	697.4375[ab]	499	602.6875[abc]	298	0.000	0.002
cholic acid	2186	4152	57470	80315	100432	144778	33577	72157	22448	59179	0.025	0.073
cholesterol	1041	1302	518	222	760	570	607	313	511	294	0.120	0.215
chenodeoxycholic acid	899	982	1279	1497	1684	1541	1413	1614	929	1453	0.504	0.605

cerotinic acid	438	343	308	144	376	228	443	402	247	170	0.207	0.320
caprylic acid	1985	2032	962	682	1051	727	1149	1090	1144	651	0.080	0.164
capric acid	659	275	365	226	450	236	547	477	457	155	0.035	0.092
butyrolactam NIST	2477	1579	4905	7689	3496	3440	1962	1192	2664	1609	0.276	0.387
biphenyl	1391	1029	1224	1101	1378	1104	1343	853	1230	975	0.955	0.960
beta-sitosterol	828.1875[a]	697	399.625[b]	175	527.1875[ab]	307	545.4375[ab]	296	418.0625[b]	233	0.008	0.033
beta-glutamic acid	1324	2174	1796	3094	2810	3951	2336	3356	1325	2034	0.563	0.647
beta-gentiobiose	3135	1874	13071	19766	9594	18831	5744	5450	10568	32466	0.543	0.631
beta-alanine	22486.4375[a]	40469	797.5[b]	514	2363.1875[ab]	3747	15879.375[ab]	13479	19784.5625[ab]	16776	0.009	0.034
benzoic acid	11125	5385	18419	34906	14674	15408	10423	12870	8369	4699	0.467	0.574
azelaic acid	379	178	247	155	389	237	393	223	429	283	0.145	0.249
aspartic acid	35714	22523	41111	35794	32068	19686	37545	30310	30253	13692	0.774	0.801
asparagine	1776	1042	1938	1865	2714	4099	2020	1430	2197	1653	0.737	0.780
arachidic acid	1651	1369	2269	2318	1660	809	1403	729	1032	394	0.096	0.182
aminomalonate	1607.25[b]	739	4219.4375[a]	1971	5217.4375[a]	3464	1721.25[b]	1409	1256.75[b]	727	0.000	0.000
alpha-ketoglutarate	396.875[b]	269	1014.3125[ab]	1251	1100.9375[a]	965	400.1875[b]	213	387.0625[b]	310	0.003	0.016
alpha-aminoadipic acid	1055	633	632	395	797	557	895	457	963	679	0.056	0.132
allantoic acid	2417	2985	857	1160	1248	2014	1810	2534	1221	1149	0.160	0.266
alanine-alanine	10852	13289	11819	11167	10422	7210	12441	13747	5953	6204	0.271	0.383
alanine	446604	170827	517168	261975	501912	232092	316798	156906	399535	208288	0.045	0.112
adipic acid	799[a]	482	423.3125[b]	157	563.5625[ab]	300	627.6875[ab]	296	533.75[ab]	183	0.005	0.022
adenosine	755	572	358	215	452	237	411	428	488	639	0.127	0.226
adenine	2407	2114	2017	1268	2545	2164	2085	1884	2293	2892	0.931	0.940
7-methylguanine NIST	678	570	401	234	475	199	538	269	403	178	0.074	0.155
6-deoxyglucose	43145	38229	11211	14813	7400	7022	40069	60200	29020	65836	0.042	0.106
5-aminovaleric acid	935694.9375[a]	679169	297542.1875[b]	286551	375092.375[b]	264553	1169792.4375[a]	830539	965808.125[a]	678334	0.000	0.000
5,6-dihydrouracil	920	1257	494	291	565	309	459	253	384	291	0.139	0.243
4-hydroxyphenylacetic acid	18103	25782	23888	41015	13535	17642	24172	30128	18692	21346	0.795	0.818
4-hydroxybutyric acid	1171.0625[ab]	694	2172.1875[a]	1330	2309.6875[a]	2217	942.0625[b]	487	1425.5625[ab]	1765	0.005	0.022
4-hydroxybenzoate	4826.25[a]	4373	851.9375[b]	471	1156.125[b]	955	3187.6875[ab]	1929	4138.8125[a]	4205	0.000	0.000
4-aminobutyric acid	1331	1600	21045	50510	10880	29728	2928	5861	4618	8188	0.237	0.350
3-ureidopropionate	4504.125[a]	3396	978.5625[b]	736	1120.75[b]	460	2378.625[b]	1884	2284.9375[b]	2612	0.000	0.001
3-phenyllactic acid	2536.1875[b]	3928	12055.375[a]	9507	11911.8125[a]	12604	2077.8125[b]	1794	2619.875[b]	3358	0.000	0.000

3-hydroxypalmitic acid	1065.5[a]	1206	330.5625[b]	415	365.9375[b]	354	707.75[ab]	623	533.75[ab]	655	0.003	0.016
3-hydroxybutyric acid	9562	15514	2786	2639	1853	1677	4067	4363	6446	8516	0.062	0.140
3-hydroxy-3-methylglutaric acid	258	165	246	197	293	214	456	455	227	126	0.109	0.201
3-aminoisobutyric acid	4872	3215	2114	1654	4338	7209	4999	3119	6377	3196	0.061	0.139
3,6-anhydro-D-galactose	1623	778	969	503	1620	990	1991	1506	1586	1355	0.143	0.247
3,4-dihydroxyphenylacetic acid	695	543	3017	4875	2958	6673	2809	6192	1561	3623	0.367	0.475
3,4-dihydroxyhydrocinnamic acid NIST	99467.125[a]	137971	5720.5[b]	3827	7556.5[b]	6674	35126.3125[b]	34212	34774.75[b]	28731	0.000	0.003
3,4-dihydroxycinnamic acid	1047	1057	508	265	606	483	886	757	713	483	0.082	0.164
3,4-dihydroxybenzoic acid	2909	2946	7548	17689	7572	24210	3936	3057	2750	2563	0.632	0.693
3-(4-hydroxyphenyl)propionic acid	12266.5[a]	9011	3327.8125[b]	2464	2562.625[b]	1386	7821[ab]	4256	12039.25[a]	9076	0.000	0.000
3-(3-hydroxyphenyl)propionic acid	7195	16848	242	87	371	214	4684	10724	10840	17575	0.036	0.094
2-ketoisocaproic acid	6927	3470	4124	2643	3988	1986	5483	3167	4492	2206	0.018	0.059
2-ketobutyric acid	3808	1975	2750	1808	3284	2982	3004	1953	3441	2562	0.702	0.766
2-hydroxyhexanoic acid	5604.8125[b]	14404	27170.125[a]	18284	21814.9375[a]	14887	4118.3125[b]	4629	2798.0625[b]	3328	0.000	0.000
2-hydroxyglutaric acid	1314.5625[b]	1344	5807.25[a]	3834	6130[a]	3529	1981.1875[b]	1433	2254.5[b]	2986	0.000	0.000
2-hydroxybutanoic acid	15445.375[b]	40335	63515.25[a]	43645	88181.0625[a]	85418	11272.625[b]	14468	8544.9375[b]	7606	0.000	0.000
2-deoxytetronic acid	2397.4375[a]	2323	528.4375[b]	270	552.125[b]	355	1466[ab]	1017	1282.8125[ab]	842	0.000	0.001
2-deoxyerythritol	4348	5366	763	396	3784	6727	1293	929	1619	1743	0.049	0.122
2,4-diaminobutyric acid	3268	2236	1252	899	1743	1843	1952	1474	2585	3098	0.023	0.072
1-monopalmitin	830	388	503	395	723	433	690	234	526	289	0.041	0.105
1-kestose	427	499	411	432	343	221	457	817	256	160	0.752	0.792
1-deoxyerythritol	2690	1601	1921	2809	3494	4817	1819	729	4783	10358	0.487	0.588
1,5-anhydroglucitol	1062	1190	2031	1957	3055	4276	2440	3826	1572	1724	0.181	0.292
2,5-dihydroxypyrazine NIST	873	599	674	375	865	560	541	235	573	154	0.024	0.073
