# Supplementary material for: Effects of metronidazole on the fecal microbiome and metabolome in healthy dogs
Source: J Vet Intern Med. 2020 Aug 28;34(5):1853–66. doi: 10.1111/jvim.15871 (PMC7517498; doi:10.1111/jvim.15871)
Supplement: Supplementary file 7 — Supplementary Data S7. List of relevant metabolites detected in serum samples from group 1 (control), with mean and SD for each time point. Time points were compared with 1‐way ANOVA and adjusted for multiple comparison using Benjamini and Hochberg's False Discovery Rate, and p‐ and q‐values are presented. [file JVIM-34-1853-s007.pdf]

Group 1 Summary Statistics for serum metabolomics

	day 0		day 42			
**Compound name**	**mean**	**standard deviation**	**mean**	**standard deviation**	**p-value**	**q-value**
xylulose NIST	251	26	270	47	0.1982	0.8716
xylose	1277	357	1256	260	0.9229	0.9598
xylitol	351	111	309	55	0.4119	0.8716
xanthine	778	168	731	287	0.5640	0.8716
valine	121653	25692	118763	28427	0.7562	0.8969
uric acid	785	260	627	229	0.1942	0.8716
urea	207064	64365	140654	88491	0.0167	0.8716
uracil	429	137	341	183	0.1709	0.8716
tyrosine	36118	8856	34342	10348	0.7113	0.8969
tryptophan	81114	22953	81854	36107	0.9269	0.9598
trans-4-hydroxyproline	20492	13502	16703	13829	0.5482	0.8716
tocopherol alpha-	7906	4097	7897	2569	0.9924	0.9924
thymidine	2747	811	2625	855	0.3508	0.8716
threose	446	123	365	155	0.3851	0.8716
threonine	40909	7012	36839	7999	0.2210	0.8716
threonic acid	4262	722	3789	664	0.0765	0.8716
threitol	233	87	217	30	0.6090	0.8716
taurine	10607	3557	8621	1849	0.0963	0.8716
tagatose	1664	539	1934	506	0.3856	0.8716
sulfuric acid	2312	2609	2158	1988	0.8974	0.9565
sucrose	212	233	140	106	0.4719	0.8716
succinic acid	934	216	855	150	0.2140	0.8716
stearic acid	267385	58559	292004	62126	0.3538	0.8716
sorbitol	795	412	1079	700	0.2518	0.8716
serine	46398	8571	43778	12960	0.4848	0.8716
sebacic acid, di(2-octyl) ester NIST	1629	299	1961	355	0.1127	0.8716
saccharic acid	159	64	163	67	0.9023	0.9565
ribose	1075	352	1045	338	0.8813	0.9531
ribonic acid	402	120	480	119	0.2255	0.8716

ribitol	288	64	313	99	0.5740	0.8716
pyrophosphate	150	66	199	145	0.4638	0.8716
pseudo uridine	1796	368	1750	138	0.7611	0.8969
propane-1,3-diol NIST	1723	514	1641	271	0.6307	0.8827
proline	127660	48987	134349	75296	0.7640	0.8969
pinitol	184	149	538	758	0.2437	0.8716
phosphoethanolamine	242	150	169	61	0.2185	0.8716
phosphate	14293	3330	13876	2167	0.7535	0.8969
phenylethylamine	1874	748	2220	1140	0.4615	0.8716
phenylalanine	18685	3452	18517	3553	0.6468	0.8827
pelargonic acid	14890	2092	15634	2110	0.5127	0.8716
palmitoleic acid	5877	6482	10325	12728	0.2557	0.8716
palmitic acid	42459	12366	46066	14986	0.4077	0.8716
oxoproline	161288	32532	149084	68622	0.6783	0.8922
oxalic acid	601	65	521	141	0.0672	0.8716
ornithine	13199	7198	11560	4073	0.4772	0.8716
oleic acid	6027	4722	9363	9326	0.2392	0.8716
octanol NIST	799	136	766	153	0.5516	0.8716
octadecanol	198	40	284	106	0.0436	0.8716
N-methylalanine	15337	7480	18198	8359	0.4583	0.8716
N-acetylmannosamine	94	47	106	42	0.6688	0.8877
n-acetylglutamate	687	254	683	185	0.9734	0.9869
N-acetyl-D-hexosamine	146	40	152	38	0.7975	0.8969
myristic acid	13275	3560	16259	5883	0.0881	0.8716
myo-inositol	13038	6027	10771	4201	0.5265	0.8716
methyltetrahydrophenanthrenone NIST	1230	277	1221	208	0.9106	0.9565
methionine sulfoxide	12344	4528	12805	3774	0.7935	0.8969
methionine	7253	4163	6793	2841	0.7224	0.8969
methanolphosphate	1071	146	1464	942	0.2724	0.8716
mannose	13392	5069	15899	4868	0.3593	0.8716
maltose	309	114	354	146	0.4796	0.8716
malic acid	460	297	469	191	0.8695	0.9474
maleimide	811	352	653	73	0.2496	0.8716
lyxitol	833	195	909	281	0.5593	0.8716

lysine	37810	16692	27277	9242	0.0631	0.8716
linolenic acid	3422	2523	2703	1849	0.2222	0.8716
linoleic acid	2857	1992	3998	3517	0.1995	0.8716
leucine	95193	8869	102780	33076	0.5003	0.8716
lauric acid	5786	2140	6886	2086	0.3784	0.8716
lactic acid	321704	114560	298144	91923	0.6149	0.8716
lactamide	473	421	480	647	0.9813	0.9881
ketohexose	255	76	274	98	0.6630	0.8877
isothreonic acid	803	136	842	108	0.5321	0.8716
isoleucine	46660	11892	45171	9896	0.8091	0.8969
isoheptadecanoic acid NIST	735	172	906	267	0.1075	0.8716
isocitric acid	538	112	591	176	0.3073	0.8716
inosine	1250	715	1241	676	0.9492	0.9759
indoxyl sulfate	418	175	287	204	0.1996	0.8716
indole-3-lactate	1256	479	1302	774	0.7457	0.8969
indole-3-acetate	653	152	581	239	0.3223	0.8716
hypoxanthine	2371	1230	2350	1632	0.9685	0.9869
hydroxylamine	45572	18065	35985	27155	0.3040	0.8716
hydroxycarbamate NIST	8362	3680	6144	4511	0.2442	0.8716
hydroquinone	3459	2390	2411	2453	0.2882	0.8716
histidine	3751	1683	3356	1006	0.5649	0.8716
hexuronic acid	4303	2486	3711	3396	0.7179	0.8969
hexose	1310	643	1527	596	0.4412	0.8716
hexitol	506	277	455	226	0.3316	0.8716
heptadecanoic acid	5060	1355	6019	1471	0.0559	0.8716
glycolic acid	2279	864	1799	639	0.1643	0.8716
glycine	58874	35297	63650	40746	0.7995	0.8969
glycerol-alpha-phosphate	6819	1394	6600	1378	0.6111	0.8716
glycerol	29427	14567	31712	17295	0.6530	0.8828
glyceric acid	2352	450	2391	255	0.7965	0.8969
glutamine	95116	55735	108893	62287	0.7158	0.8969
glutamic acid	6865	1705	7211	2418	0.6469	0.8827
glucose-6-phosphate	343	154	283	142	0.4127	0.8716
glucose	253644	45318	289158	69314	0.3252	0.8716

gluconic acid	137	31	144	52	0.6352	0.8827
fumaric acid	450	151	474	180	0.3430	0.8716
fructose	33966	10083	39784	10534	0.3409	0.8716
ethanolamine	995	140	1070	172	0.3176	0.8716
erythrose	225	40	238	29	0.3955	0.8716
erythritol	736	94	791	72	0.1748	0.8716
dodecanoic acid, isopropanol ester NIST	173	78	179	68	0.9041	0.9565
doconexent NIST	1327	588	1218	287	0.6022	0.8716
cystine	2690	867	2947	928	0.0890	0.8716
cysteine	883	312	798	174	0.5467	0.8716
creatinine	10625	3383	9194	2029	0.0602	0.8716
conduritol-beta-expoxide	584	94	791	468	0.2551	0.8716
citrulline	2072	827	2347	631	0.4127	0.8716
citric acid	29726	7001	32294	8036	0.2987	0.8716
cholesterol	131525	17127	132713	13260	0.8657	0.9474
caprylic acid	3914	759	3663	623	0.5079	0.8716
capric acid	873	155	946	158	0.3504	0.8716
beta-glycerolphosphate	194	43	220	92	0.4745	0.8716
beta-alanine	189	71	176	77	0.6966	0.8969
benzoic acid	8923	1168	6952	1569	0.0060	0.8716
behenic acid	767	142	788	130	0.7873	0.8969
azelaic acid	225	188	117	21	0.1574	0.8716
aspartic acid	1202	437	1104	318	0.5904	0.8716
asparagine	4858	1746	4459	1875	0.5301	0.8716
arachidonic acid	11843	3231	12394	3414	0.5954	0.8716
arachidic acid	2566	544	2803	718	0.4972	0.8716
aminomalonate	2335	747	1806	1540	0.3814	0.8716
allantoic acid	1200	252	1133	223	0.4928	0.8716
alanine-alanine	1342	554	1258	456	0.7348	0.8969
alanine	285596	49726	306574	58049	0.4757	0.8716
adipic acid	489	165	388	158	0.2652	0.8716
aconitic acid	280	69	316	79	0.2270	0.8716
acetophenone NIST	1856	582	2560	629	0.1006	0.8716
5-methoxytryptamine	345	214	393	159	0.4785	0.8716

5-aminovaleric acid	351	107	309	90	0.4630	0.8716
4-hydroxyquinoline-2-carboxylic acid	387	213	342	183	0.5001	0.8716
3-phosphoglycerate	135	40	122	45	0.6107	0.8716
3-hydroxybutyric acid	2538	1616	3135	1985	0.4982	0.8716
2,3-dihydroxybutanoic acid NIST	183	40	147	79	0.1097	0.8716
2-ketoisocaproic acid	2374	1442	2066	321	0.5897	0.8716
2-hydroxyvaleric acid	1219	410	1191	243	0.8109	0.8969
2-hydroxypyrazinyl-2-propenoic acid ethyl ester NIST	781	307	570	324	0.1479	0.8716
2-hydroxyglutaric acid	307	130	443	346	0.3616	0.8716
2-hydroxybutanoic acid	11559	8311	12702	7435	0.4818	0.8716
2-deoxytetronic acid	281	75	348	57	0.0964	0.8716
1,5-anhydroglucitol	30423	11335	31415	14161	0.7348	0.8969
1-monostearin	350	142	335	87	0.7854	0.8969
1-monopalmitin	355	152	377	162	0.7054	0.8969
1-monoolein	534	128	649	293	0.2263	0.8716

p-values adjusted by Benjamini and Hochberg FDR
