# Supplementary material for: Effects of metronidazole on the fecal microbiome and metabolome in healthy dogs
Source: J Vet Intern Med. 2020 Aug 28;34(5):1853–66. doi: 10.1111/jvim.15871 (PMC7517498; doi:10.1111/jvim.15871)
Supplement: Supplementary file 8 — Supplementary Data S8. List of relevant metabolites detected in serum samples from group 2 during the hydrolyzed protein diet trial, with mean and SD for each time point. Time points were compared with 1‐way ANOVA and adjusted for multiple comparison using Benjamini and Hochberg's False Discovery Rate, and p‐ and q‐values are presented. [file JVIM-34-1853-s008.pdf]

Group 2 diet Summary Statistics for serum metabolomics

	day 0		day 42			
**Compound name**	**mean**	**standard deviation**	**mean**	**standard deviation**	**p-value**	**q-value**
xylulose NIST	224	35	274	98	0.1794	0.5136
xylose	1161	185	1146	345	0.9217	0.9822
xylitol	294	84	280	89	0.6337	0.8641
xanthine	714	178	641	340	0.4902	0.7952
valine	111466	30099	125211	26638	0.4153	0.7394
uric acid	896	261	639	199	0.069	0.3713
urea	161390	46196	169443	34755	0.7599	0.9245
uracil	338	76	459	480	0.4605	0.7836
tyrosine	27664	4605	41845	17765	0.034	0.3189
tryptophan	80168	25270	99206	26540	0.0523	0.3189
trans-4-hydroxyproline	12476	4979	14687	22960	0.8125	0.9367
tocopherol alpha-	7105	3030	5498	2318	0.0178	0.3147
thymidine	2525	1330	2116	635	0.2284	0.5955
threose	346	186	402	101	0.4863	0.7952
threonine	34939	8718	39353	11552	0.0886	0.4079
threonic acid	4572	1066	3405	783	0.0013	0.1241
threitol	234	49	222	52	0.6292	0.8641
taurine	8880	2504	8292	2871	0.5769	0.8240
tagatose	1605	405	1959	391	0.0765	0.3851
sulfuric acid	3753	4063	1997	1255	0.317	0.6340
sucrose	107	51	84	38	0.2874	0.6111
succinic acid	953	256	947	295	0.9296	0.9826
stearic acid	251606	67436	244036	35580	0.7762	0.9289
sorbitol	1105	304	773	357	0.0527	0.3189
serine	34598	6932	43124	15855	0.0986	0.4113
sebacic acid, di(2-octyl) ester NIST	1752	515	1802	512	0.749	0.9245
saccharic acid	120	32	155	40	0.0804	0.3913
ribose	642	304	1256	666	0.0127	0.3147
ribonic acid	336	158	333	150	0.9392	0.9826

ribitol	271	79	286	54	0.66	0.8760
pyrophosphate	165	74	171	62	0.6412	0.8641
pseudo uridine	1689	415	1931	401	0.0516	0.3189
propane-1,3-diol NIST	2278	1179	1687	457	0.1059	0.4295
proline	92145	27247	120847	66550	0.2222	0.5898
pinitol	248	212	245	308	0.9807	0.9943
phosphoethanolamine	144	58	199	139	0.2683	0.6111
phosphate	12320	3524	10746	2559	0.2922	0.6111
phenylethylamine	2516	830	4268	1981	0.0194	0.3147
phenylalanine	17041	3701	18597	3563	0.2982	0.6132
pelargonic acid	16010	3622	16398	2366	0.7003	0.9114
palmitoleic acid	8160	3959	6054	6267	0.2352	0.6006
palmitic acid	42998	12664	38970	9442	0.4749	0.7879
oxoproline	188434	49559	164625	83125	0.2028	0.5534
oxalic acid	584	260	505	132	0.3957	0.7394
ornithine	10741	3298	14182	3239	0.0365	0.3189
oleic acid	9755	5362	6046	5643	0.1581	0.4827
octanol NIST	846	247	857	140	0.9141	0.9813
octadecanol	180	44	244	108	0.162	0.4827
N-methylalanine	16293	6519	11636	10948	0.2741	0.6111
N-acetylmannosamine	94	37	96	43	0.9008	0.9813
n-acetylglutamate	765	372	637	386	0.5159	0.7972
N-acetyl-D-hexosamine	139	35	146	54	0.803	0.9367
myristic acid	13568	3757	14078	3163	0.7679	0.9266
myo-inositol	10873	2909	9291	4775	0.4109	0.7394
methyltetrahydrophenanthrenone NIST	1104	352	1027	224	0.2812	0.6111
methionine sulfoxide	9205	4104	11753	4005	0.1343	0.4669
methionine	6647	1843	3973	1875	0.0392	0.3189
methanolphosphate	954	366	1171	910	0.5332	0.8029
mannose	14446	5440	14038	3428	0.8006	0.9367
maltose	210	111	250	84	0.1618	0.4827
malic acid	540	181	511	244	0.5634	0.8144
maleimide	714	174	669	217	0.6146	0.8546
lyxitol	793	263	663	128	0.3078	0.6242

lysine	28575	13428	31691	10434	0.4071	0.7394
linolenic acid	4114	1957	3039	848	0.1574	0.4827
linoleic acid	3839	1054	3052	1864	0.293	0.6111
leucine	85094	11973	84398	24474	0.9499	0.9826
lauric acid	6443	1616	10009	3467	0.022	0.3189
lactic acid	452065	227307	249486	130071	0.0039	0.1606
lactamide	823	897	402	337	0.1447	0.4827
ketohexose	271	92	302	67	0.4111	0.7394
isothreonic acid	833	241	762	127	0.4555	0.7836
isoleucine	40976	8287	49634	10714	0.1541	0.4827
isoheptadecanoic acid NIST	918	406	669	159	0.0957	0.4109
isocitric acid	616	180	656	239	0.4563	0.7836
inosine	854	415	1396	637	0.0712	0.3713
indoxyl sulfate	217	123	250	113	0.5016	0.7972
indole-3-lactate	1222	407	1178	610	0.7275	0.9114
indole-3-acetate	555	192	620	189	0.2912	0.6111
hypoxanthine	2794	966	2972	2633	0.8469	0.9367
hydroxylamine	38189	24679	42378	21294	0.7095	0.9114
hydroxycarbamate NIST	7571	5350	7577	4501	0.998	0.9980
hydroquinone	1576	1412	1413	881	0.546	0.8029
histidine	3270	1161	3837	935	0.1253	0.4619
hexuronic acid	3573	2411	3683	2731	0.9076	0.9813
hexose	1401	393	1599	488	0.3622	0.7051
hexitol	688	221	398	136	0.0017	0.1241
heptadecanoic acid	6181	2296	4317	1247	0.0445	0.3189
glycolic acid	1934	701	2684	473	0.0894	0.4079
glycine	55149	27607	54818	26821	0.9598	0.9826
glycerol-alpha-phosphate	5992	2452	5891	1977	0.8153	0.9367
glycerol	28199	7611	28426	17008	0.9624	0.9826
glyceric acid	2457	580	2380	851	0.8436	0.9367
glutamine	74439	31909	132092	69380	0.015	0.3147
glutamic acid	7741	1388	11141	4989	0.1229	0.4619
glucose-6-phosphate	256	173	331	119	0.2658	0.6111
glucose	247953	50873	305969	58897	0.0186	0.3147

gluconic acid	114	58	163	57	0.1297	0.4619
fumaric acid	516	88	421	134	0.0371	0.3189
fructose	32933	9089	40175	7807	0.0931	0.4109
ethanolamine	836	134	888	234	0.6451	0.8641
erythrose	222	70	209	25	0.5499	0.8029
erythritol	819	193	735	135	0.1758	0.5133
dodecanoic acid, isopropanol ester NIST	136	60	159	42	0.2808	0.6111
doconexent NIST	1281	840	1118	672	0.3355	0.6619
cystine	2957	686	3319	1083	0.2731	0.6111
cysteine	932	360	1015	464	0.5813	0.8240
creatinine	8555	2226	8033	2649	0.7212	0.9114
conduritol-beta-expoxide	647	229	446	172	0.0286	0.3189
citrulline	1972	434	3108	1532	0.0268	0.3189
citric acid	33054	9501	33929	11329	0.6974	0.9114
cholesterol	107336	29450	110296	22683	0.541	0.8029
caprylic acid	3857	1005	3973	745	0.8033	0.9367
capric acid	885	138	1036	263	0.0652	0.3661
beta-glycerolphosphate	180	42	178	67	0.9522	0.9826
beta-alanine	126	34	182	73	0.0543	0.3189
benzoic acid	7407	1904	7238	1475	0.8456	0.9367
behenic acid	820	201	859	135	0.6067	0.8517
azelaic acid	119	49	122	22	0.882	0.9682
aspartic acid	1194	427	1160	383	0.7275	0.9114
asparagine	3753	884	6046	2730	0.0424	0.3189
arachidonic acid	11494	2883	10599	2827	0.5109	0.7972
arachidic acid	2211	602	2614	321	0.1188	0.4619
aminomalonate	1832	845	1927	861	0.8237	0.9367
allantoic acid	1103	385	995	295	0.4616	0.7836
alanine-alanine	1403	284	1705	949	0.3861	0.7394
alanine	366267	192403	367450	81172	0.9882	0.9950
adipic acid	383	128	470	76	0.2047	0.5534
aconitic acid	251	67	286	89	0.5086	0.7972
acetophenone NIST	1922	660	2414	1469	0.2713	0.6111
5-methoxytryptamine	351	177	499	320	0.2386	0.6006

5-aminovaleric acid	268	75	295	85	0.5457	0.8029
4-hydroxyquinoline-2-carboxylic acid	274	180	375	161	0.1284	0.4619
3-phosphoglycerate	107	48	114	35	0.7304	0.9114
3-hydroxybutyric acid	2644	857	2308	1357	0.3927	0.7394
2,3-dihydroxybutanoic acid NIST	164	44	175	46	0.5187	0.7972
2-ketoisocaproic acid	3205	2630	2469	1459	0.1491	0.4827
2-hydroxyvaleric acid	1168	305	1062	450	0.4728	0.7879
2-hydroxypyrazinyl-2-propenoic acid ethyl ester NIST	631	346	663	277	0.8349	0.9367
2-hydroxyglutaric acid	272	148	118	60	0.0407	0.3189
2-hydroxybutanoic acid	13371	8193	7098	5216	0.0546	0.3189
2-deoxytetronic acid	361	92	295	100	0.0044	0.1606
1,5-anhydroglucitol	26977	7488	34451	7585	0.0345	0.3189
1-monostearin	339	101	286	87	0.2919	0.6111
1-monopalmitin	248	157	275	137	0.7593	0.9245
1-monoolein	499	96	427	109	0.1832	0.5144

p-values adjusted by Benjamini and Hochberg FDR
