# Supplementary material for: Effects of metronidazole on the fecal microbiome and metabolome in healthy dogs
Source: J Vet Intern Med. 2020 Aug 28;34(5):1853–66. doi: 10.1111/jvim.15871 (PMC7517498; doi:10.1111/jvim.15871)
Supplement: Supplementary file 9 — Supplementary Data S9. List of relevant metabolites detected in serum samples from groups 2 and 3 during the metronidazole trial, with mean and SD for each time point. Time points were compared with 1‐way ANOVA and adjusted for multiple comparison using Benjamini and Hochberg's False Discovery Rate, and p‐ and q‐values are presented [file JVIM-34-1853-s009.pdf]

Group 2 metronidazole and Group 3 Combined Summary Statistics for serum metabolomics

	day 0		day 14		day 42			
Compound Name	mean	standard deviation	mean	standard deviation	mean	standard deviation	p-value	q-value
xylulose NIST	236	95	233	68	236	67	0.986	0.987
xylose	1198	288	1193	217	1208	304	0.982	0.987
xylitol	301	108	277	66	270	81	0.258	0.734
xanthine	685	263	791	164	783	281	0.264	0.734
valine	130290	31480	125188	24384	126148	21064	0.735	0.925
uric acid	723	214	781	245	692	161	0.313	0.776
urea	165576	55953	164144	53849	178157	58248	0.699	0.907
uracil	427	352	377	109	372	178	0.714	0.913
tyrosine	37539	13410	42459	14346	35056	8370	0.049	0.422
tryptophan	94000	21854	91724	29460	93617	31886	0.900	0.968
trans-4-hydroxyproline	17796	18076	9526	4270	20206	22640	0.058	0.424
tocopherol alpha-	5369	1908	6220	2216	5149	2068	0.008	0.181
thymidine	2638	1090	2670	873	2501	1362	0.573	0.873
threose	378	105	424	116	363	146	0.388	0.810
threonine	41526	10982	42310	8466	40850	11062	0.600	0.873
threonic acid	4052	1066	3617	804	3403	1455	0.112	0.511
threitol	236	100	246	77	261	98	0.634	0.876
taurine	8656	2634	7927	2208	9893	3851	0.087	0.438
tagatose	1785	382	1630	522	1599	714	0.418	0.823
sulfuric acid	2110	1498	871	672	1162	1468	0.034	0.407
sucrose	116	71	106	85	98	73	0.815	0.952
succinic acid	933	257	895	194	891	213	0.826	0.952
stearic acid	230003	41026	239202	63104	225371	49831	0.755	0.934
sorbitol	944	387	751	376	1119	650	0.075	0.438
serine	40942	12382	42090	9158	41797	12936	0.891	0.968
sebacic acid, di(2-octyl) ester NIST	1591	438	1841	423	1658	488	0.187	0.734
saccharic acid	169	71	136	51	155	74	0.141	0.590
ribose	1121	569	1261	471	1149	465	0.447	0.826
ribonic acid	337.875[b]	111	989.6875[a]	589	363.375[b]	126	0.000	0.000

ribitol	286	53	277	89	261	67	0.610	0.873
pyrophosphate	190	73	147	58	162	56	0.074	0.438
pseudo uridine	1851	459	1791	498	1759	622	0.542	0.873
propane-1,3-diol NIST	1677	476	1730	531	1812	776	0.702	0.907
proline	125651	50796	103658	47641	139321	87608	0.085	0.438
pinitol	323	447	243	338	268	435	0.372	0.799
phosphoethanolamine	213	122	175	94	175	95	0.429	0.823
phosphate	11067	2545	12328	2212	10734	2618	0.049	0.422
phenylethylamine	3256	1768	3144	1666	3169	1314	0.958	0.987
phenylalanine	17281	2945	18403	3174	17612	3028	0.250	0.734
pelargonic acid	15263	2692	15727	2578	15579	2681	0.833	0.952
palmitoleic acid	6020	4436	10504	10619	5729	5433	0.126	0.556
palmitic acid	38060	8054	42457	14564	36719	11616	0.348	0.781
oxoproline	157864	66687	155763	56107	146233	53329	0.824	0.952
oxalic acid	567	143	480	111	478	231	0.264	0.734
ornithine	14618	3251	12546	2706	14082	4133	0.201	0.734
oleic acid	6003	4090	9037	8123	5854	3777	0.196	0.734
octanol NIST	797	160	824	170	775	173	0.581	0.873
octadecanol	212	94	255	73	236	78	0.353	0.781
N-methylalanine	16030	11801	14009	8086	13051	5890	0.470	0.847
N-acetylmannosamine	100	39	110	43	99	46	0.650	0.876
n-acetylglutamate	660	352	806	360	590	305	0.139	0.590
N-acetyl-D-hexosamine	135	46	157	35	165	117	0.435	0.823
myristic acid	11445	3512	16181	10232	13221	4701	0.068	0.438
myo-inositol	12465	6873	12227	7310	11848	7219	0.901	0.968
methyltetrahydrophenanthrenone NIST	1030	205	1278	329	1032	282	0.018	0.259
methionine sulfoxide	11213	4198	11149	4236	14277	5768	0.054	0.423
methionine	6411	3194	5867	3295	6756	4342	0.719	0.913
methanolphosphate	1068	793	1099	358	942	364	0.658	0.876
mannose	13362	2687	13658	3859	13130	4109	0.885	0.968
maltose	254	96	258	114	246	83	0.916	0.969
malic acid	459	186	512	186	460	183	0.467	0.847
maleimide	736	259	777	167	1834	3585	0.251	0.734
lyxitol	953	412	717	239	838	210	0.070	0.438

lysine	36086	12295	31212	11464	33150	10270	0.251	0.734
linolenic acid	3575	1288	4073	2420	3073	1761	0.266	0.734
linoleic acid	3246	1479	4213	2246	3465	2077	0.279	0.754
leucine	96741	25192	88881	30560	93640	27193	0.504	0.848
lauric acid	7351	3754	10149	8828	8081	4357	0.204	0.734
lactic acid	282295	133967	342710	153882	326558	185633	0.313	0.776
lactamide	409	348	442	360	637	1054	0.597	0.873
ketohexose	286	60	278	100	253	117	0.423	0.823
isothreonic acid	749.8125[b]	140	1478[a]	536	798[b]	266	0.000	0.000
isoleucine	49564	9521	49618	9416	47213	9184	0.627	0.876
isoheptadecanoic acid NIST	578	158	697	314	621	160	0.265	0.734
isocitric acid	661	218	662	199	618	270	0.660	0.876
inosine	1321	555	1168	785	982	429	0.087	0.438
indoxyl sulfate	223	164	230	153	218	109	0.967	0.987
indole-3-lactate	1066	470	1194	547	1124	570	0.491	0.848
indole-3-acetate	619	177	577	360	703	294	0.353	0.781
hypoxanthine	2728	2107	2522	1471	2486	943	0.798	0.952
hydroxylamine	40856	19774	33792	21975	33247	23658	0.566	0.873
hydroxycarbamate NIST	7163	3970	6049	4201	5793	3974	0.636	0.876
hydroquinone	2165	2131	322	147	1727	2819	0.055	0.423
histidine	4363	1228	3904	1037	4195	1014	0.400	0.811
hexuronic acid	4240	2892	3978	2800	4120	4701	0.961	0.987
hexose	1415	522	1378	491	1345	621	0.910	0.969
hexitol	529	274	498	363	573	340	0.335	0.781
heptadecanoic acid	3990	1015	4456	1626	3996	1006	0.494	0.848
glycolic acid	2274	643	2338	894	1555	614	0.008	0.181
glycine	53636	26949	55163	22873	71065	48373	0.261	0.734
glycerol-alpha-phosphate	6926	1824	7286	2419	6823	1844	0.567	0.873
glycerol	28195	12293	36221	17647	34827	40571	0.648	0.876
glyceric acid	2395	671	3030	674	2805	878	0.012	0.211
glutamine	119048	59589	92287	38479	129520	73049	0.190	0.734
glutamic acid	9880	3806	9053	2229	9323	4155	0.779	0.952
glucose-6-phosphate	324	177	374	223	400	380	0.598	0.873
glucose	306761	55609	289363	53895	282066	78997	0.328	0.781

gluconic acid	140	57	218	113	133	52	0.009	0.181
fumaric acid	431	97	503	127	448	106	0.112	0.511
fructose	37609	7168	34472	10246	35132	11765	0.440	0.823
ethanolamine	915.6875[b]	192	4524.6875[a]	3463	1037.5[b]	539	0.000	0.001
erythrose	207	33	199	31	216	62	0.579	0.873
erythritol	823	158	771	176	828	226	0.484	0.848
dodecanoic acid, isopropanol ester NIST	153	44	152	40	156	49	0.951	0.987
doconexent NIST	1101	507	1121	408	1084	585	0.891	0.968
cystine	3182	956	2870	787	2768	1269	0.262	0.734
cysteine	987	375	1011	488	941	320	0.832	0.952
creatinine	9329	2889	10666	3799	9959	2789	0.322	0.781
conduritol-beta-expoxide	580	286	507	187	540	304	0.410	0.820
citrulline	2890	1198	2521	636	3029	1272	0.312	0.776
citric acid	35503	10203	33977	9959	34867	15295	0.864	0.963
cholesterol	105660.25[a]	20712	111740.75[a]	29048	94568.5625[b]	21791	0.001	0.042
caprylic acid	3798	820	3980	658	3913	803	0.644	0.876
capric acid	913	244	996	238	923	173	0.352	0.781
beta-glycerolphosphate	198	57	208	88	210	74	0.862	0.963
beta-alanine	189	72	155	47	152	54	0.097	0.474
benzoic acid	7158	1469	11415	13528	7019	1286	0.235	0.734
behenic acid	767	155	856	189	711	154	0.071	0.438
azelaic acid	124	24	113	36	102	48	0.266	0.734
aspartic acid	1085	338	1258	473	1485	1355	0.365	0.795
asparagine	5337	2041	4646	1646	5037	2466	0.506	0.848
arachidonic acid	12380	3537	12405	3564	12069	3743	0.798	0.952
arachidic acid	2333	467	2414	661	2213	471	0.594	0.873
aminomalonate	1949	828	1591	738	1971	999	0.302	0.776
allantoic acid	1169	484	1092	372	1280	835	0.295	0.776
alanine-alanine	1468	728	1711	638	1227	783	0.037	0.407
alanine	355581	69205	349593	101388	332294	101799	0.605	0.873
adipic acid	438	118	417	90	396	100	0.595	0.873
aconitic acid	292	76	329	107	310	93	0.378	0.801
acetophenone NIST	2205	1138	2811	1053	2268	780	0.215	0.734
5-methoxytryptamine	446	256	479	350	445	313	0.941	0.987

5-aminovaleric acid	421	341	290	88	545	341	0.020	0.259
4-hydroxyquinoline-2-carboxylic acid	280	156	303	210	285	182	0.835	0.952
3-phosphoglycerate	116	39	119	44	141	127	0.535	0.873
3-hydroxybutyric acid	2637	1134	2654	843	1872	805	0.014	0.220
2,3-dihydroxybutanoic acid NIST	176	45	167	61	165	74	0.755	0.934
2-ketoisocaproic acid	3142	1559	2464	1389	2403	1209	0.045	0.422
2-hydroxyvaleric acid	1153	425	1132	253	1145	482	0.987	0.987
2-hydroxypyrazinyl-2-propenoic acid ethyl ester NIST	680	277	624	307	578	352	0.673	0.885
2-hydroxyglutaric acid	255	257	354	313	246	210	0.039	0.407
2-hydroxybutanoic acid	9885	5773	8408	4390	8808	4598	0.600	0.873
2-deoxytetronic acid	296	72	344	104	287	93	0.078	0.438
1,5-anhydroglucitol	31703	7873	32409	9609	31622	9570	0.862	0.963
1-monostearin	288	65	316	83	336	161	0.483	0.848
1-monopalmitin	268	105	302	135	286	224	0.794	0.952
1-monoolein	511	186	623	352	583	577	0.397	0.811

p-values adjusted by Benjamini and Hochberg FDR

Groups not sharing a common letter are significantly different (Tukey-Kramer HSD)
