# Supplementary figures and images for: Effects of metronidazole on the fecal microbiome and metabolome in healthy dogs
Source: J Vet Intern Med. 2020 Aug 28;34(5):1853–66. doi: 10.1111/jvim.15871 (PMC7517498; doi:10.1111/jvim.15871)

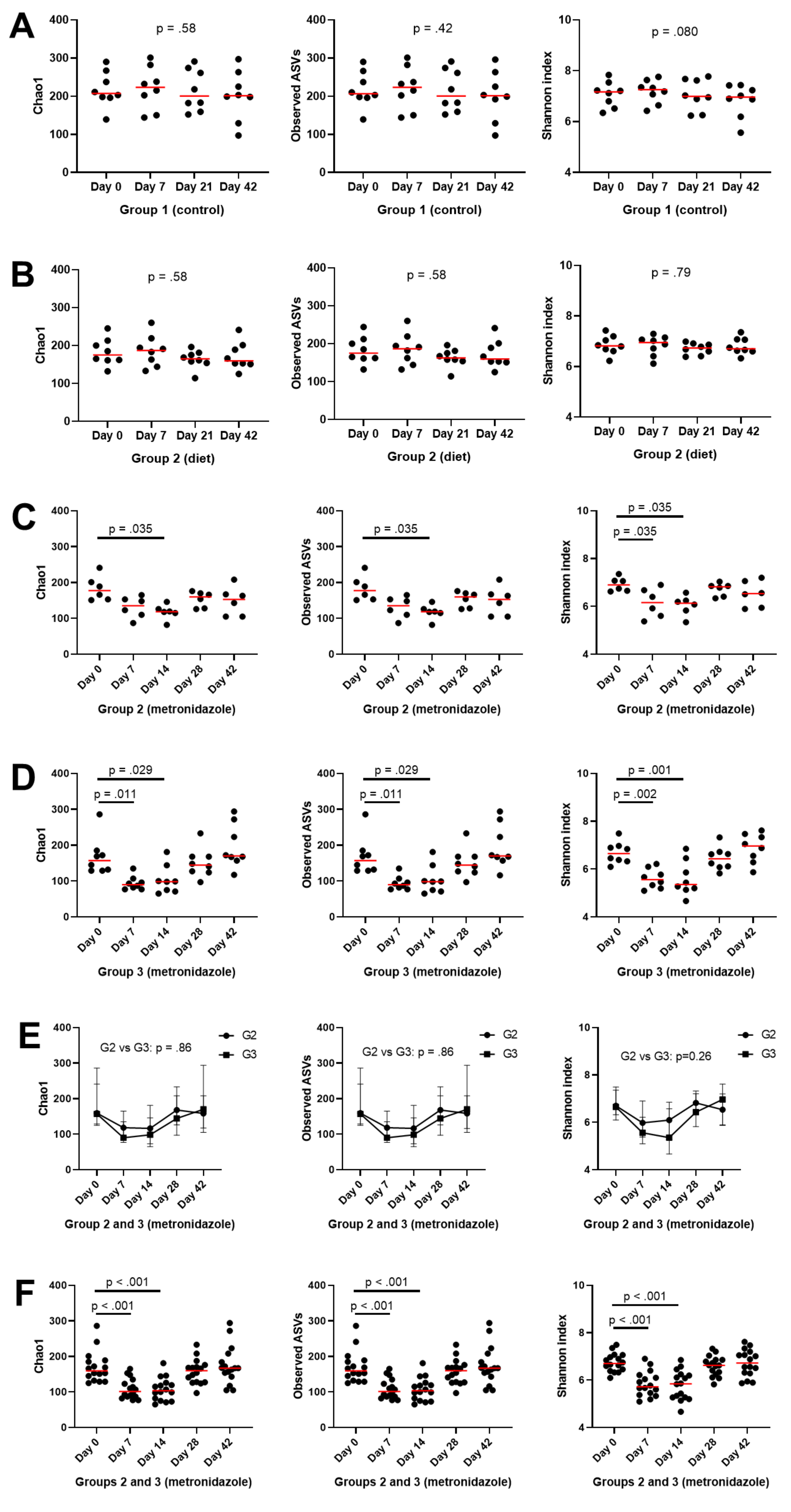

Supplement: Supplementary file 10 — Supplementary Figure S1. Alpha diversity for (A) group 1 (control), (B) groups 2 (hydrolyzed protein diet trial), (C) group 2 (metronidazole trial), (D) group 3 (metronidazole trial), and (E) a comparison between groups 2 and 3 during the metronidazole trial. Species richness (Chao 1 and Observed ASVs) and evenness (Shannon index) were not significantly affected by time (A) or diet change (B). Metronidazole administration significantly decreased all alpha diversity parameters in both group 2 (C) and 3 (D, days 7 and 14), but all parameters recovered after discontinuation of the antibiotic. No difference in the response to metronidazole was observed between group 2, which was concomitantly in a hydrolyzed diet, and group 3, which remained on their original diets (E). [file JVIM-34-1853-s010.tiff]

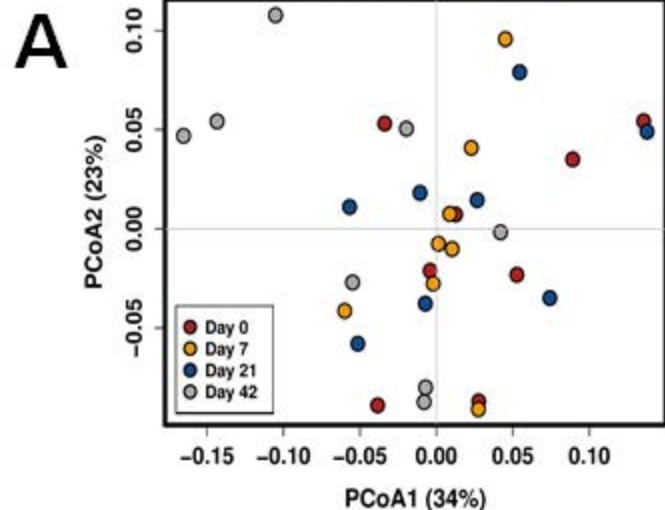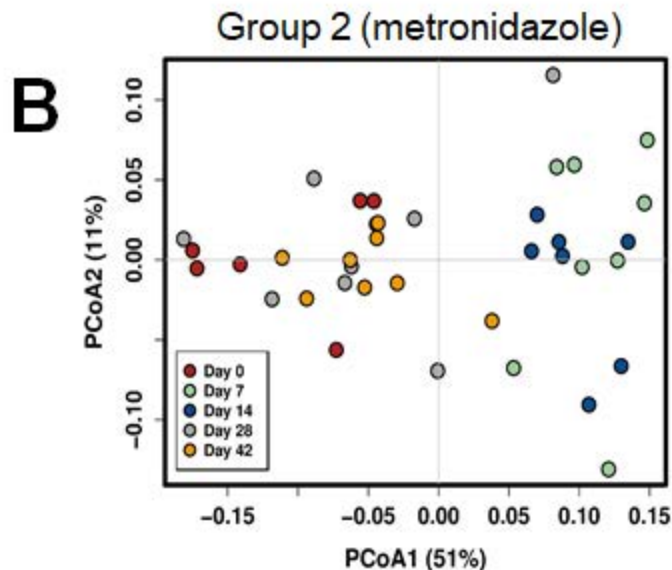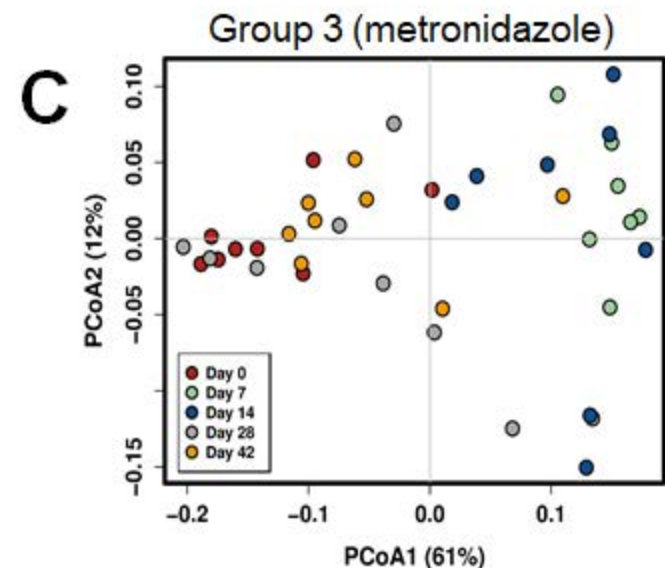

**D** ANOSIM, group 2 vs 3 (metronidazole)

| Group 2 vs group 3 | R-value | p-value |
|--------------------|---------|---------|
| Day 0              | .131    | .076    |
| Day 7              | .159    | .057    |
| Day 14             | .025    | .32     |
| Day 21             | -.016   | .50     |
| Day 42             | .285    | .016    |

Supplement: Supplementary file 11 — Supplementary Figure S2. Principal Coordinate Analysis (PCoA) of weighted UniFrac distances of 16S rRNA genes for (A) group 1 (control), (B) group 2 (metronidazole trial), (C) group 3 (metronidazole trial), and (D) ANOSIM comparison between groups 2 and 3 during metronidazole trial. Microbial communities were not significantly affected by time (A, P = .73). Metronidazole administration, as shown on day 7 (green) and day 14 (blue), significantly shifted microbial communities in both group 2 (B, P = .001) and 3 (C, P < .001). After discontinuation of treatment, microbial communities shifted back towards baseline samples, indicating a recovery of the microbiome. (D) No difference in the response to metronidazole was observed between group 2 (B), which was concomitantly in a hydrolyzed diet, and group 3 (C), which remained on their original diets, before (day 0), during (days 7 and 14), or after (day 21) metronidazole administration. A significant difference was seen at day 42 (D). [file JVIM-34-1853-s011.pdf]

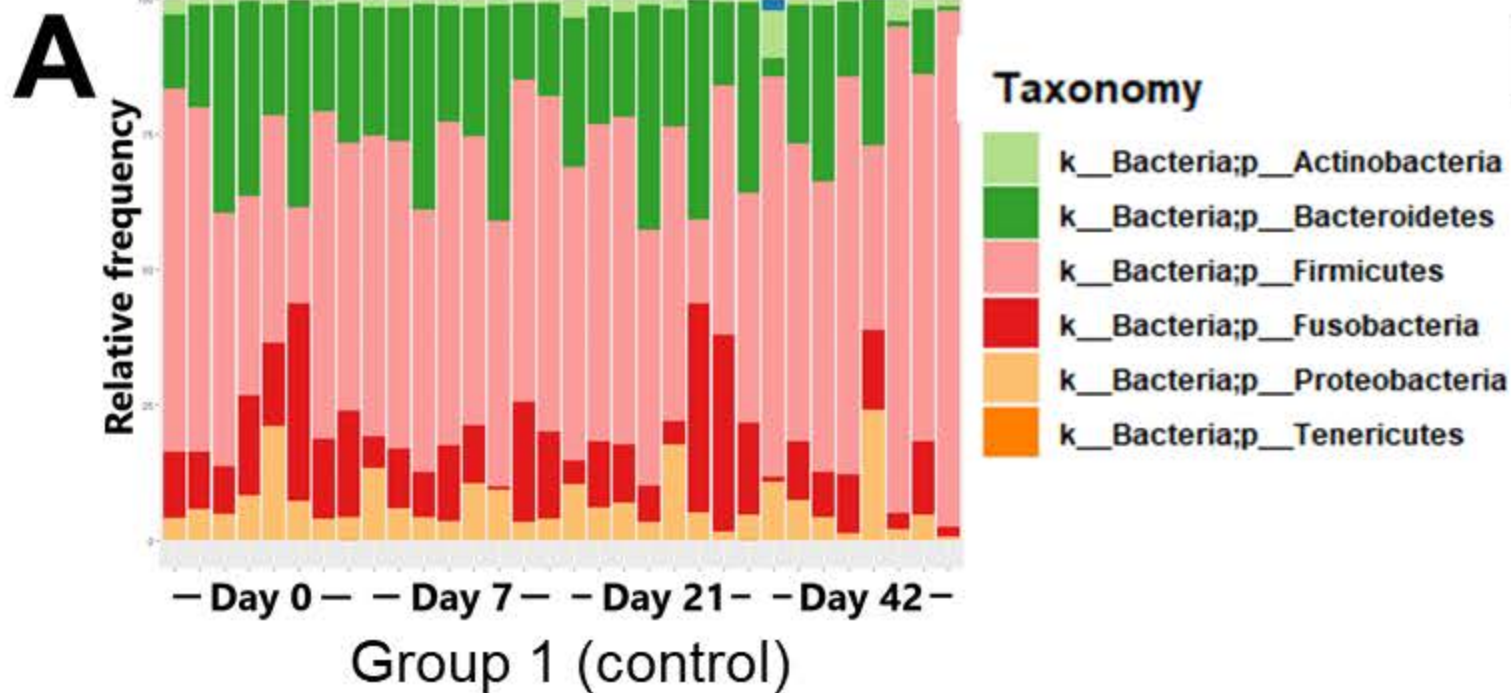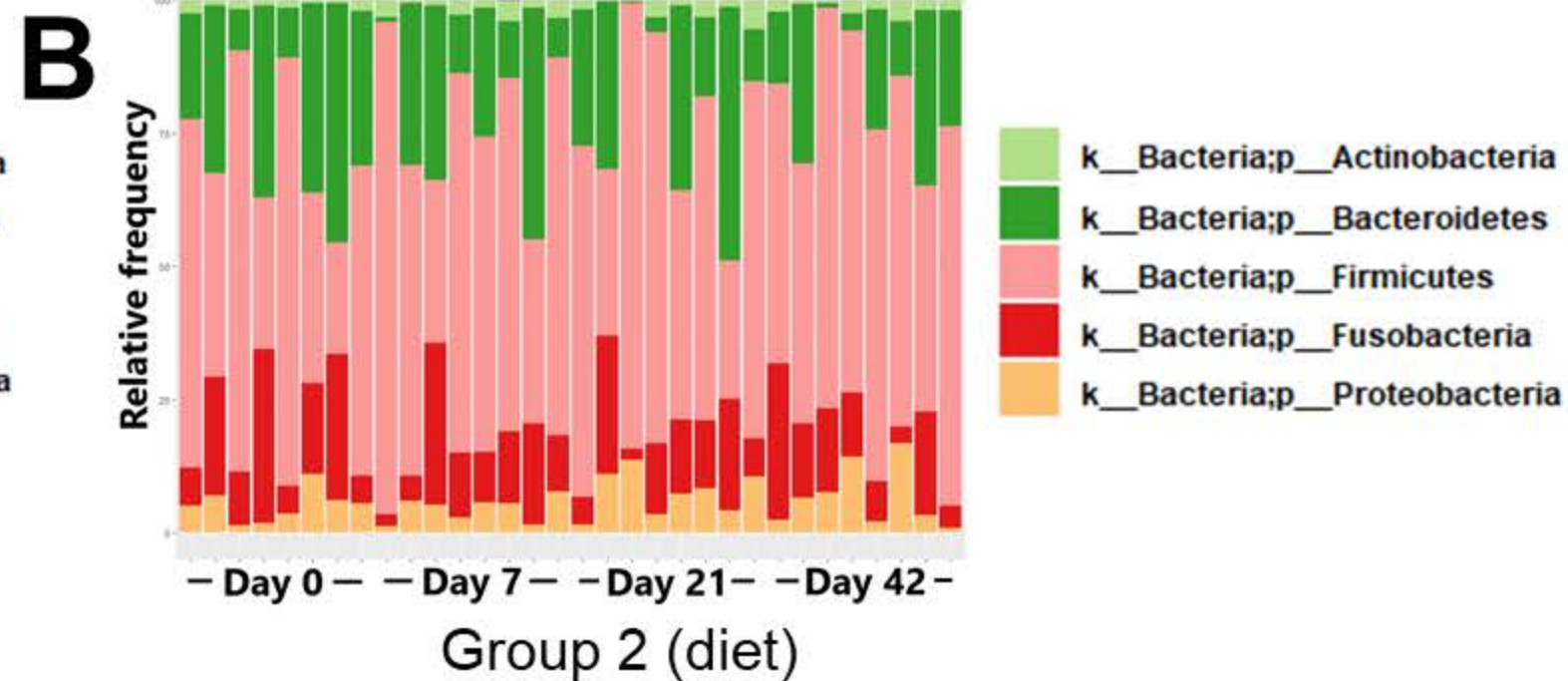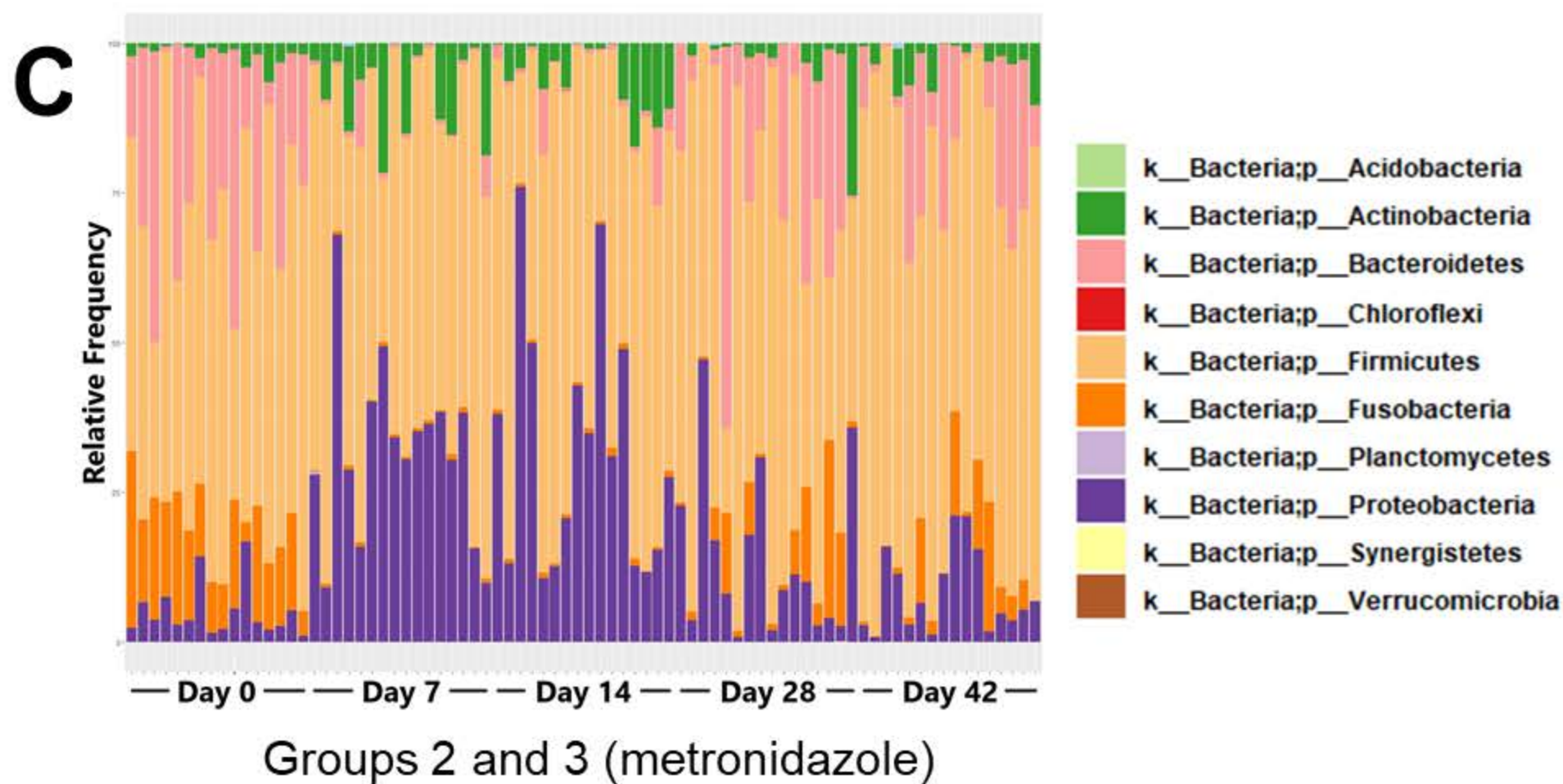

Supplement: Supplementary file 12 — Supplementary Figure S3. Phylum bar graphs from (A) group 1, (B) group 2 during the hydrolyzed protein diet trial, An (C) groups 2 and 3 during the administration of metronidazole. No significant variation is observed in 3A and 3B over time. In 3C, instead, metronidazole administration caused a significant change with decreased Bacteroidetes and Fusobacteria, and increased Proteobacteria and Actinobacteria on days 7 and 14. After the discontinuation of metronidazole, Bacteroidetes, Proteobacteria and Actinobacteria returned to abundances similar to baseline, but Fusobacteria remained significantly decreased (P = .025). [file JVIM-34-1853-s012.pdf]

**A**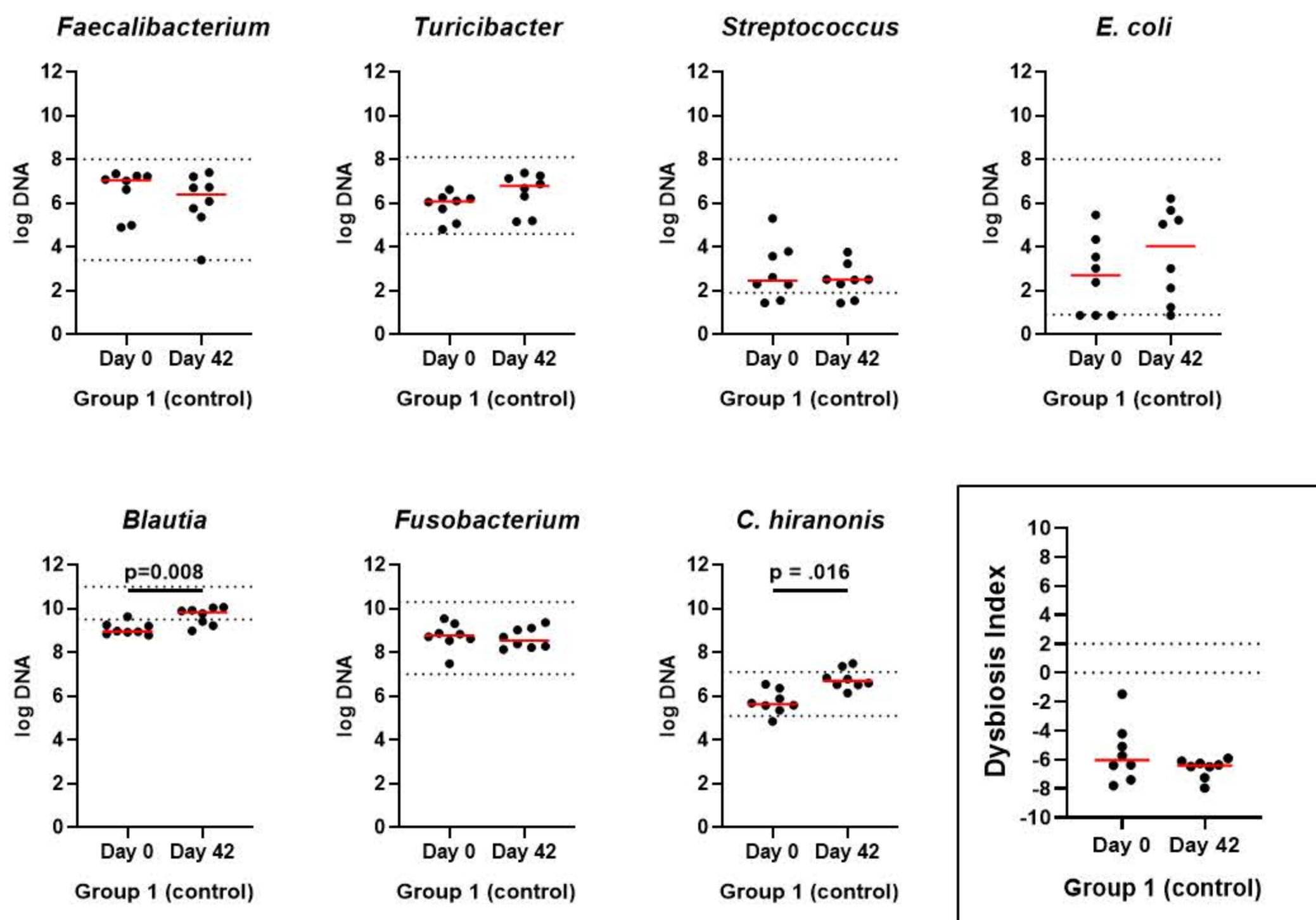**B**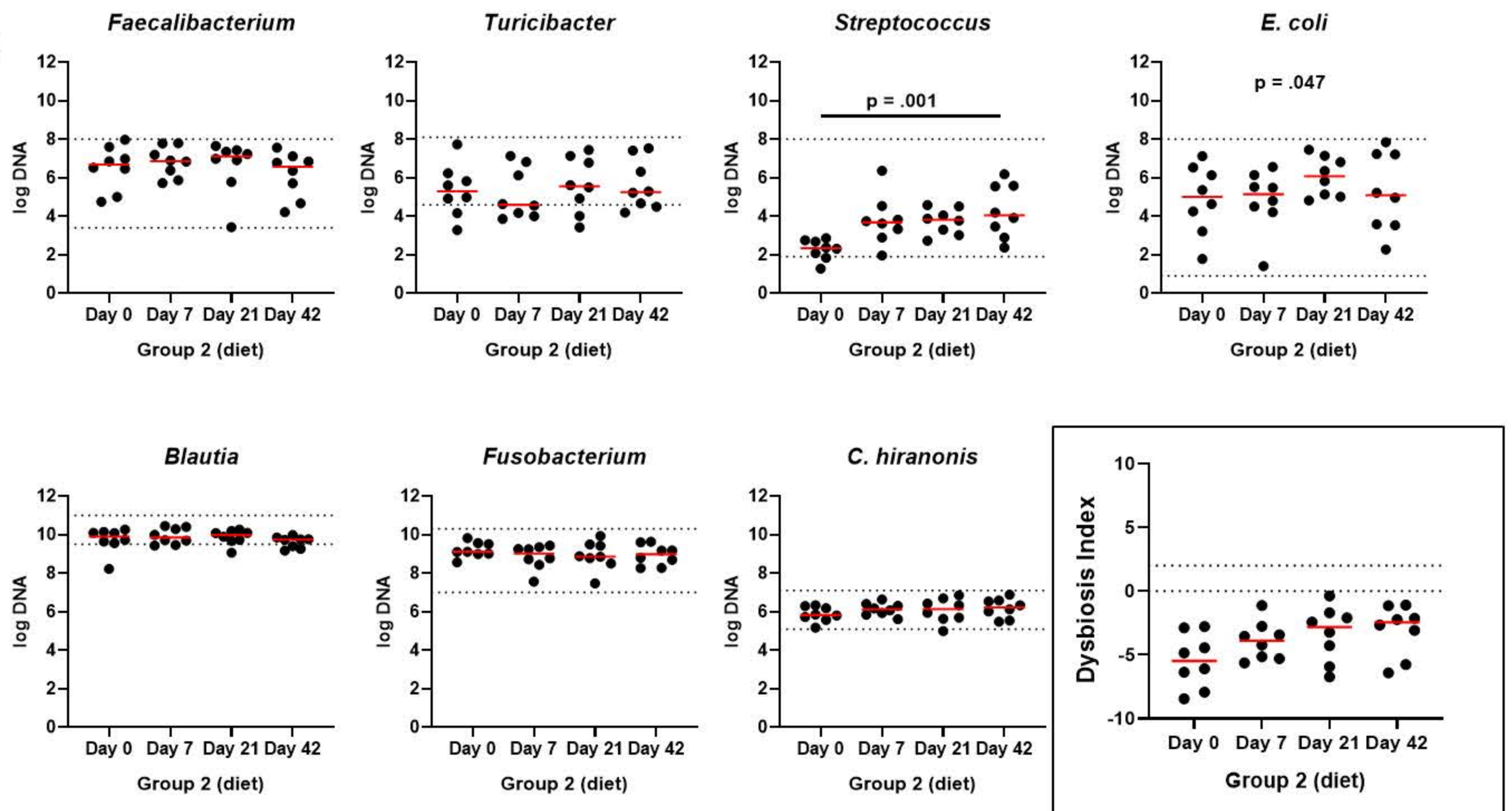**C**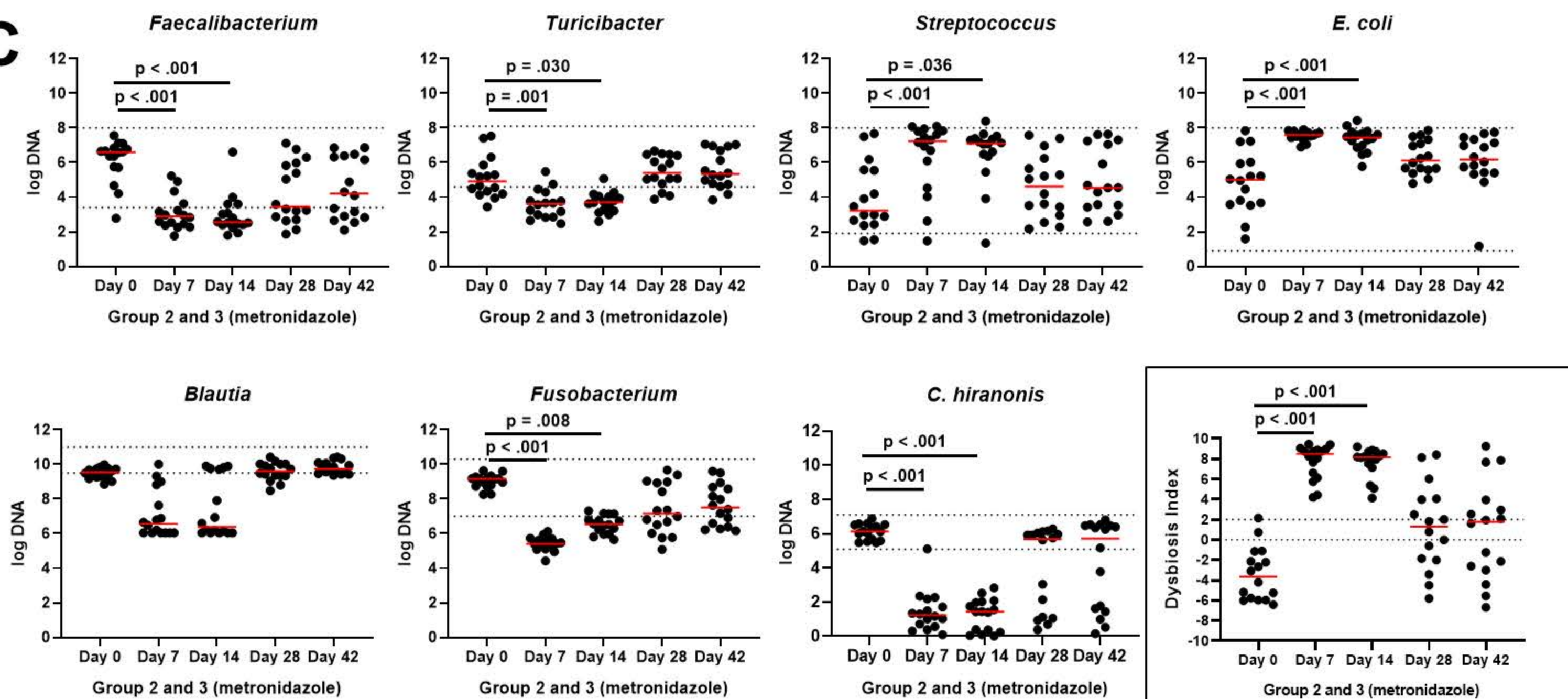

Supplement: Supplementary file 13 — Supplementary Figure S4. qPCR for Faecalibacterium, Turicibacter, Streptococcus, E. coli, Blautia, Fusobacterium, Clostridium hiranonis, and calculated fecal dysbiosis index for (A) group 1 (control), (B) group 2 during the hydrolyzed protein diet trial, and (C) groups 2 and 3 during the metronidazole trial. Results are expressed in logDNA, and dotted lines indicate the reference intervals. Fecal dysbiosis index values below zero indicate normobiosis, values between 0 and 2 are considered equivocal, and values above 2 indicate dysbiosis. [file JVIM-34-1853-s013.pdf]
