# Supplementary material for: Effects of metronidazole on the fecal microbiome and metabolome in healthy dogs
Source: J Vet Intern Med. 2020 Aug 28;34(5):1853–66. doi: 10.1111/jvim.15871 (PMC7517498; doi:10.1111/jvim.15871)
Supplement: Supplementary file 14 — Supplementary Table S1. Signalment of dogs enrolled [file JVIM-34-1853-s014.pdf]

Supplementary Table 1: Signalment of dogs enrolled

Breed	Sex	Age (years)	Weight (pounds)	Original diet	Medications/nutraceuticals	Group
Golden	CM	1	65	Purina JM	Carprofen	1
Pomeranian	SF	2	3	ProPlan Sport		1
Dachshund/terrier	CM	4	26	DRM		1
Dachshund/terrier	CM	4	11	DRM		1
Shepherd mix	CM	3	40	Sci Diet Ideal Bal active formula		1
Terrier mix	SF	3	24	ProPlan Lamb&Rice		1
Hound mix	SF	1	65	Sci Diet Lamb&Rice		1
Mix	CM	3	45	ProPlan		1
	Male: 5	Mean: 2.6	Mean: 34.9			
Vizsla	CM	2	60	Purina Lamb&Rice		2
Terrier mix	CM	2	14	Sci Diet Lamb&Rice		2
Mix	CM	3	20	ProPlan		2
Bull terrier	SF	4	45	ProPlan Sens Skin&Stomach	Glucosamine, chondroitin, trimeprazine, prednisolone, psyllium, Famotidine	2
Lab mix	SF	2	50	j/d or JM	Glucosamine, chondroitin, Famotidine	2
Dachshund	CM	6	14	Ideal Balance		2
Mastiff	F	6	100	ProPlan Sport		2
Mastiff	F	6	100	ProPlan Sport		2
	Male: 4	Mean: 3.9	Mean: 50.4			
Terrier mix	SF	4	14	Hills c/d		3
Terrier mix	SF	3	18	Hills c/d		3
Mix	SF	3	20	DH/ProPlan Weight Management		3
Lab mix	M	3	65	Merrick		3
Husky	CM	2	68	Wellness		3
Beagle mix	SF	4	20	TD/SciDiet Salmon		3
Mix	SF	1	40	TD/SciDiet Salmon		3
Mix	SF	2	35	TD/SciDiet Salmon		3
	Male: 2	Mean 2.8	Mean: 35.0			
